# Covalently Active Metabolites of Bisphenol A Analogs by Mass Spectrometry Diagnostic Ions: Possible Mechanisms of Their Toxicity

**DOI:** 10.1021/acs.chemrestox.5c00417

**Published:** 2026-01-06

**Authors:** Quan He, Xiaolan Hu, Xue Li, Na Li, Jian-Lin Wu

**Affiliations:** † Faculty of Chinese Medicine & State Key Laboratory of Mechanism and Quality of Chinese Medicine, 58816 Macau University of Science and Technology, Avenida Wai Long, Taipa, Macau SAR 999078, China; ‡ College of Environment and Climate, Institute of Mass Spectrometry and Atmospheric Environment, Guangdong Provincial Key Laboratory of Speed Capability Research, 47885 Jinan University, Guangzhou 510632, China

## Abstract

Bisphenol A analogs (BPs), used as BPA alternatives, have drawn great concerns due to their potential adverse effects. Studies have shown that reactive metabolites (RMs) formed in vitro and in vivo could covalently bind to nucleophilic macromolecules to elicit toxicity. However, the bioactivation potential of BPs and their capacity to covalently modify amino acid residues within proteins have been poorly characterized. Thus, this study systematically characterized the metabolic activation of eight BPs and their reactivity toward cysteine. Using *N*-acetylcysteine (NAC) as a trapping agent to capture RMs, we developed a novel nontargeted fragment screening strategy for cysteine adduct identification and mechanistic exploration. Integrating calculated electron affinity results, mechanistic analyses revealed a common activation pathway across multiple BPs involving oxidation, ipso-addition, and ipso-substitution. Also, the abundances of cysteine adducts correlated with metabolic rates of individual BPs, underscoring structure–reactivity relationships. These results provided critical mechanistic insight into BPs bioactivation, implicating their potential toxicity risk and supporting environmental risk evaluation.

## Graphical abstract

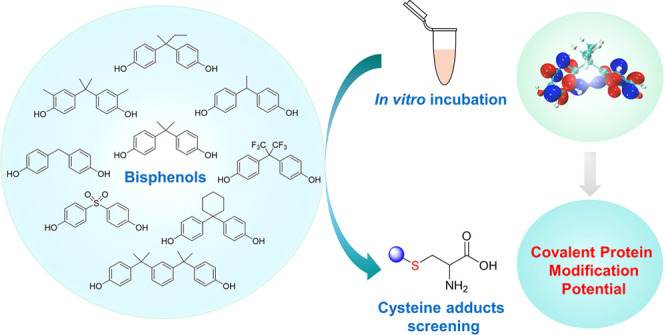

## 1 Introduction

Bisphenol A (BPA), recognized as an endocrine-disrupting chemical, has been extensively used in the production of polycarbonate plastics and epoxy resins, leading to various adverse health effects in humans.^1–3^ In response to these health concerns, several countries have banned the use of BPA.^4^ Subsequently, a variety of BPs have emerged as substitutes for BPA in various applications. These include bisphenol B (BPB), bisphenol C (BPC), bisphenol E (BPE), bisphenol F (BPF), bisphenol AF (BPAF), bisphenol S (BPS), bisphenol Z (BPZ), bisphenol M (BPM), and others.^5^ The widespread use of BPs has led to their pervasive detection in different environmental matrices, food, and human body fluids.^6–8^ Accumulated studies showed that some BPs exhibited endocrine-disrupting effects, neurotoxicity, reproductive toxicity, cytotoxicity, and genotoxicity.^9–12^ Moreover, numerous studies have demonstrated that BPB, BPS, BPAF, and BPF exert toxicities that are equivalent to or even greater than those of BPA.^13–15^ Therefore, it is essential to further evaluate and compare the risks associated with BPs to ascertain the safety of the BPA replacements.

Biotransformation is central to determining the toxicity of xenobiotics within organisms and is becoming more critical in environmental risk assessment.^16,17^ While the biotransformation of xenobiotics via human cytochrome P450 enzymes can reduce their toxicity by facilitating their clearance from organisms, it can also give rise to the formation of RMs that may exhibit higher toxicity than the prototype compounds.^17,18^ Moreover, RMs possess intrinsic chemical reactivity that can covalently bind to macromolecules, thereby triggering serious toxicity.^19^ Phenolic endocrine-disrupting chemicals, such as triclosan and BPA, serve as examples.^19,20^ Our prior research demonstrated that BPA could be bioactivated into RMs to covalently bind to cysteine residues in proteins. BPA RM modification on oxidative stress-related proteins, including superoxide dismutases (SOD), catalase (CAT), and glutathione S-transferases (GST), was an unignorable factor for its hepatotoxicity.^20^ BPs structurally similar to BPA could form RMs during bioactivation,^21^ suggesting that they might covalently bind to proteins too. Neglecting these metabolic pathways may result in an underestimation of their potential detrimental impacts on human health. Thus, the measurement of metabolic activation of BPs toward cysteine is essential for predicting the toxicity of contaminants.

The covalent binding studies in vitro, using trapping agents to trap compound-related RMs, have been used as a part of an integrated method to predict the risk of toxicity. N-acetylcysteine (NAC) and glutathione (GSH) have been widely employed to determine the intrinsic reactivity of electrophilic groups.^22^ Trapping experiments utilizing NAC or GSH generate stable adducts suitable for structural characterization via liquid chromatography–mass spectrometry (LC–MS) analysis. Notably, the rapid reaction kinetics may indicate a potential toxicity risk due to the propensity for irreversible covalent binding to proteins. This approach provides mechanistic insights into contaminant metabolic activation pathways for toxicity prediction and avoids the use of substances, enabling targeted structural modifications designed to block or minimize the formation of protein adducts, thereby providing a new avenue for reducing the toxicity of pollutants.

To investigate the potential reactivity of BPs toward cysteine residues, we exploited NAC as a trapping reagent to capture the BP RM, forming stable cysteine adducts for MS analysis. A novel nontargeted screening strategy was developed to identify these adducts. Furthermore, the selectivity of BP RM toward the cysteine residue was validated at the small peptide level. Also, the underlying reaction pathways were elucidated. Notably, the relative abundance of cysteine adduct formation was in correlation with the metabolic rates of the corresponding BPs. Results provide critical insights into covalent-binding-mediated toxicity mechanisms of contaminants.

## 2 Experimental Section

### 2.1 Chemicals and Materials

Bisphenol A (BPA) with a purity of 99% was obtained from J&K (Beijing, China). Bisphenol B (BPB, 98%), bisphenol C (2,2-Bis­(4-hydroxy-3-methylphenyl)­propane, BPC, 99%), bisphenol E (BPE, 98%), bisphenol F (BPF, 98%), bisphenol AF (BPAF, 99%), bisphenol S (BPS, 98%), bisphenol Z (4,4′-(cyclohexane-1,1-diyl) diphenol, BPZ, 99%), bisphenol M (1,3-bis­(2-(4-hydroxyphenyl)-2-propyl)­benzene, BPM, 99%), *N*-acetyl cysteine (NAC), and glutathione (GSH) were purchased from Sigma-Aldrich (St. Louis, MO, USA). Pooled human liver microsomes and nicotinamide adenine dinucleotide phosphate (NADPH) generating solutions A and B were purchased from BD Gentest (San Jose, CA, USA). Methanol (MeOH, HPLC grade), acetonitrile (ACN, HPLC grade), and acetone (HPLC grade) were purchased from Anaqua Chemicals Supply Inc. Limited (Houston, TX, USA). Deionized water was produced by a Milli-Q system (Millipore Corporation, Billerica, MA, USA).

### 2.2 Incubation of BPs and Amino Acid in Microsomes

Compound (50 μM) was incubated with NAC or GSH (5 mM) in potassium phosphate buffer (0.1 M, pH 7.4) containing microsomes (1.0 mg/mL) and NADPH-generating solutions A and B at 37 °C, with a total incubation volume of 400 μL. Aliquots (50 μL) were collected at 0, 30, 60, 120, 240, and 480 min and then quenched by five volumes of ice-cold ACN. After vortexing, the mixtures were centrifuged at 15,000 g for 10 min at 4 °C, and the supernatants were collected and evaporated to dryness under nitrogen. The residues were reconstituted in 50 μL of 50% MeOH in water. After centrifugation, the samples were analyzed by UHPLC-Q-TOF-MS/MS. Parallel controls without microsomes were also conducted to assess the direct interaction between compounds and NAC or GSH.

### 2.3 Competition Experiments

A mixture of BPA, BPB, BPC, BPE, BPF, and BPZ in a 1:1:1:1:1:1 ratio (50 μM) was incubated with GSH in the presence of microsomes (1.0 mg/mL) and NADPH-generating solutions A and B in potassium phosphate buffer solution (pH 7.4; 0.1 M) at 37 °C. The total incubation volume was 400 μL. Incubation solutions were collected at 0 and 480 min. The reactions were quenched by five volumes of ice-cold ACN containing naringenin (100 ng/mL) as the internal standard (IS). Then, the procedure was the same as those used to study the incubation of BPs with GSH. A semiquantitative analysis was employed to rank the adduct formation propensity of BPs. The peak area of each detected GSH adduct was first normalized against the peak area of IS to obtain a corrected value (corrected area adduct_*i*_ = area adduct_*i*_/area_*IS*_). The corrected values of all GSH adducts derived from the same BPs were summed to represent the total adduct burden. The relative abundance of each GSH adduct (i) was calculated as its percentage contribution to the total corrected value for % adduct_*i*_ = [corrected area adduct_*i*_/∑(corrected area adduct_1_ + corrected area adduct_2_ + ···)] × 100%.

### 2.4 Liquid Chromatography–Mass Spectrometry Analysis

The separation of NAC conjugates and GSH conjugates was carried out with an Agilent 1290 Infinity UHPLC equipped with a binary pump and 6545 Q-TOF-MS/MS system with Waters ACQUITY UHPLC BEH C18 column (2.1 × 100 mm, 1.7 μm) at room temperature (25 °C). The flow rate was 0.3 mL/min. The mobile phase consisted of 0.1% (v/v) formic acid (A) and 0.1% (v/v) formic acid in acetonitrile (B) with the following gradient: 0–0.5 min, 5% B; 0.5–4 min, 5% to 20% B; 4–6 min, 20% to 40% B; 6–8 min, 40% to 60% B; 8–10 min, 60% to 95% B; 10–11.9 min, 95% B; and 11.9–12 min, 5% B. The injection volume was 1 μL. The MS was operated in negative ion modes for NAC conjugates and positive ion modes for GSH conjugates. The MS parameters were set as gas temperature at 325 °C, dry gas flow at 9 L/min, sheath gas temperature at 350 °C, sheath gas flow at 11 L/min, nebulizer pressure at 35 psig, capillary voltage at 3500 V, and nozzle voltage at 1500 V for negative ion mode and 500 V for positive ion mode. Mass spectra were collected over a range of 100–1000 *m*/*z*. For MS/MS analysis, both automated and targeted acquisitions were employed with a scan range of 50–1000 *m*/*z* with a collision cell energy of 5–20 eV.

### 2.5 Computational Methods

The chemical structures of BPs were calculated by density functional theory (DFT) utilizing the ORCA program (version 5.0.4).^23^ Geometry optimizations were performed at the B3LYP/6-311+G**level. Solvent effects were incorporated via the solvation model based on density (SMD), with single-point energy calculations conducted at the M06-2X/6-311+G** level on the gas-phase optimized geometries. Electron affinity (EA) and condensed Fukui function index (*f* ^+^) were calculated as DFT reactivity descriptors to predict the reactivity and regioselectivity of BPs toward the cysteine residue.

#### 2.5.1 Electron Affinity Calculations

The adiabatic gas-phase electron affinities were computed through independent geometry optimizations of the neutral quinone (N electrons) and its corresponding semiquinone radical anion (N + 1 electrons). The electron affinity (EA) was determined as^24^

$$EA=E_{optimized\;neutral}\left(N\right)-E_{optimized\;anion}\left(N+1\right)$$

#### 2.5.2 Local Nucleophilicity Index Calculations

The global nucleophilicity index (*N*) was calculated using the highest occupied molecular orbital (HOMO) energy, defined as

$$N=E_{HOMO\left(Nu\right)}\;\left(eV\right)-E_{HOMO\left(TCE\right)}\;\left(eV\right)$$

where tetracyanoethylene (TCE) was used as the reference molecule for its exceptionally low HOMO energy.^25^

Since the global nucleophilicity (*N*) describes reactivity at the molecular level, a local nucleophilicity index [*N*(*r*)] was employed to assess site selectivity in amino acids.^26,27^

$$N\left(r\right)=Nf^{-}\left(r\right)\left(=N^{-}\left(r\right)\right)$$

with *f* ^–^(r) the Fukui function for electrophilic attack.^28^ This Fukui function is condensed to atoms to calculate the Fukui index. In this study, atomic populations were obtained with the NBO charge method.^29^ For the analysis of electrophile–nucleophile interactions, *N* ^–^(*r*) is a better reactivity descriptor than the corresponding Fukui function, because the local electrophilicity index is a product of a global (*N*) and a local index [*f* ^–^(*r*)]. The condensed-to-atom *k* variant is defined as

$$N_{k}=Nf_{k}^{-}$$

The Fukui function is a very important concept in the conceptual DFT, and it has been widely used in the prediction of reactive sites.^30^ Fukui function is defined as follows

$$f\left(r\right)={\left[\frac{\partial \rho \left(r\right)}{\partial N}\right]}_{V}$$

where ρ­(*r*) was the electron density at a point *r* in space, *N* was the electron number in the present system, the constant term *v* in the partial derivative was the external potential. In the condensed version of the Fukui function, the atomic population number was used to represent the amount of electron density distribution around an atom. The condensed Fukui function could be calculated as

$$nucleophilic\;attack:\;f_{k}^{+}=q_{N}^{k}-q_{N+1}^{k}$$

$$electrophilic\;attack:\;f_{k}^{-}=q_{N-1}^{k}-q_{N}^{k}$$

$$radical\;attack:f_{k}^{0}=\left(q_{N-1}^{k}-q_{N+1}^{k}\right)/2$$

where *q* ^*k*^ was the atom charge of atom *k* at the corresponding state, and the values of the Fukui index of the reactive sites were usually larger than other regions.

### 2.6 Statistical Analysis

Results were presented as means ± SD. Statistical analysis was conducted using GraphPad Prism 9 software (GraphPad, Inc. USA). The Student’s *t*-test was used to compare the levels of BPs between the two groups (0 and 4 h). Also, one-way analysis of variance followed by Tukey’s honestly significant difference was performed for multiple group analyses. *P* values of <0.05 are regarded as indicative of statistical significance.

## 3 Results

### 3.1 Evaluating the Transformation of BPs in Liver Microsomes

The metabolic transformation of BPs was evaluated in the microsomal system. Following a 4 h incubation, the concentrations of each BP were significantly reduced (*p* < 0.01). The metabolic efficiencies of BPA, BPB, BPC, BPE, BPF, BPAF, BPS, BPZ, and BPM were 59.2, 50.1, 25.2, 64.3, 62.3, 6.8, 4.0, 12.4, and 5.4%, respectively (Figure 1A). To investigate the potential influence of electronic properties on metabolic stability, the EA was calculated for each BP. EA was defined as the energy difference between the optimized geometry of neutral quinone and its corresponding semiquinone (SQ) radical anion.^24^ In comparison to BPA, the calculated EAs of BPB, BPC, BPE, and BPF clustered within a narrow range of −8.81 to −11.49 kcal/mol. In contrast, BPS exhibited a distinct positive EA value of 9.10 kcal/mol (Table 1). All tested BPs retained metabolic susceptibility in microsomal systems, despite structural variations at the ipso-position that modulate metabolic efficiency.

**Figure 1:**
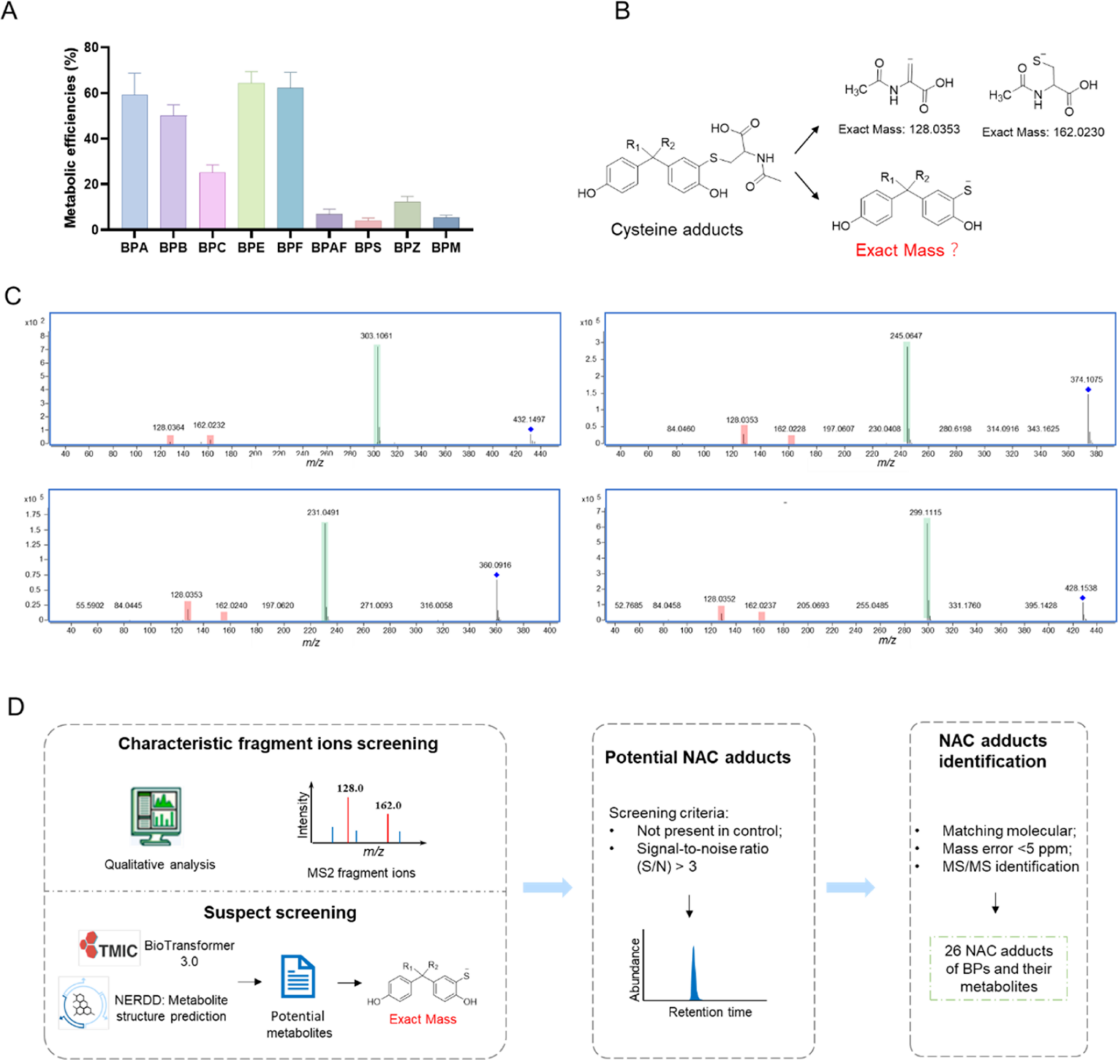
(A) Metabolic efficiencies of BPs in liver microsomal systems. (B) MS/MS fragmentation characteristics of NAC adducts formed from BPs or their metabolites; (C) representative MS/MS spectra of NAC adducts, and (D) workflow for screening NAC adducts.

**Table 1:** Calculated B3LYP Energy and EA of Selected BPs^a^

	B3LYP energy (Hartrees)	EA (Hartrees)	EA (kcal/mol)
BPA	–731.590039	–0.0169	–10.5911
BPA SQ radical	–731.573161		
BPB	–770.879076	–0.0140	–8.8052
BPB SQ radical	–770.865044		
BPC	–810.186078	–0.0183	–11.4897
BPC SQ radical	–810.167768		
BPE	–692.299907	–0.0159	–9.9573
BPE SQ radical	–692.284039		
BPF	–653.007612	–0.0160	–10.0200
BPF SQ radical	–652.991644		
BPAF	–1327.265489	–0.0018	–1.1339
BPAF SQ radical	–1327.263682		
BPS	–1162.341541	0.0145	9.0958
BPS SQ radical	–1162.356036		
BPZ	–848.343666	–0.0806	–50.5735
BPZ SQ radical	–848.263072		
BPM	–1080.488977	–0.0102	–6.3724
BPM SQ radical	–1080.478822		

a SQ radical means the semiquinone radical of the corresponding quinone.

### 3.2 Method Development for Cysteine Adduct Discovery

The above results prompted us to investigate the potential covalent modification of proteins by BPs following biotransformation. To evaluate the reactivity of BPs and their RMs toward nucleophilic cysteine residues in proteins, NAC was utilized as a trapping agent in a microsomal incubation system. We developed an untargeted UHPLC-Q-TOF-MS/MS-based metabolomics approach to explore the potential cysteine adducts.

Considering that NAC adducts share a common NAC moiety, identifying their characteristic fragment ions could provide an efficient, rapid, and accurate method for profiling NAC adducts. Our previous studies have shown that different NAC adducts could produce characteristic fragment ions resulting from the cleavage of the C–S bond, either between the substrate and NAC moiety or within the NAC moiety.^20,31^ In negative ion mode, these fragments correspond to deprotonated ions of NAC derivatives at *m*/*z* 162.0 and 128.0, as well as the substrate or its metabolites with the sulfur atom of NAC attached (Figure 1B,C). To systematically identify cysteine adducts, the metabolites of BPs were first predicted by BioTansformer 3.0 and New E-Resource for Drug Discovery software. Then, the possible sulfhydryl-containing BPs or their metabolites are predicted based on the covalent binding mechanisms.^31^ We used these characteristic fragment ions to screen BP-derived NAC adducts in the microsomal system (Figure 1D). In total, 26 new NAC adducts were extracted and confirmed by MS/MS spectra (Table S1 and Figures S1–S8).

Taking BPB as an example, which structurally differs from BPA only by an extra methyl group on the central carbon, four NAC adducts were detected (Table S1). No corresponding cysteine adducts were detected in NADPH and microsomal free incubations, indicating that BPB could not bind to cysteine without prior metabolism. The structures of these adducts were tentatively determined through the elucidation of MS and MS/MS analysis. BC1 showed the deprotonated molecular ion [M – H]^−^ at *m*/*z* 402.1385, corresponding to the chemical formula of C_21_H_25_NO_5_S, which was determined by the characteristic NAC fragment ion at *m*/*z* 128.0357. And the fragment ion at *m*/*z* 273.0963 ([M – H – C_5_H_7_NO_3_]^−^), generated by C–S bond cleavage in the NAC moiety, confirmed NAC binding to BPB via its terminal –SH group (Figure S1A). BC2 displayed a deprotonated molecule ion [M – H]^−^ at *m*/*z* 418.1318, which was determined by the characteristic NAC fragment ions at *m*/*z* 128.0353 and 162.0243, which were 16 Da heavier than BC1 and corresponded to the chemical formula of C_21_H_25_NO_6_S. The fragment ions at *m*/*z* 289.0907 resulting from the neutral loss of C_5_H_7_NO_3_ confirmed BC2 as a monooxygenated BPB-NAC adduct (Figure S1B). BC3 exhibited a deprotonated molecular ion [M – H]^−^ at *m*/*z* 434.1289 with the chemical formula C_21_H_25_NO_7_S, as determined by a characteristic NAC fragment ion at *m*/*z* 128.0357. BC3, which was 32 Da heavier than BC1, together with the fragment ion at *m*/*z* 305.0870, was confirmed as a dioxygenated BPB-NAC adduct (Figure S1C). BC4 was determined by the sulfhydryl-containing fragment ion at *m*/*z* 125.0069 ([M – H – C_5_H_7_NO_3_]^−^) (Figure S1D). Its deprotonated molecular ion [M – H]^−^ at *m*/*z* 254.0491, corresponding to the chemical formula C_11_H_13_NO_4_S, indicated that NAC was added to BPB’s ipso-position cleavage metabolite, forming phenol-NAC adduct. The successful determination of NAC adducts of BPB and its RMs demonstrated that the diagnostic fragment-driven screening strategy enabled the rapid and efficient identification of adduct candidates from complex matrices.

This strategy was further applied to structurally diverse BPs. BPC is structurally distinguished from BPA by featuring two methyl substitutions on each phenolic ring. Three NAC adducts of BPC were detected in the microsomal incubation system (Table S1 and Figure S2). CC1 (*m*/*z* 416.1547) and CC2 (*m*/*z* 432.1497) were determined by the characteristic NAC fragment ion at *m*/*z* 128.035, corresponding to NAC adducts formed from BPC and monooxygenated BPC, respectively. CC3 (*m*/*z* 268.0664) was determined by the sulfhydryl-containing a fragment ion at *m*/*z* 139.0224, corresponding to NAC adducts derived from the ipso-position cleavage metabolite, 2-methylphenol. Compared to BPA, BPE lacks a methyl group on the central carbon. Three NAC adducts were detected with the deprotonated molecular ions at *m*/*z* 374.1075, 390.1024, and 254.0495, respectively, corresponding to NAC adducts of BPE (EC1), monooxygenated BPE (EC2), and phenol (EC3) (Figure S3). BPF is distinct from BPA solely due to the absence of two methyl groups on the central carbon atom. Five NAC adducts were determined, formed from BPF (FC1), monooxygenated BPF (FC2), dioxygenated BPF (FC3), phenol (FC4), and dihydroxybenzene (FC5) (Figure S4). BPAF is characterized by two trifluoromethyl groups on the central carbon atom. One NAC adduct was detected, which was determined by characteristic NAC fragment ions at *m*/*z* 128.0349 and 162.0239, corresponding to NAC adducts of monooxygenated BPAF (AFC1, Figure S5). BPS differs structurally from BPA by containing a sulfone group (−SO_2_^–^) as its central linker rather than the dimethylmethylene group. Three NAC adducts formed from monooxygenated BPS (SC1), dioxygenated BPS (SC2), and dihydroxybenzene (SC3) were detected (Figure S6). BPZ is structurally distinguished from BPA via replacement of the dimethylmethylene bridging group with a saturated cyclohexane moiety as its central linker. Four NAC adducts formed from BPZ (ZC1), monooxygenated BPZ (ZC2 and ZC3), and phenol (ZC4) were detected (Figure S7). BPM features a central benzene ring substituted at the 1,3-positions with two isopropyl-linked *p*-hydroxyphenyl groups. Three NAC adducts formed from BPM (MC1), mono-oxygenated BPM (MC2), and dioxygenated BPM (MC3) (Figure S8). These findings demonstrated that this screening strategy enable specific identification of cysteine adducts formed from BPs and their metabolites within the complex microsomal matrix.

### 3.3 Validation of BP Reactivity and Selectivity

To further evaluate the reactivity and selectivity of BPs, glutathione (GSH) as a small peptide with a thiol group and amino group was employed as a trapping agent in hepatic microsomal incubations. Forty-seven GSH conjugates bound to the thiol group were detected, while no conjugates bound to the amino group were observed under the present conditions, indicating that BPs have no or low reaction activity with the amino group. These GSH conjugates generated characteristic fragment ions during collision-induced dissociation. In the positive mode, GSH conjugates readily undergo cleavage, resulting in the neutral losses of glycine (75 Da, C_2_H_5_NO_2_) and pyroglutamic acid (129 Da, C_5_H_7_NO_3_) ions, accompanied by the formation of corresponding fragment ions at *m*/*z* 76 and 130, respectively. Additionally, fragment ions resulting from cleavage of the cysteinyl C–S bond within the GSH moiety were also observed. Seven GSH conjugates, BG1-BG7, were detected from the BPB incubation system (Table 2). BG5 showed the protonated molecular ion at *m*/*z* 456.1814, corresponding to the chemical formula of C_20_H_29_N_3_O_7_S. The fragment ions of *m*/*z* 327.2020 generated from the elimination of C_5_H_7_NO_3_ provided the evidence that GSH bound to BPB metabolite, 4-(butane-2-yl) phenol (Figure S9E). Others showed the protonated molecular ions at *m*/*z* 548.2083, 564.2031, 400.1182, 416.1123, 472.1748, and 470.1595, respectively, corresponding to GSH conjugate adducts formed with BPB (BG1), monooxygenated BPB (BG2), phenol (BG3), dihydroxybenzene (BG4), 4-(butane-2-yl)­dihydroxybenzene (BG6), and 4-(butene-2yl)­dihydroxybenzene (BG7) (Figures 2A and S9). In addition, the formation of these conjugates exhibited a time-dependent increase during the reaction period (Figure 2B). Based on peak areas, BG1, BG3, and BG5 were the major conjugates in the in vitro incubation system. The condensed Fukui function (*f* ^+^) was applied to analyze the regioselectivity of GSH nucleophilic attacks on BPs substrates. The *f* ^+^ value of BPB reveals that C5 (*f* ^+^ = 0.118) was the preferred site for nucleophilic addition (Figure 2C). Notably, the *f* ^+^ value at C5 of BPB is higher than that of the corresponding site in BPA (*f* ^+^ = 0.103), indicating that the additional methyl group in BPB enhances the reactivity of the phenolic ring. Based on these results, we proposed a potential CYP450-mediated bioactivation pathway (Figure 3A), including oxidation, ipso-addition, and ipso-substitution reactions, similar to the pathway observed for BPA.^20^

**Table 2:**
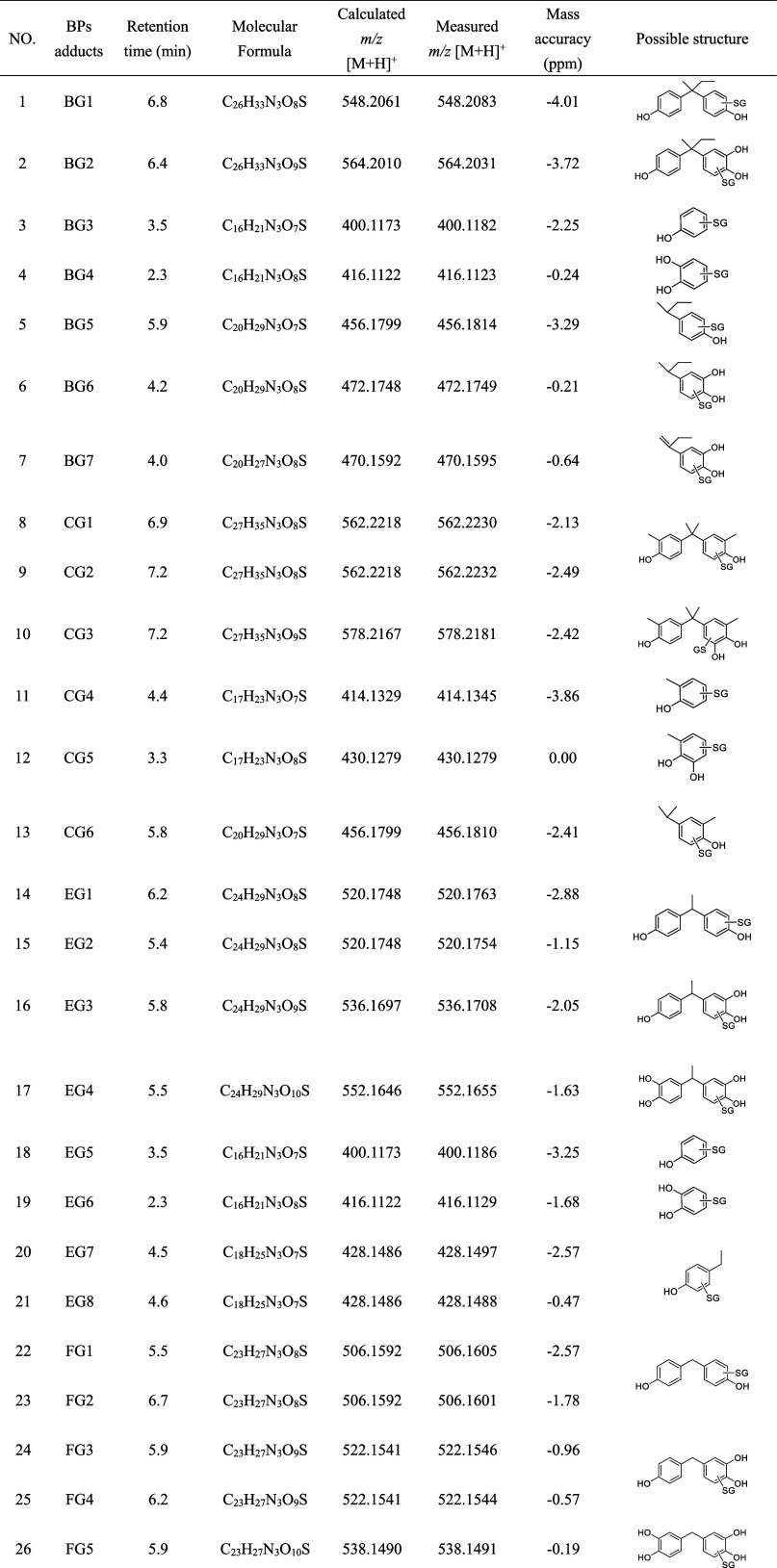

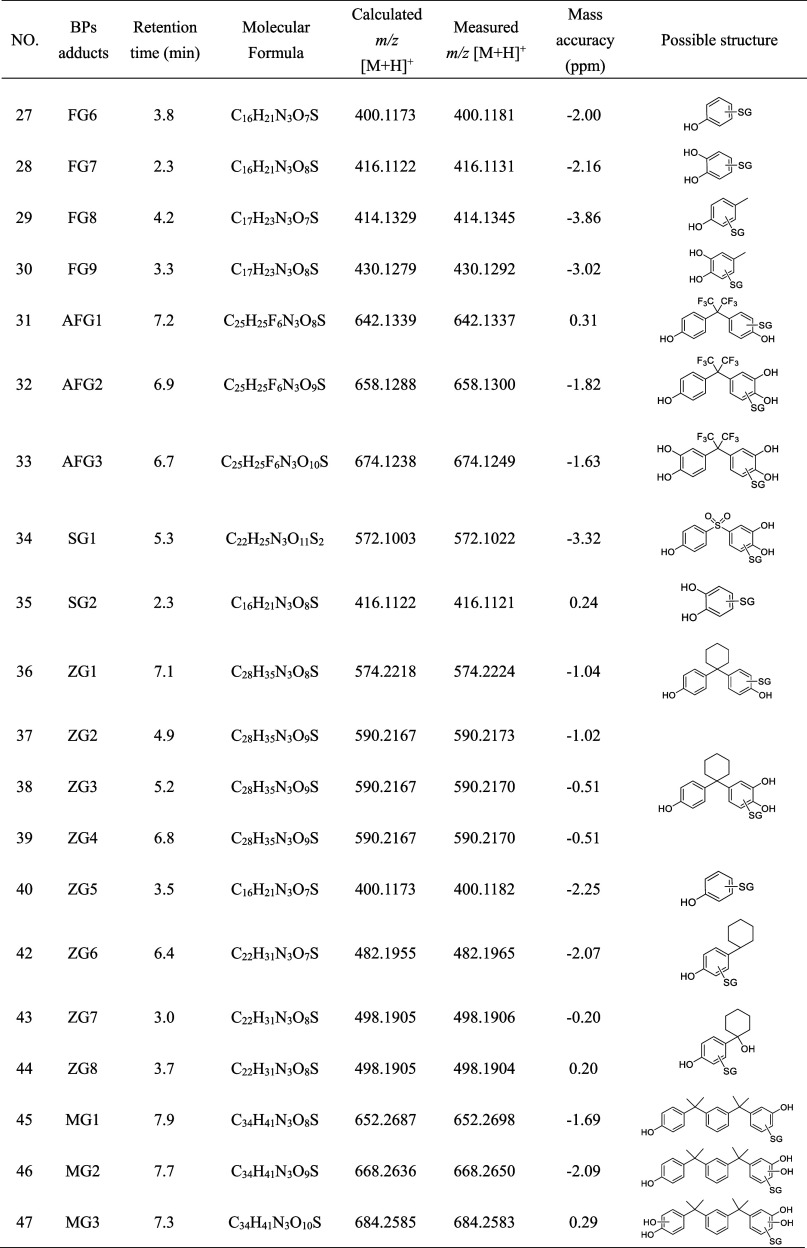
BPs Covalently Bind with GSH after Bioactivation

**Figure 2:**
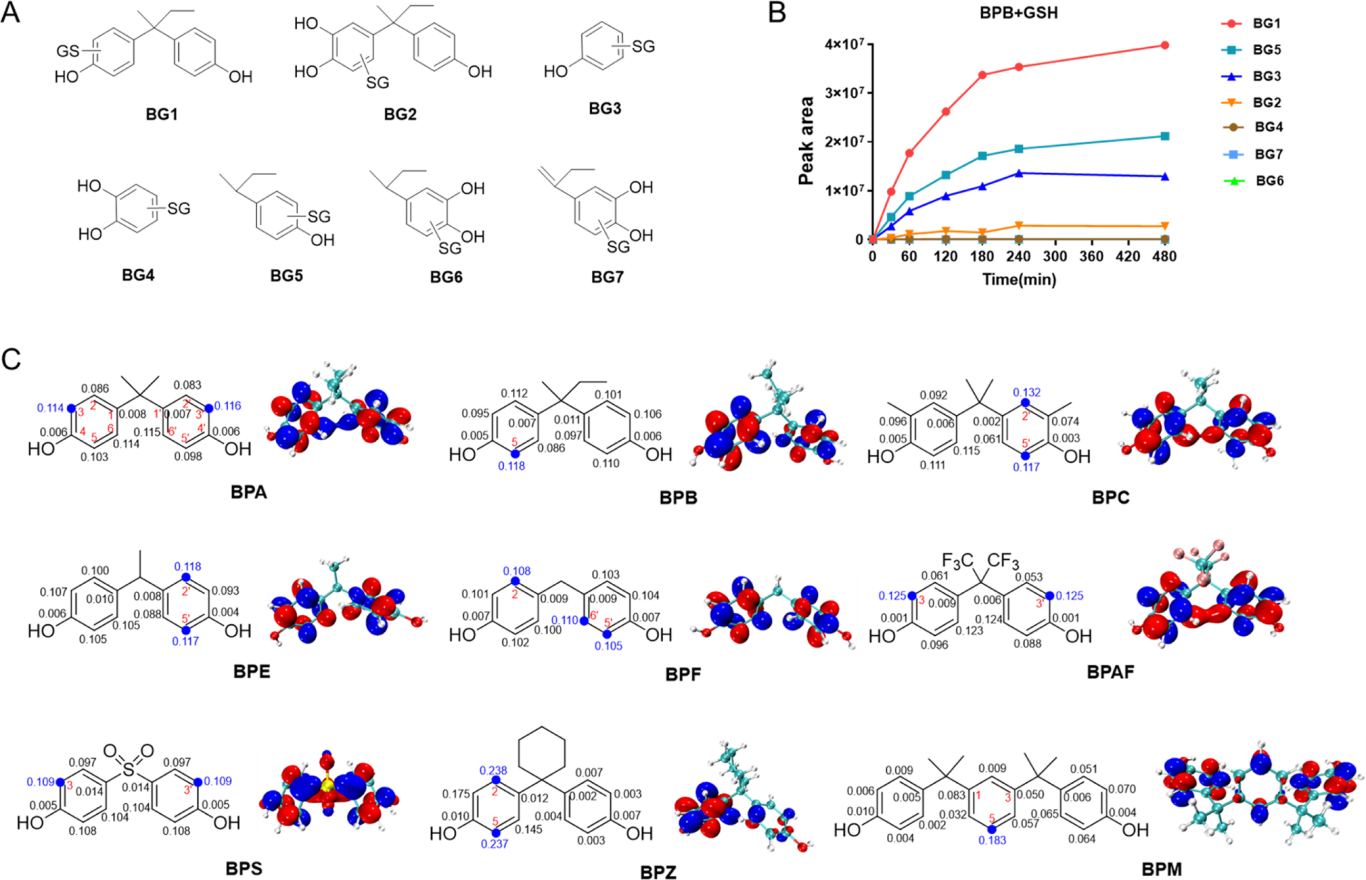
(A) Possible structure and (B) time-course changes of GSH conjugates formed with BPB and its metabolites in microsomes; (C) Fukui index (*f* ^+^) computed for BPs.

**Figure 3:**
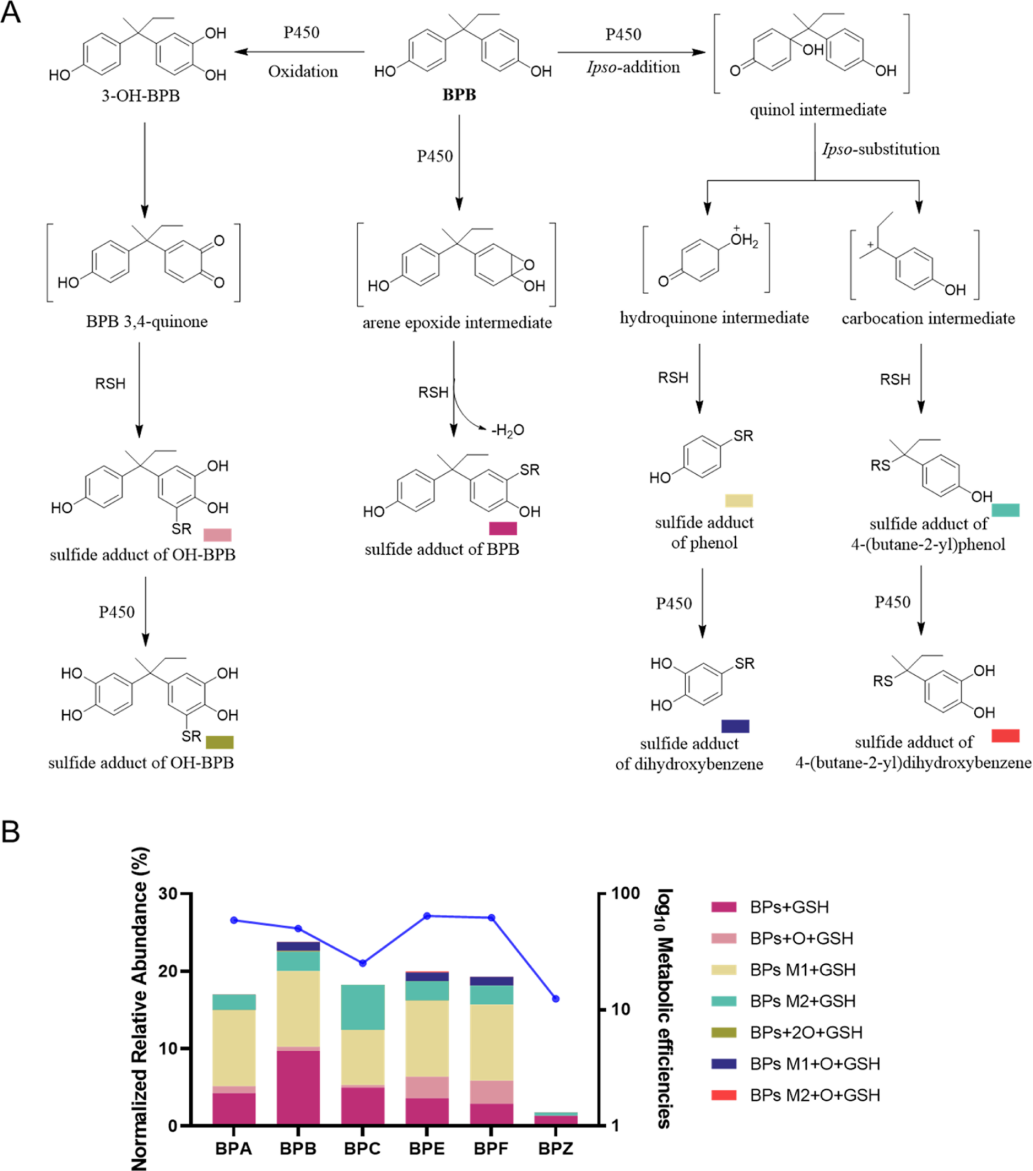
(A) Proposed metabolic biotransformation of BPB. (B) GSH conjugates levels of BPs and their metabolites after coincubation in a microsomal system.

According to the metabolite pathway, the bioactivation potential of BPC, BPE, BPF, BPAF, BPS, BPZ, and BPM was also evaluated using the GSH trapping assay in the presence of microsomes. For BPC, six GSH conjugates were identified. CG1 and CG2, eluting at 6.9 and 7.2 min, shared a protonated molecular ion at *m*/*z* 562.2230 (C_27_H_35_N_3_O_8_S), corresponding to BPC-GSH conjugates (Table 2, Figure S10A). CG3-CG6 had the protonated molecular ions at *m*/*z* 578.2181, 414.1345, 430.1279, and 456.1810, respectively, corresponding to GSH conjugates form with monooxygenated BPC, 2-methylphenol, hydroxy-2-methylphenol, and 4-(propan-2-yl)-2-methylphenol, respectively (Figure S10). The *f* ^+^ values indicated C2′ (*f* ^+^ = 0.132) or C5′ (*f* ^+^ = 0.117) as the preferred sites for GSH nucleophilic attack on BPC, while steric hindrance should be incorporated to explain regioselectivity (Figure 2C). For BPE, eight GSH conjugates were determined, including BPE-GSH (EG1 and EG2), monooxygenated BPE-GSH (EG3), dioxygenated BPE-GSH (EG4), phenol-GSH (EG5), dihydroxybenzene-GSH (EG6), 4-ethyl-phenol-GSH (EG7), and 4-ethyl-phenol-GSH (EG8) (Figure S11). Based on the *f* ^+^ values for BPE, C2′ (*f* ^+^ = 0.118) is the electronically optimal site for nucleophilic attack. However, due to steric shielding at C2′, the attack might shift to the secondary site C5′ (*f* ^+^ = 0.117), which exhibits comparable electrophilicity (Figure 2C). For BPF, nine GSH conjugates were detected: BPF-GSH (FG1 and FG2), monooxygenated BPE-GSH (FG3 and FG4), dioxygenated BPF-GSH (FG5), phenol-GSH (FG6), dihydroxybenzene-GSH (FG7), 4-methyl-phenol-GSH (FG8), and 4-methyl-dihydroxybenzene-GSH (FG9) (Figure S12). The *f* ^+^ values identified C6′ (*f* ^+^ = 0.110), C5′ (*f* ^+^ = 0.105), and C2 (*f* ^+^ = 0.108) as sites susceptible to GSH nucleophilic attack (Figure 2C). Three GSH conjugates of BPAF were determined as BPAF-GSH (AFG1), monooxygenated BPAF-GSH (AFG2), and dioxygenated BPAF-GSH (AFG3) (Figure S13). The –CF_3_ group in BPAF creates an electron-withdrawing effect, rendering the C3 (*f* ^+^ = 0.125) and C3′ (*f* ^+^ = 0.125) positions highly electrophilic and susceptible to nucleophilic attack. Two GSH conjugates of BPS were found, including monooxygenated BPS-GSH (SG1) and dihydroxybenzene-GSH (SG2) (Figure S14). Similar to BPAF, the –SO_2_ group in BPS exhibits a strong electron-withdrawing effect, which increases the electrophilicity of C3 (*f* ^+^ = 0.125) and C3′ (*f* ^+^ = 0.125) positions. Eight GSH conjugates of BPZ were determined as BPZ-GSH (ZG1), monooxygenated BPZ-GSH (ZG2, ZG3, and ZG4), phenol-GSH (ZG5), *para*-hydroxyphenylcyclohexane-GSH (ZG6), and *para*-dihydroxyphenylcyclo-hexanol-GSH (ZG7 and ZG8) (Figure S15). While the *f* ^+^ values confirm that C2 (*f* ^+^ = 0.238) and C5 (*f* ^+^ = 0.237) are electronically preferred sites for GSH attack in BPZ, steric constraints from the bulky cyclohexane moiety must be rigorously assessed. Three GSH conjugates of BPM were detected as BPM-GSH (MG1), monooxygenated BPM-GSH (MG2), and dioxygenated BPM-GSH (MG3) (Figure S16). According to the *f* ^+^ values, the central benzene ring C5 (*f* ^+^ = 0.183) is likely to be preferentially attacked by GSH.

GSH conjugate profiling revealed that BPs shared a common metabolic activation pathway, proceeding via oxidation, ipso-addition, and ipso-substitution reactions. These results provided strong evidence that BPs could undergo bioactivation to form RMs capable of covalent adduction with cysteine residues in protein. Moreover, all GSH conjugates demonstrated time-dependent accumulation (Figures S17–S24).

### 3.4 Toxicological Implications of BPs

The formation of RMs is linked to a range of adverse effects. To compare the covalently adduct-forming potential of structurally similar BPs, including BPA, BPB, BPC, BPE, BPF, and BPZ, these compounds were coincubated with GSH at equimolar concentrations (1:1:1:1:1:1) in the microsomal system. In order to compare the relative trends in adduct formation across different BPs, semiquantitative analysis was performed by calculating the IS-corrected peak area for each GSH conjugate and determining its proportional contribution to the total conjugates. This analysis revealed the following hierarchy for covalent adduct formation capacity: BPB > BPC > BPE > BPF > BPA > BPZ (Figure 3B). The variability in metabolite profiles and relative abundances reflected differential metabolic fates among these BPs, with the relative abundances being consistent with the metabolic rates mentioned above.

## 4 Discussion

Biotransformation critically influences xenobiotic toxicity by generating RMs that are more toxic than the parent compound and capable of covalently binding to proteins, triggering serious adverse effects. We previously showed that BPA bioactivation yielded RMs that covalently bind to cellular proteins, leading to hepatotoxicity.^20^ Given that BPs shared a core diphenylmethane structure with two *para*-hydroxyl-substituted benzene rings, we hypothesized that they undergo analogous metabolic activation. To date, most biotransformation studies have focused on BPA, with only a limited number investigating other analogues such as BPF, BPAF, and BPZ.^32^ However, there is still no comprehensive and comparative analysis of biotransformation across a structurally diverse set of BPs, which hinders a thorough safety evaluation. In this study, we systematically examined the enzyme-mediated transformation of BPs and evaluated their potential for covalent binding to cysteine residues.

First, the transformation of BPs was investigated using in vitro systems. BPE and BPF exhibited comparable depletion tendency to BPA, implying that their structures are more readily metabolizable. In contrast, the markedly lower metabolic rates of BPAF and BPS might be attributed to inherent molecular stability conferred by their ipso-substituents (−CF_3_/–SO_2_), which were higher than those of linear alkyl groups.^2,33^ This is evidenced by their low B3LYP energies: −1327.27 for BPAF and −1162.34 for BPS, respectively (Table 1). Furthermore, EA calculations provided an electronic structural perspective for the differential metabolic rates of BPs. The comparable EA values of BPA, BPB, BPC, BPE, and BPF suggested analogous electron-accepting behavior, while the aberrant positive EA of BPS indicated the thermodynamic instability of its radical anion that might hinder downstream metabolic processes. Additionally, the low metabolic rate of BPZ and BPM might be attributed to steric hindrance effects. These results demonstrated that BPs exhibited metabolic capacity in microsomal systems, with their efficiency modulated by structural differences at the ipso-position.

To investigate whether BPs could covalently bind to nucleophilic cysteine residues, we initially used NAC as a trapping agent to capture RMs derived from BPs. An untargeted UHPLC-Q-TOF-MS/MS method, combining with a diagnostic fragment-driven screening strategy, was developed to explore the potential cysteine adducts. This approach enabled rapid and efficient identification of cysteine adducts formed from BPs and their metabolites within a complex microsomal matrix. Furthermore, these adducts were further validated using tripeptide GSH. Although structural differences among BPs yielded distinct adduct profiles, they possessed a common metabolic activation pathway, with the process involving oxidation, ipso-addition, and ipso-substitution reactions. Among them, BPF, with a reduced steric bulk, yielded the widest variety of cysteine adducts. In contrast, electron-deficient groups in BPAF and BPS constrained their reactivity, resulting in fewer cysteine adducts predominantly via oxidative pathways. BPM also primarily formed cysteine adducts via oxidative pathways, presumably due to steric hindrance. These results provided strong evidence that BPs could undergo bioactivation to form RMs capable of covalently binding to cysteine. This observation indicated that BPs might have the potential to form covalent bonds with cysteine residues in protein, implicating this mechanism in BP-induced toxicity. Moreover, the time-dependent accumulation of GSH conjugates confirmed the sustained generation of these reactive intermediates (Figures S17–S24), enabling persistent protein adduct formation that might drive prolonged toxicological effects. Overall, these findings provided critical mechanistic insight into the metabolic activation of BPs and their structure-dependent toxicological potential.

The significant differences in covalent adduct formation and metabolite profiles among structurally similar BPs underscored a structure-dependent metabolic divergence. These findings suggested that structural characteristics, particularly steric and electronic properties of substituents, influenced their reactivity toward GSH. Previous studies have demonstrated that BPB exhibited higher binding affinity to G protein-coupled estrogen receptor (GPER) than BPA, consistent with its stronger GPER agonistic activity, indicating the agonistic potential of BPs toward GPER might be determined by their direct binding affinity.^34^ In addition, BPB and BPE have been reported to display higher binding affinity and activity toward ER compared to BPA.^35,36^ Consistent with these receptor interactions, our results revealed that BPB and BPE formed more adducts than BPA, implying an increased capacity for protein modification. Furthermore, the covalent modifications could disrupt protein structures and/or functions, initiating a cascade of toxicological effects.^19^ Our prior work demonstrated that BPA RMs covalently modified oxidative stress-related proteins, including SOD, CAT, and GST, thereby impairing their enzymatic activities. This impairment led to the accumulation of reactive oxygen species, depletion of GSH, and elevated lipid peroxidation (MDA), ultimately leading to hepatotoxicity.^20^ Additionally, sustained adduct formation across all BPs indicated prolonged adverse effects. Collectively, these findings suggested that BPs might elicit a higher toxicological risk than BPA and were therefore unlikely to be safe alternatives. Moreover, future studies are warranted to identify and characterize protein targets of BPs to enable a more conclusive hazard assessment.

In conclusion, this study established a simple, selective, and effective diagnostic fragment-driven strategy for screening cysteine adducts. The successful application of this strategy demonstrated that BPs could covalently bind to cysteine residues, suggesting a potential mechanism for their toxicity. We identified key reaction products and elucidated pathways for cysteine adduct formation across structurally diverse BPs. These transformations involved the metabolic activation of BPs, hydroxylation, and carbon bridge cleavage, with all resulting metabolites undergoing covalent conjugation to cysteine residues. The in vitro covalent binding studies provided a predictive framework for assessing the potential to target cysteine, implicating the toxicity risks of environmental contaminants. Furthermore, by identifying chemicals prone to forming protein adducts, this approach enabled proactive intervention in the use of high-risk substances to mitigate adverse health effects.

## Supplementary Material



## Notes

BPA analogues covalently bind with NAC after bioactivation, MS/MS spectrum and possible structure of BPs adducts with NAC and GSH, and extracted ion chromatograms, possible structure, and time-course changes of GSH conjugates formed with BPs and their metabolites in microsomes (PDF)

## Abbreviations

BPA: bisphenol A
BPs: BPA analogs
BPB: bisphenol B
BPC: bisphenol C
BPE: bisphenol E
BPF: bisphenol F
BPAF: bisphenol AF
BPS: bisphenol S
BPZ: bisphenol Z
BPM: bisphenol M
NAC: N-acetylcysteine
GSH: glutathione
RMs: reactive metabolites
DFT: density functional theory
EA: electron affinity
*f* ^+^: Fukui function index
SOD: superoxide dismutases
CAT: catalase
GST: glutathione S-transferases
ROS: reactive oxygen species
MDA: lipid peroxidation
