# Supplementary material for: Covalently Active Metabolites of Bisphenol A Analogs by Mass Spectrometry Diagnostic Ions: Possible Mechanisms of Their Toxicity
Source: Chem Res Toxicol. 2026 Jan 6;39(1):157–67. doi: 10.1021/acs.chemrestox.5c00417 (PMC12820979; doi:10.1021/acs.chemrestox.5c00417)
Supplement: Supplementary file 1 [file tx5c00417_si_001.pdf]

Supporting Information

Covalently active metabolites of bisphenol A analogs by mass spectrometry diagnostic ions: possible mechanisms of their toxicity

Quan He[a], Xiaolan Hu[a,b,*], Xue Li[b], Na Li[a], Jian-Lin Wu[a,*]

[a]Faculty of Chinese Medicine & State Key Laboratory of Quality Research in Chinese Medicine, Macau University of Science and Technology, Avenida Wai Long, Taipa, Macau SAR, 999078, China

[b]College of Environment and Climate, Institute of Mass Spectrometry and Atmospheric Environment, Guangdong Provincial Key Laboratory of Speed Capability Research, Jinan University, Guangzhou, 510632, China

* Corresponding authors: Xiaolan Hu, Email: xiaolanhu@jnu.edu.cn. Jian-Lin Wu, Email: jlwu@must.edu.mo.

Summary

Table S1. Bisphenol A analogues covalently binds with NAC after bioactivation.

Figure S1. MS/MS spectrum and possible structure of BPB RM-NAC adducts in negative mode. (A) BC1, (B) BC2, (C) BC3, (D) BC4.

Figure S2. MS/MS spectrum and possible structure of BPC RM-NAC adducts in negative mode. (A) CC1, (B) CC2, (C) CC3.

Figure S3. MS/MS spectrum and possible structure of BPE RM-NAC adducts in negative mode. (A) EC1, (B) EC2, (C) EC3.

Figure S4. MS/MS spectrum and possible structure of BPF RM-NAC adducts in negative mode. (A) FC1, (B) FC2, (C) FC3, (D) FC4, (E) FC5.

Figure S5. MS/MS spectrum and possible structure of BPAF RM-NAC adducts, AFC1, in negative mode.

Figure S6. MS/MS spectrum and possible structure of BPS RM-NAC adducts in negative mode. (A) SC1, (B) SC2, (C) SC3.

Figure S7. MS/MS spectrum and possible structure of BPZ RM-NAC adducts in negative mode. (A) ZC1, (B) ZC2 and ZC3, (C) ZC4.

Figure S8. MS/MS spectrum and possible structure of BPM RM-NAC adducts in negative mode. (A) MC1, (B) MC2, (C) MC3.

Figure S9. MS/MS spectrum and possible structure of BPB RM-GSH adducts in positive mode. (A) BG1, (B) BG2, (C) BG3, (D) BG4, (E) BG5, (F) BG6, (G) BG7.

Figure S10. MS/MS spectrum and possible structure of BPC RM-GSH adducts in

positive mode. (A) CG1 and CG2, (B) CG3, (C) CG4, (D) CG5, (E) CG6.

Figure S11. MS/MS spectrum and possible structure of BPE RM-GSH adducts in positive mode. (A) EG1 and EG2, (B) EG3, (C) EG4, (D) EG5, (E) EG6, (F) EG7 and EG8.

Figure S12. MS/MS spectrum and possible structure of BPF RM-GSH adducts in positive mode. (A) FG1 and FG2, (B) FG3 and FG4, (C) FG5, (D) FG6, (E) FG7, (F) FG8, (G) FG9.

Figure S13. MS/MS spectrum and possible structure of BPAF RM-GSH adducts in positive mode. (A) AFG1, (B) AFG2, (C) AFG3.

Figure S14. MS/MS spectrum and possible structure of BPS RM-GSH adducts in positive mode. (A) SG1, (B) SG2.

Figure S15. MS/MS spectrum and possible structure of BPZ RM-GSH adducts in positive mode. (A) ZG1, (B) ZG2-4, (C) ZG5, (D) ZG6, (E) ZG7-8.

Figure S16. MS/MS spectrum and possible structure of BPM RM-GSH adducts in positive mode. (A) MG1, (B) MG2, (C) MG3.

Figure S17. Extracted ion chromatograms of GSH conjugates formed with BPB and its metabolites in microsomes.

Figure S18. Extracted ion chromatograms (A), possible structure (B), and time-course changes (C) of GSH conjugates formed with BPC and its metabolites in microsomes.

Figure S19. Extracted ion chromatograms (A), possible structure (B), and time-course changes (C) of GSH conjugates formed with BPE and its metabolites in microsomes.

Figure S20. Extracted ion chromatograms (A), possible structure (B), and time-course

changes (C) of GSH conjugates formed with BPF and its metabolites in microsomes.

Figure S21. Extracted ion chromatograms (A), possible structure (B), and time-course changes (C) of GSH conjugates formed with BPAF and its metabolites in microsomes.

Figure S22. Extracted ion chromatograms (A), possible structure (B), and time-course changes (C) of GSH conjugates formed with BPS and its metabolites in microsomes.

Figure S23. Extracted ion chromatograms (A), possible structure (B), and time-course changes (C) of GSH conjugates formed with BPZ and its metabolites in microsomes.

Figure S24. Extracted ion chromatograms (A), possible structure (B), and time-course changes (C) of GSH conjugates formed with BPM and its metabolites in microsomes.

Table S1. Bisphenol A analogues covalently binds with NAC after bioactivation.

NO.	BPs analogues adducts	Retention time (min)	Molecular Formula	Calculated *m/z* [M-H]$^-$	Measured *m/z* [M-H]$^-$	Mass accuracy (ppm)
1	BC1	7.9	$C_{21}H_{25}NO_5S$	402.1381	402.1385	-0.99
2	BC2	7.4	$C_{21}H_{25}NO_6S$	418.1330	418.1318	2.87
3	BC3	7.1	$C_{21}H_{25}NO_7S$	434.1279	434.1289	-2.30
4	BC4	5.2	$C_{11}H_{13}NO_4S$	254.0493	254.0491	0.79
5	CC1	8.4	$C_{22}H_{27}NO_5S$	416.1537	416.1547	-2.40
6	CC2	8.3	$C_{22}H_{27}NO_6S$	432.1486	432.1497	-2.55
7	CC3	6.1	$C_{12}H_{15}NO_4S$	268.0649	268.0664	-5.60
8	EC1	7.2	$C_{19}H_{21}NO_5S$	374.1068	374.1075	-1.87
9	EC2	6.7	$C_{19}H_{21}NO_6S$	390.1017	390.1024	-1.79
10	EC3	5.2	$C_{11}H_{13}NO_4S$	254.0493	254.0495	-0.79
11	FC1	7.9	$C_{18}H_{19}NO_5S$	360.0911	360.0916	-1.39
12	FC2	7.3	$C_{18}H_{19}NO_6S$	376.0860	376.0864	-1.06
13	FC3	6.9	$C_{18}H_{19}NO_7S$	392.0809	392.0807	0.51
14	FC4	6.1	$C_{11}H_{13}NO_4S$	254.0493	254.0489	1.57
15	FC5	3.8	$C_{11}H_{13}NO_5S$	270.0442	270.0439	1.11
16	AFC1	7.9	$C_{20}H_{17}FNO_6S$	512.0608	512.0605	0.59
17	SC1	6.6	$C_{17}H_{17}NO_8S_2$	426.0323	426.0319	0.94
18	SC2	6.2	$C_{17}H_{17}NO_9S_2$	442.0272	442.0271	0.23
19	SC3	3.5	$C_{11}H_{13}NO_5S$	270.0442	270.0451	-3.33
20	ZC1	8.3	$C_{23}H_{27}NO_5S$	428.1537	428.1538	-0.23
21	ZC2	7.8	$C_{23}H_{27}NO_6S$	444.1486	444.1486	0.00
22	ZC3	6.2	$C_{23}H_{27}NO_6S$	444.1486	444.1491	-1.13
23	ZC4	5.2	$C_{11}H_{13}NO_4S$	254.0493	254.0504	-4.33
24	MC1	9.3	$C_{29}H_{33}NO_5S$	506.2007	506.2015	-1.58
25	MC2	8.8	$C_{29}H_{33}NO_6S$	522.1956	522.1961	-0.96
26	MC3	8.1	$C_{29}H_{33}NO_7S$	538.1905	538.1881	4.46

A

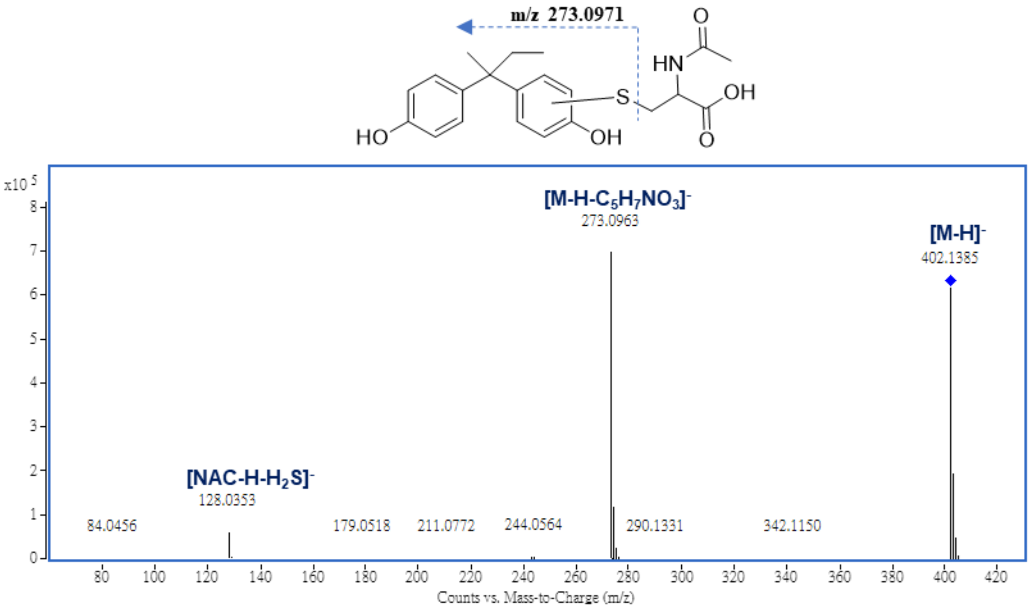

m/z 273.0971
[M-H-C5H7NO3]-
273.0963
[M-H]-
402.1385
[NAC-H-H2S]-
128.0353
84.0456
179.0518
211.0772
244.0564
290.1331
342.1150
Counts vs. Mass-to-Charge (m/z)

B

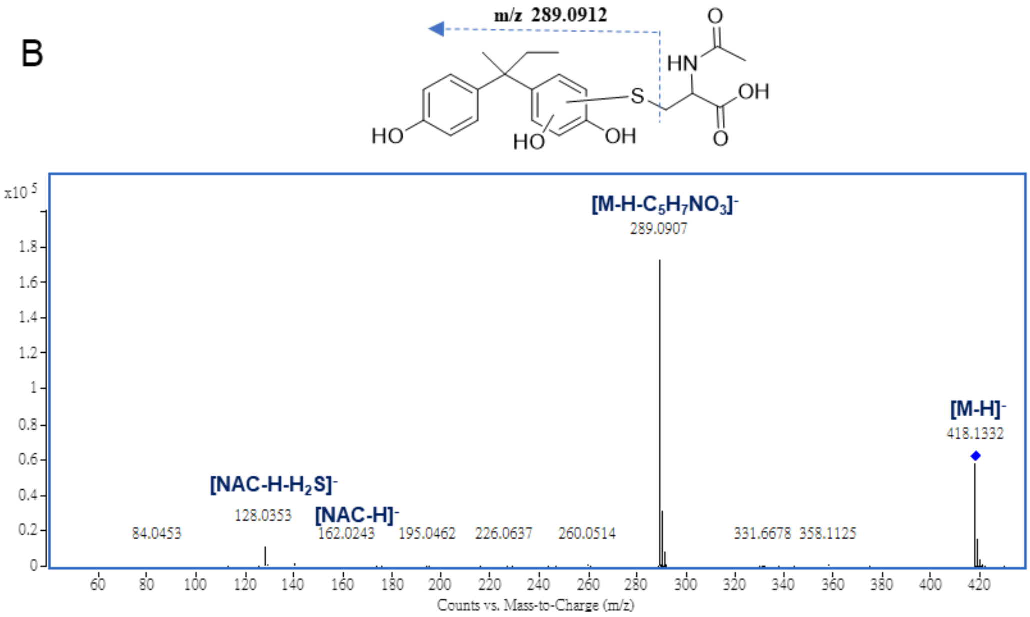

m/z 289.0912
[M-H-C5H7NO3]-
289.0907
[M-H]-
418.1332
[NAC-H-H2S]-
128.0353
[NAC-H]-
162.0243
84.0453
195.0462
226.0637
260.0514
331.6678
358.1125
Counts vs. Mass-to-Charge (m/z)

C

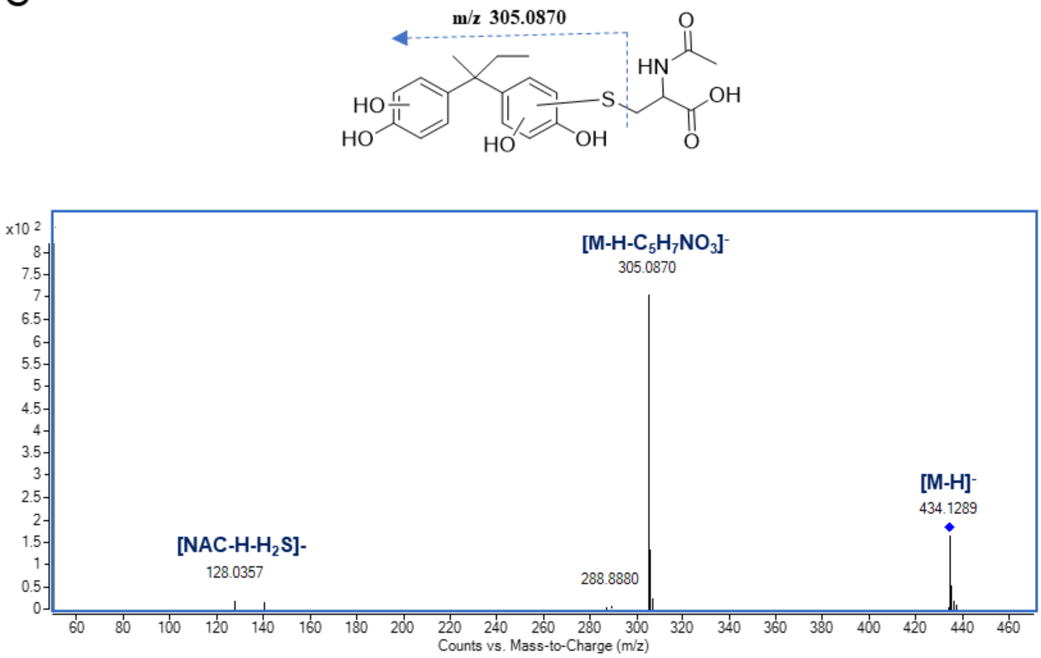

D

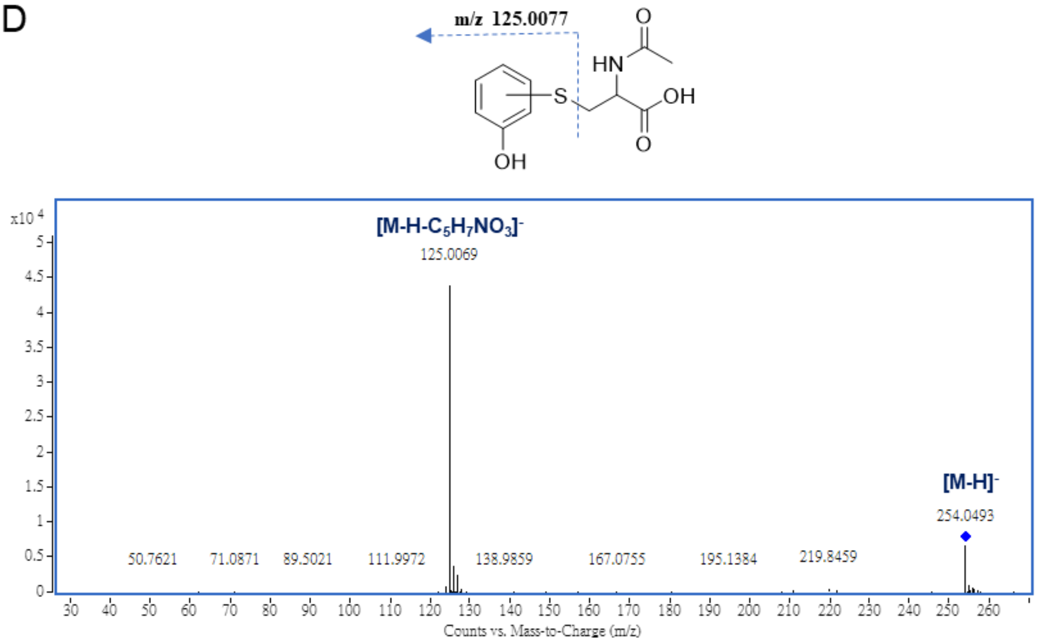

Figure S1. MS/MS spectrum and possible structure of BPB RM-NAC adducts in negative mode. (A) BC1, (B) BC2, (C) BC3, (D) BC4.

A

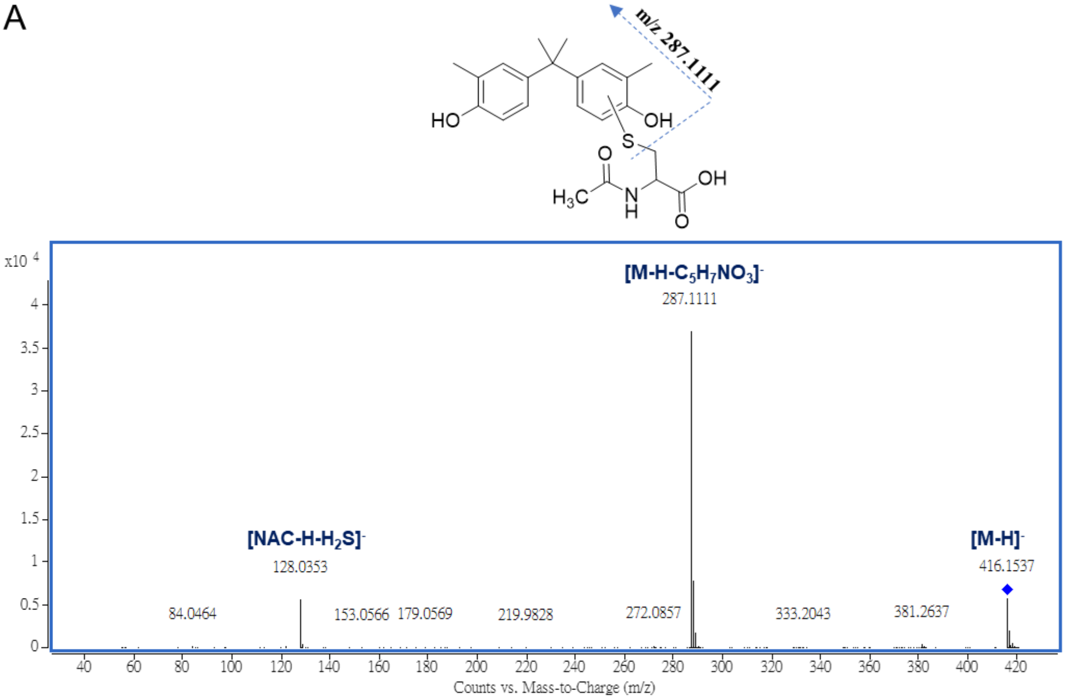
m/z 287.1111
HO
OH
S
O
H3C
N
H
OH
O
x10 4
[M-H-C5H7NO3]-
287.1111
[NAC-H-H2S]-
128.0353
[M-H]-
416.1537
84.0464
153.0566
179.0569
219.9828
272.0857
333.2043
381.2637
Counts vs. Mass-to-Charge (m/z)

B

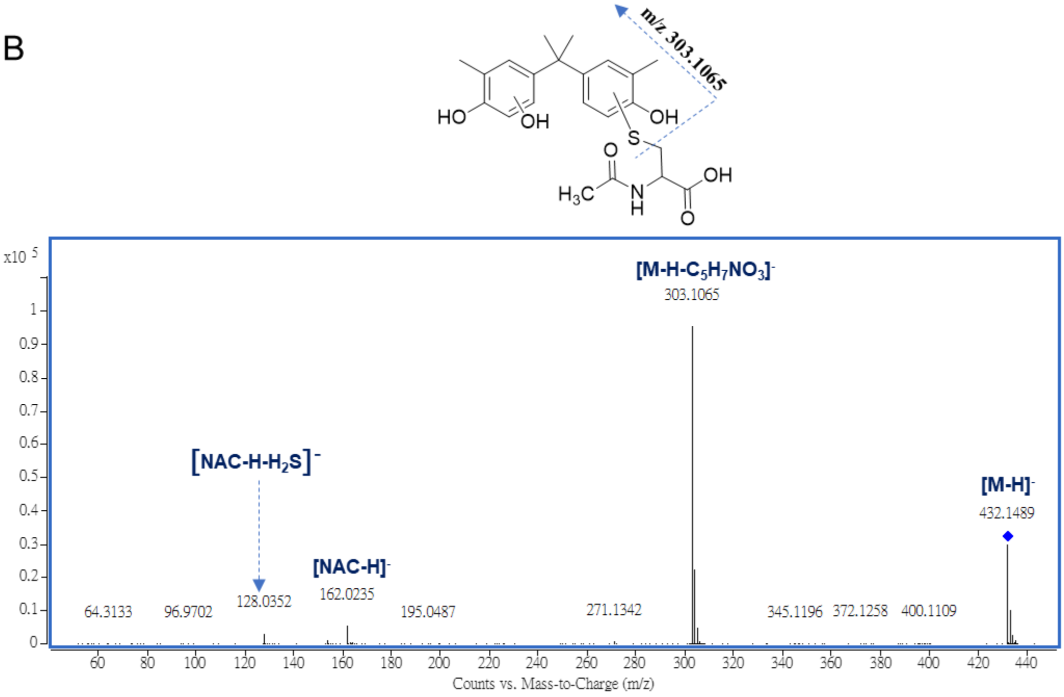
m/z 303.1065
HO
OH
OH
S
O
H3C
N
H
OH
O
x10 5
[M-H-C5H7NO3]-
303.1065
[NAC-H-H2S]-
128.0352
[NAC-H]-
162.0235
[M-H]-
432.1489
64.3133
96.9702
195.0487
271.1342
345.1196
372.1258
400.1109
Counts vs. Mass-to-Charge (m/z)

C

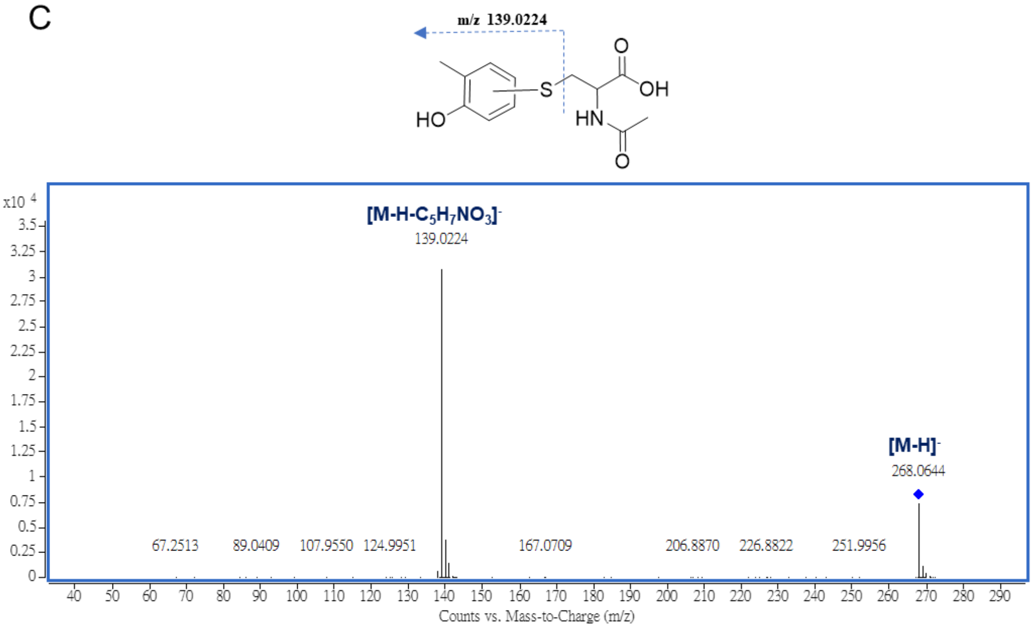

Figure S2. MS/MS spectrum and possible structure of BPC RM-NAC adducts in negative mode. (A) CC1, (B) CC2, (C) CC3.

A

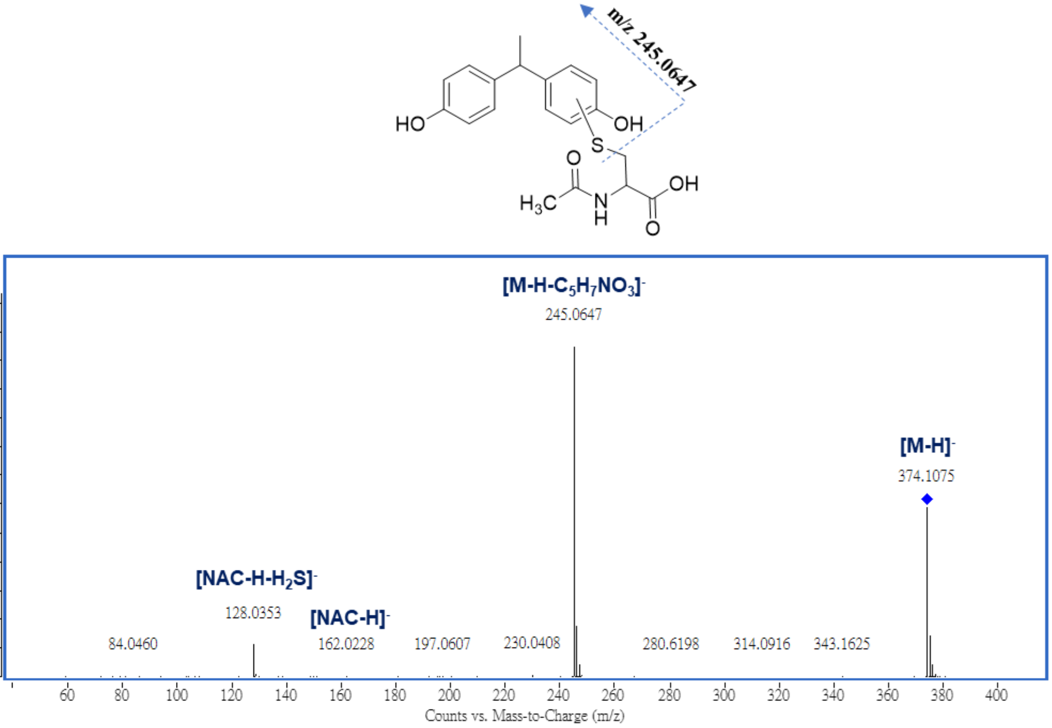

m/z 245.0647
HO
OH
S
O
OH
H_3C
N
H
O
x10 5
Counts vs. Mass-to-Charge (m/z)
$[M-H-C_5H_7NO_3]^-$
245.0647
$[M-H]^-$
374.1075
$[NAC-H-H_2S]^-$
128.0353
$[NAC-H]^-$
84.0460
162.0228
197.0607
230.0408
280.6198
314.0916
343.1625

B

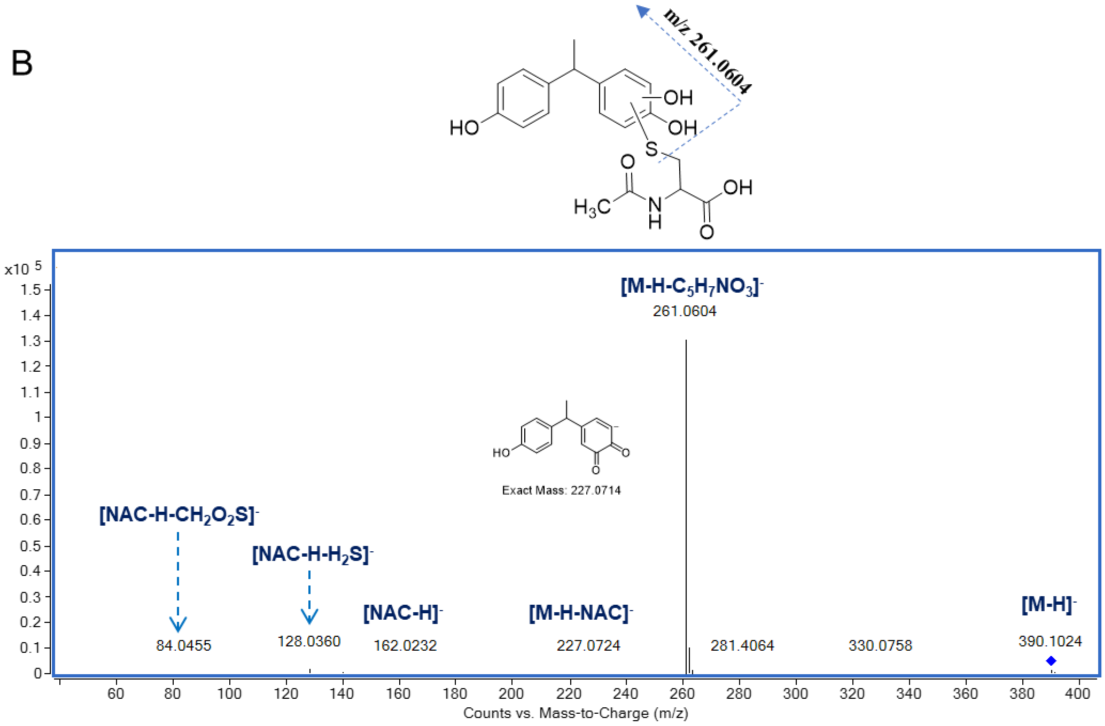

m/z 261.0604
HO
OH
OH
S
O
OH
H_3C
N
H
O
x10 5
Counts vs. Mass-to-Charge (m/z)
$[M-H-C_5H_7NO_3]^-$
261.0604
HO
O
O
Exact Mass: 227.0714
$[NAC-H-CH_2O_2S]^-$
$[NAC-H-H_2S]^-$
$[NAC-H]^-$
$[M-H-NAC]^-$
$[M-H]^-$
84.0455
128.0360
162.0232
227.0724
281.4064
330.0758
390.1024

C

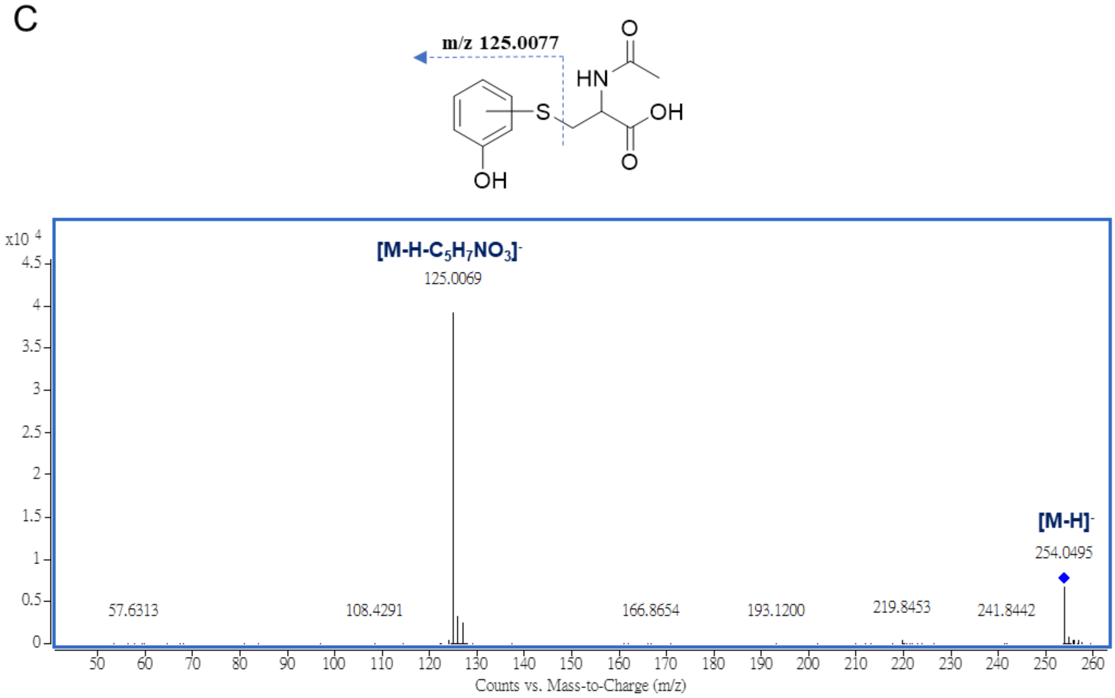

Figure S3. MS/MS spectrum and possible structure of BPE RM-NAC adducts in negative mode. (A) EC1, (B) EC2, (C) EC3.

A

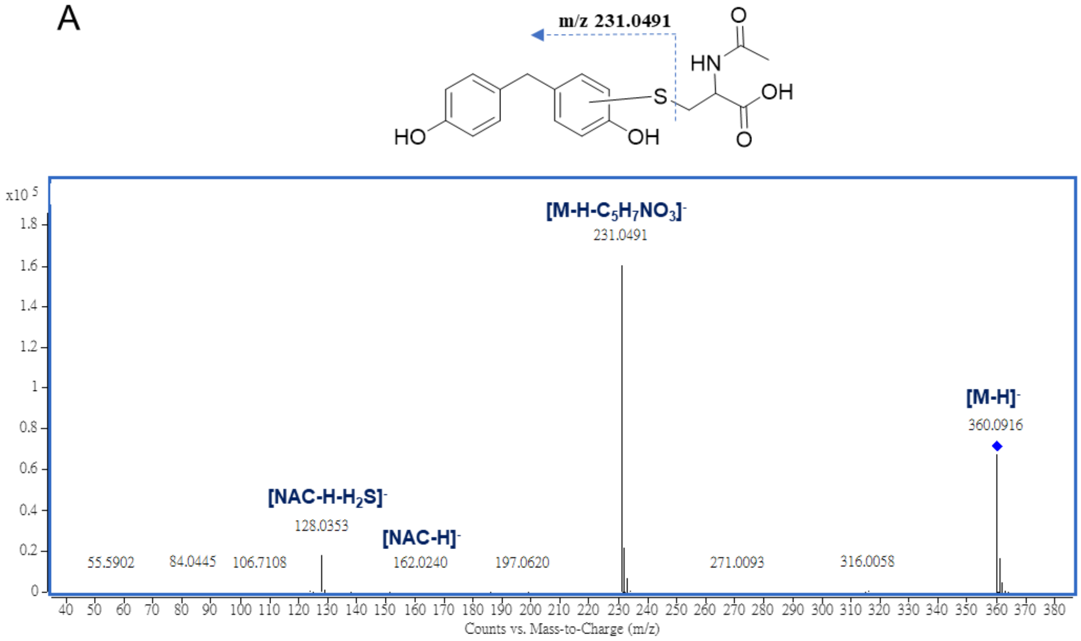

m/z 231.0491
HN
OH
HO
S
O
[M-H-C5H7NO3]-
231.0491
[M-H]-
360.0916
[NAC-H-H2S]-
128.0353
[NAC-H]-
162.0240
55.5902
84.0445
106.7108
197.0620
271.0093
316.0058
x10 5
Counts vs. Mass-to-Charge (m/z)

B

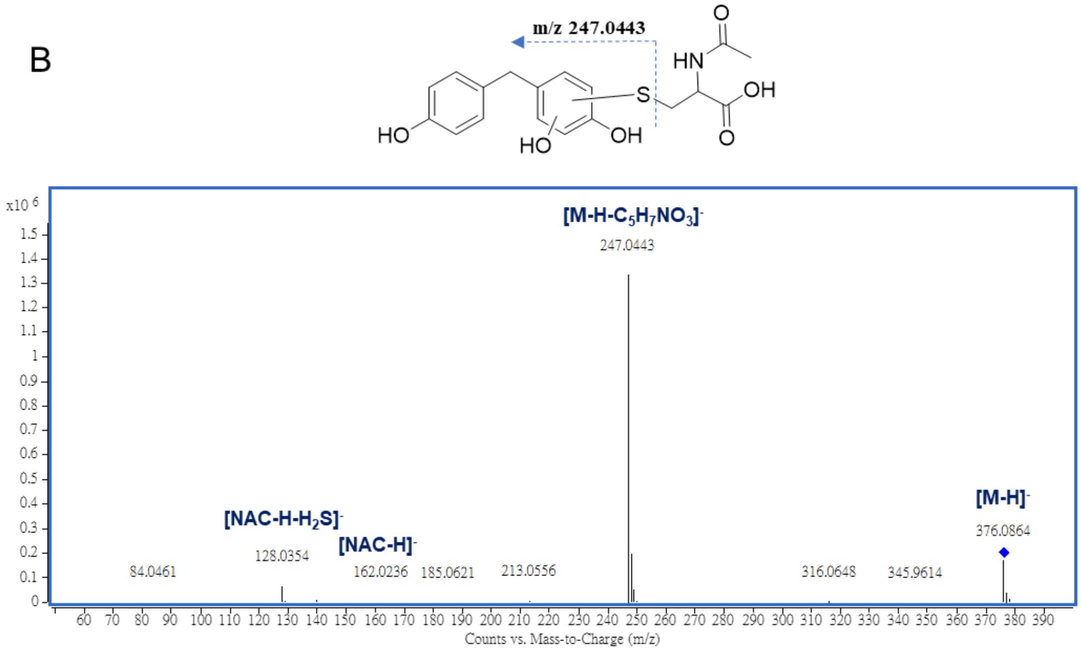

m/z 247.0443
HN
OH
HO
S
O
[M-H-C5H7NO3]-
247.0443
[M-H]-
376.0864
[NAC-H-H2S]-
128.0354
[NAC-H]-
162.0236
84.0461
185.0621
213.0556
316.0648
345.9614
x10 6
Counts vs. Mass-to-Charge (m/z)

C

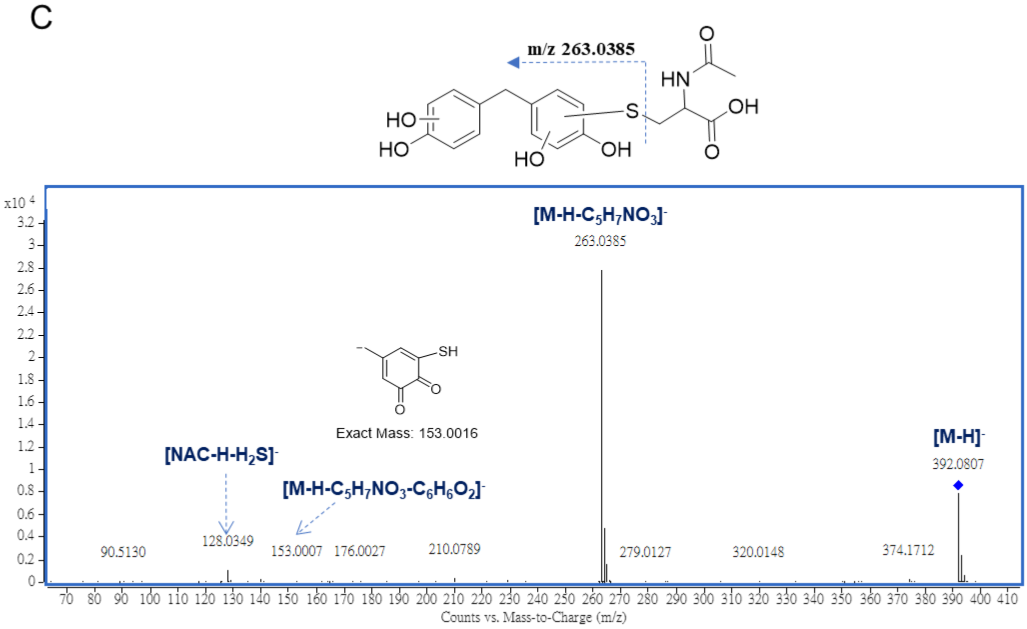

m/z 263.0385
HO
HO
HO
OH
S
HN
O
OH
O
x10 4
[M-H-C5H7NO3]-
263.0385
SH
O
O
Exact Mass: 153.0016
[NAC-H-H2S]-
[M-H-C5H7NO3-C6H6O2]-
[M-H]-
392.0807
90.5130
128.0349
153.0007
176.0027
210.0789
279.0127
320.0148
374.1712
Counts vs. Mass-to-Charge (m/z)

D

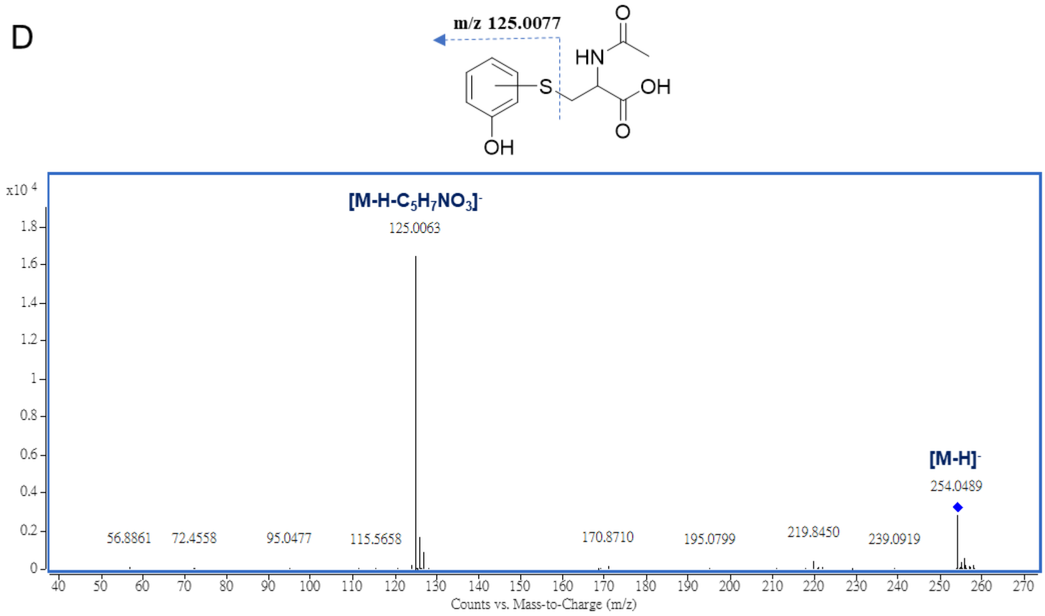

m/z 125.0077
O
HN
S
OH
O
OH
x10 4
[M-H-C5H7NO3]-
125.0063
[M-H]-
254.0489
56.8861
72.4558
95.0477
115.5658
170.8710
195.0799
219.8450
239.0919
Counts vs. Mass-to-Charge (m/z)

E

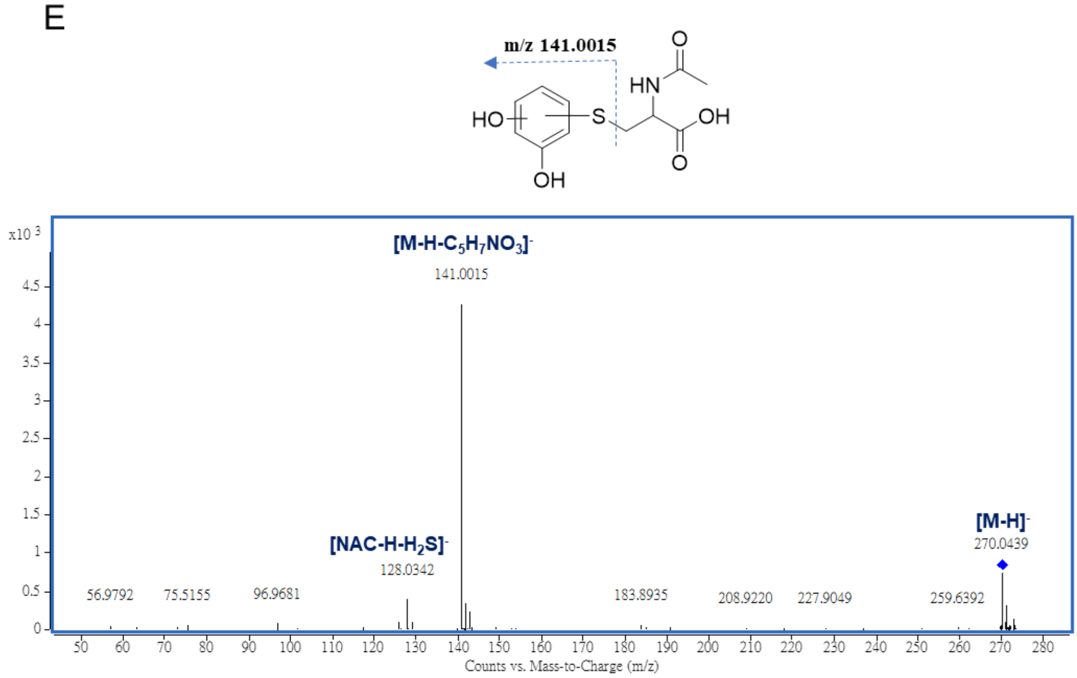

Figure S4. MS/MS spectrum and possible structure of BPF RM-NAC adducts in negative mode. (A) FC1, (B) FC2, (C) FC3, (D) FC4, (E) FC5.

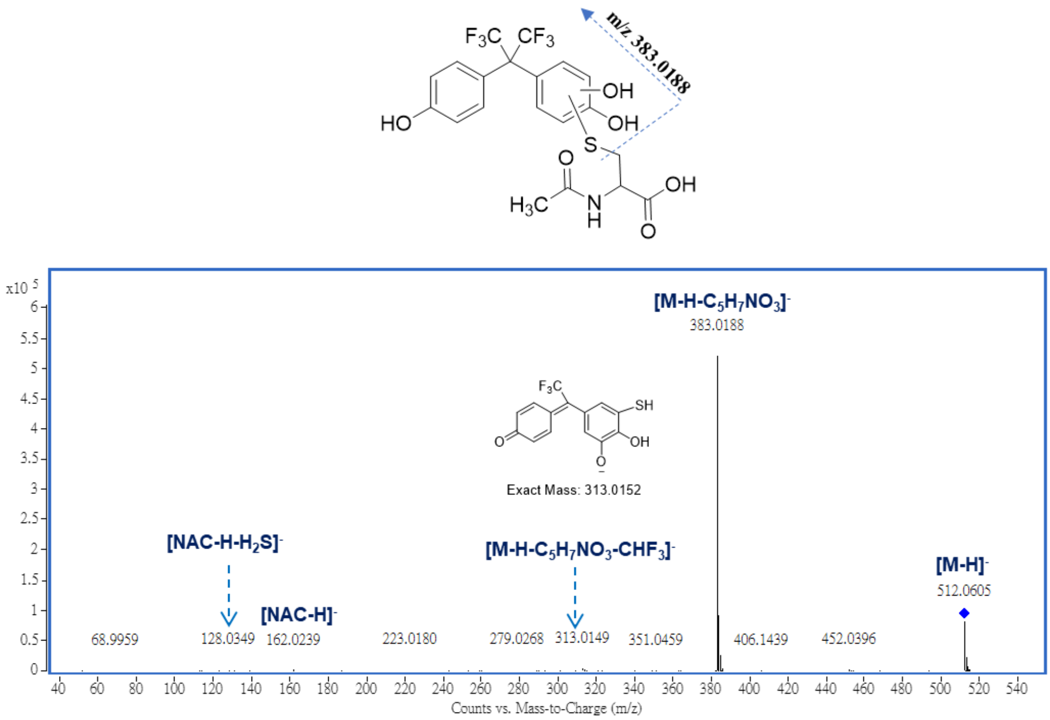

Figure S5. MS/MS spectrum and possible structure of BPAF RM-NAC adducts, AFC1, in negative mode.

A

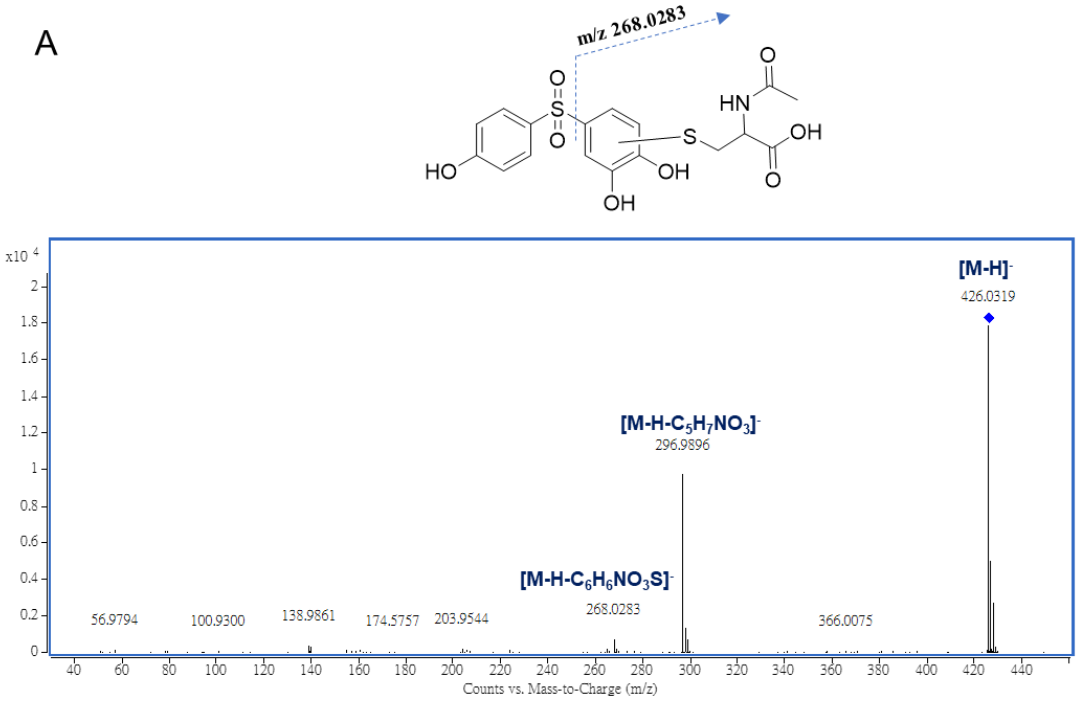

m/z 268.0283
[M-H]-
426.0319
[M-H-C5H7NO3]-
296.9896
[M-H-C6H6NO3S]-
268.0283
56.9794
100.9300
138.9861
174.5757
203.9544
366.0075
x10 4
Counts vs. Mass-to-Charge (m/z)

B

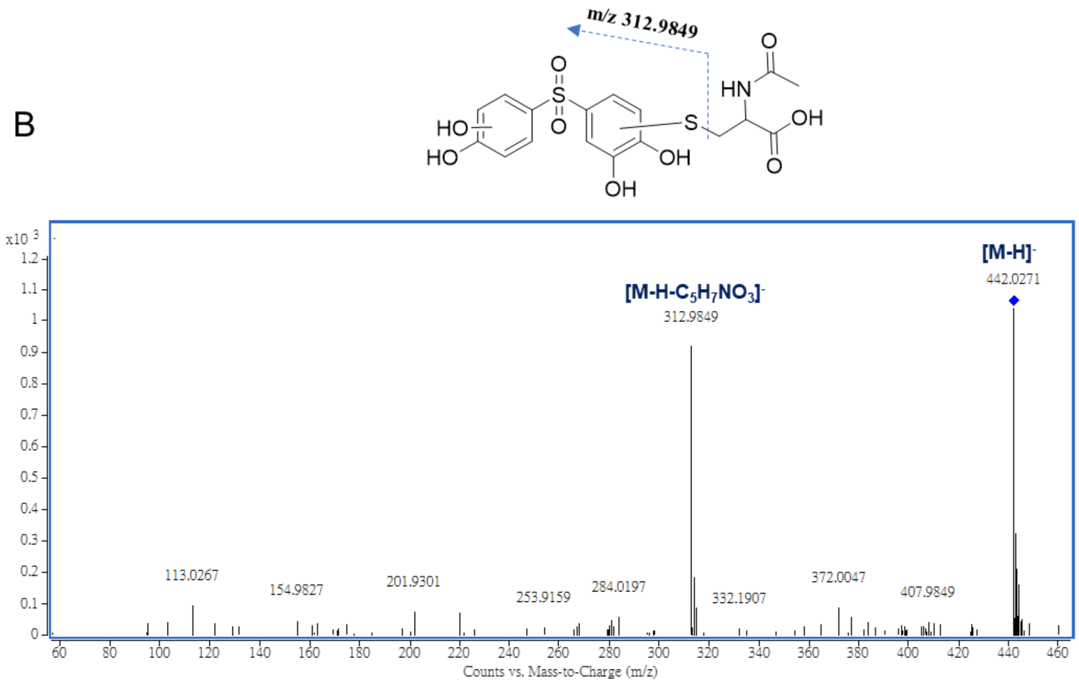

m/z 312.9849
[M-H]-
442.0271
[M-H-C5H7NO3]-
312.9849
113.0267
154.9827
201.9301
253.9159
284.0197
332.1907
372.0047
407.9849
x10 3
Counts vs. Mass-to-Charge (m/z)

C

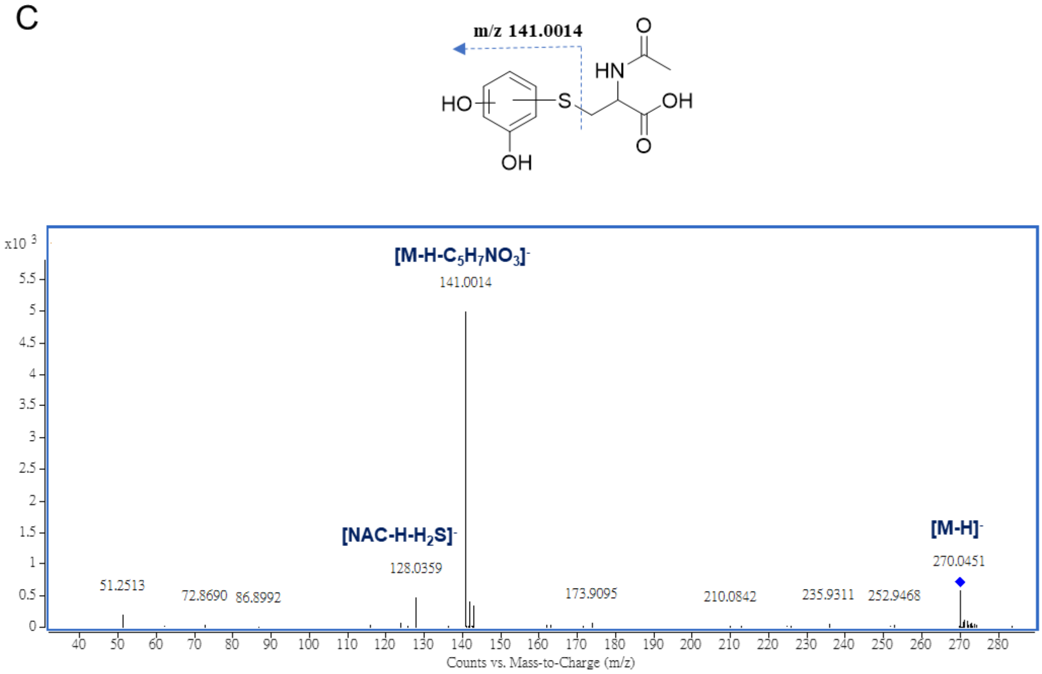

Figure S6. MS/MS spectrum and possible structure of BPS RM-NAC adducts in negative mode. (A) SC1, (B) SC2, (C) SC3.

A

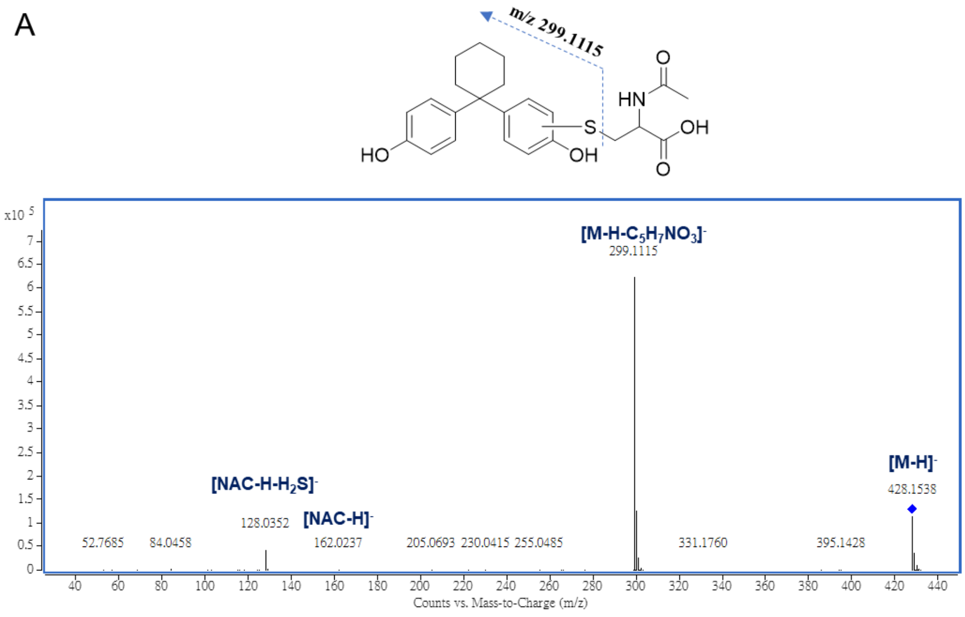

m/z 299.1115
[M-H-C5H7NO3]-
299.1115
[NAC-H-H2S]-
128.0352
[NAC-H]-
162.0237
[M-H]-
428.1538
52.7685
84.0458
205.0693 230.0415 255.0485
331.1760
395.1428
x10 5
Counts vs. Mass-to-Charge (m/z)

B

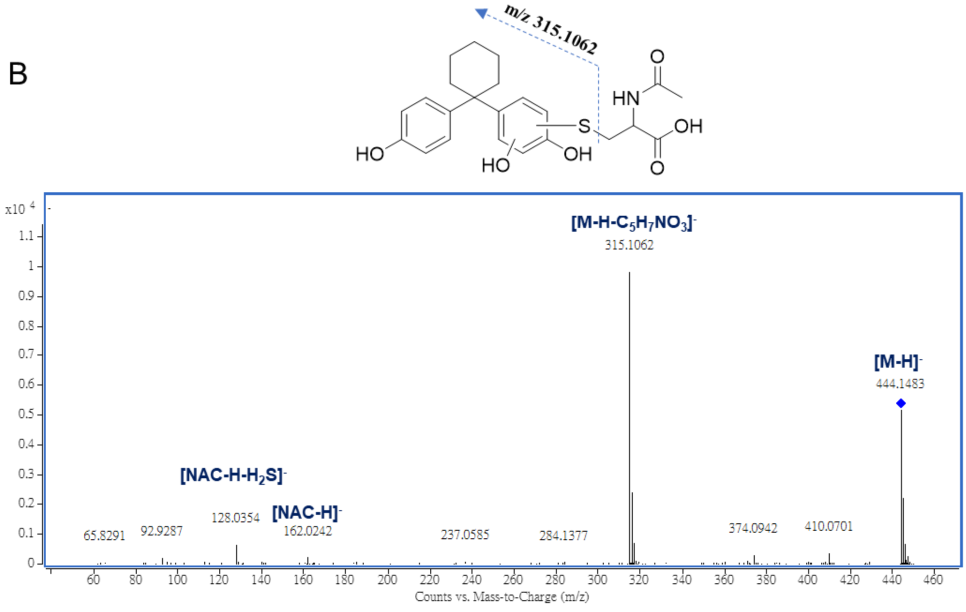

m/z 315.1062
[M-H-C5H7NO3]-
315.1062
[NAC-H-H2S]-
128.0354
[NAC-H]-
162.0242
[M-H]-
444.1483
65.8291
92.9287
237.0585
284.1377
374.0942
410.0701
x10 4
Counts vs. Mass-to-Charge (m/z)

C

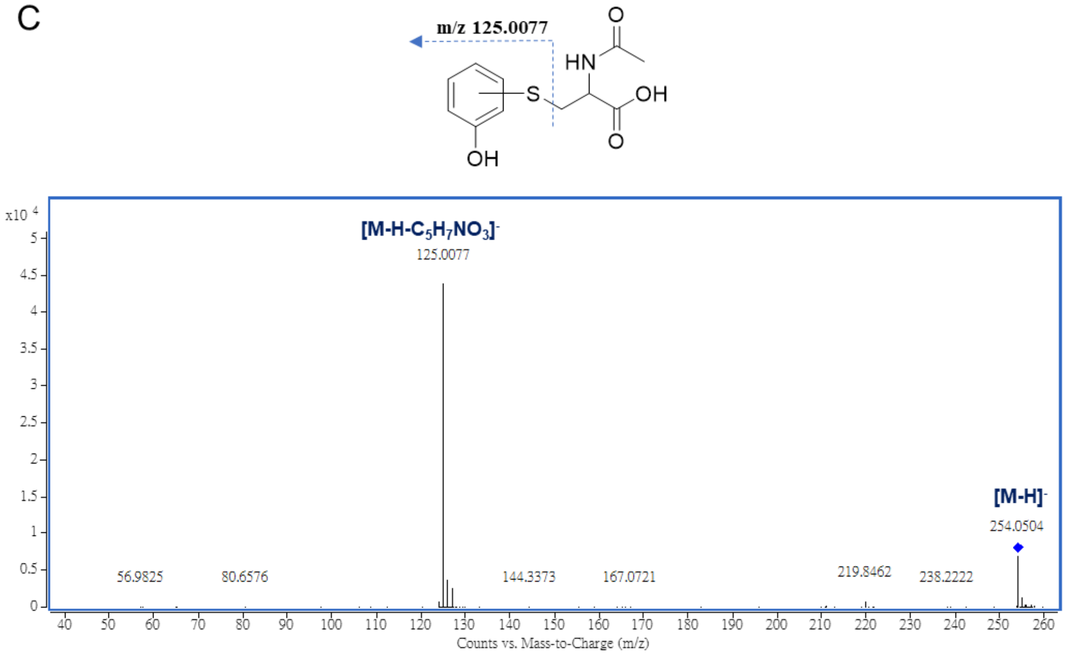

Figure S7. MS/MS spectrum and possible structure of BPZ RM-NAC adducts in negative mode. (A) ZC1, (B) ZC2 and ZC3, (C) ZC4.

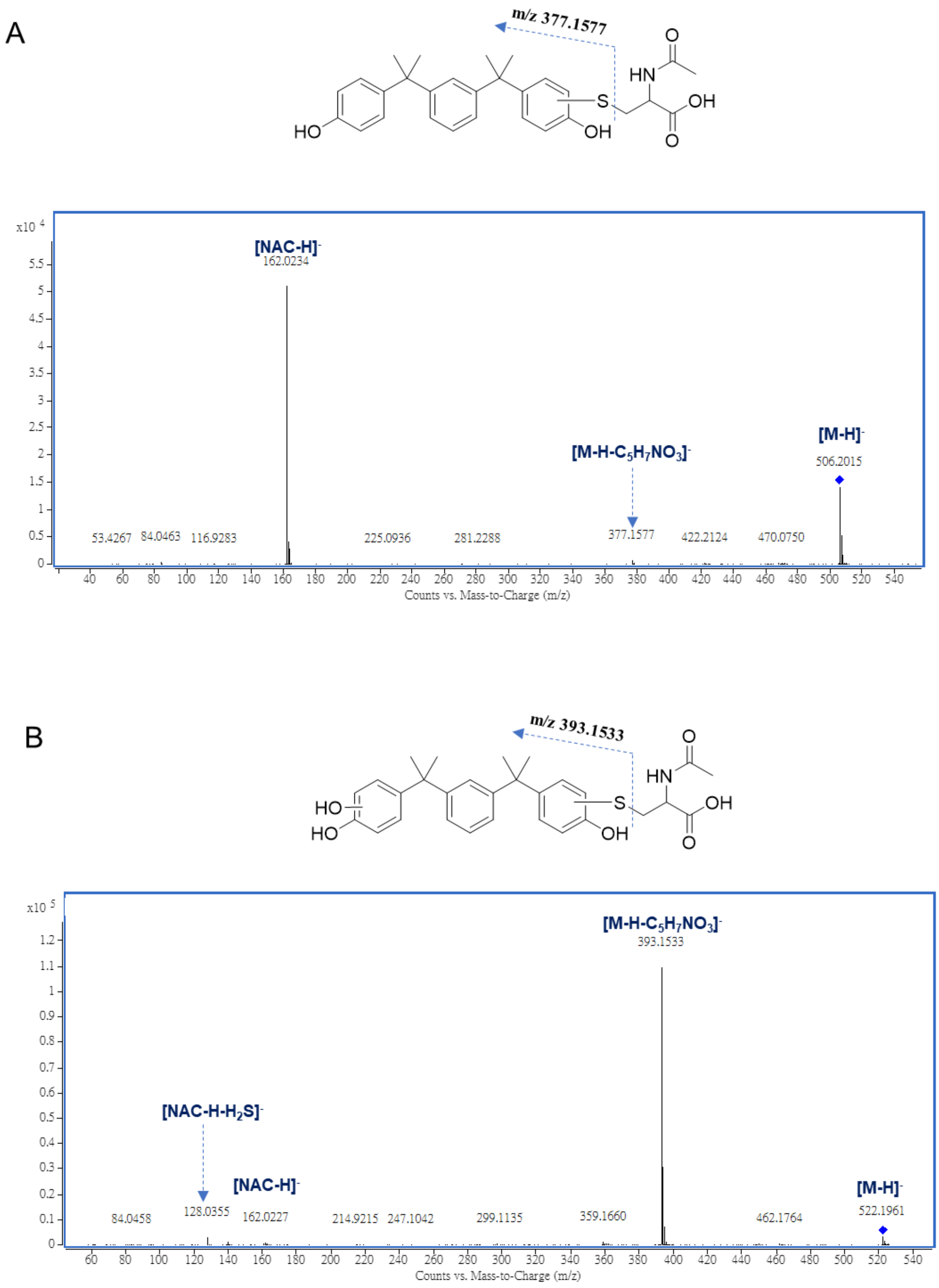

Figure S8. MS/MS spectrum and possible structure of BPM RM-NAC adducts in negative mode. (A) MC1, (B) MC2, (C) MC3.

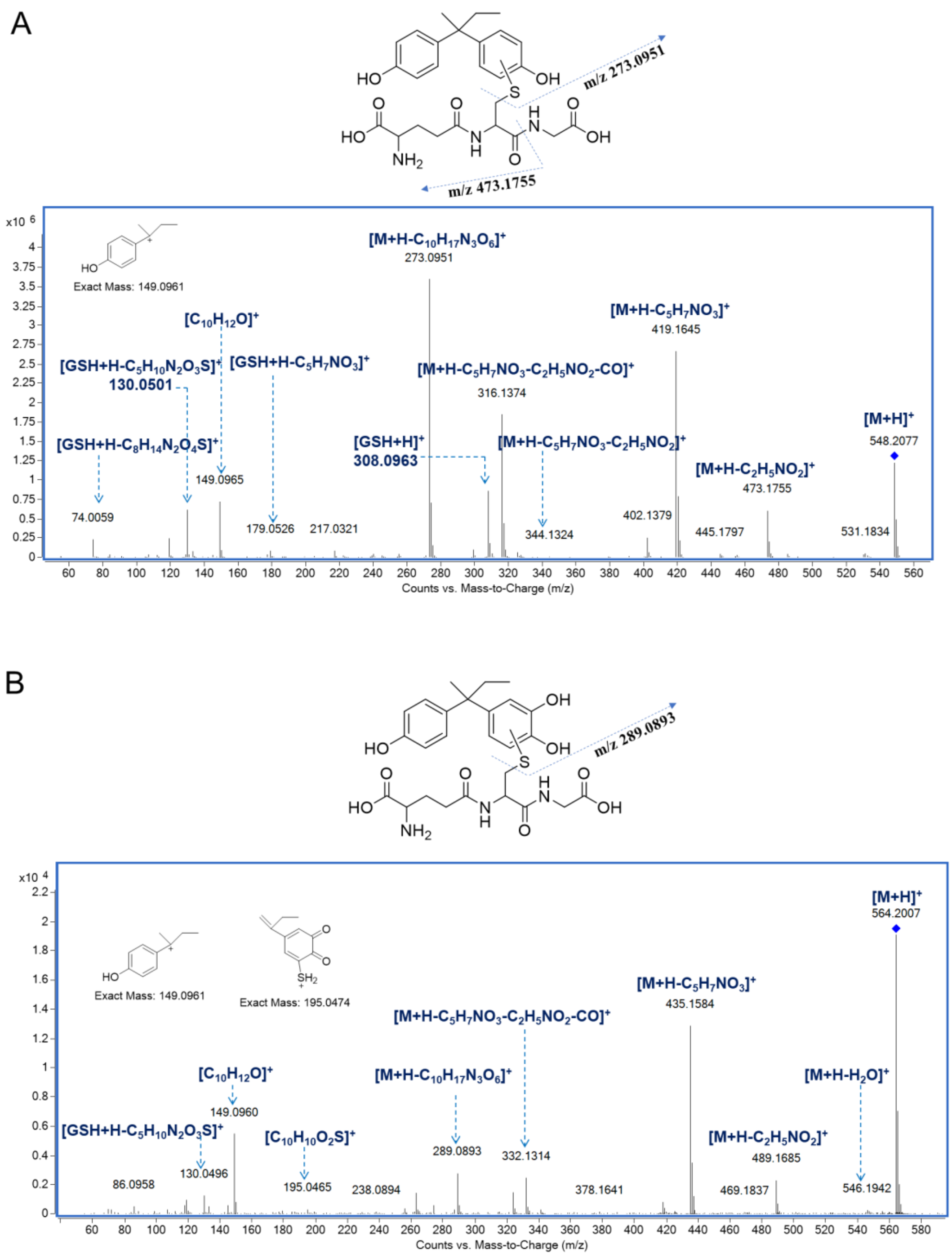

A
m/z 273.0951
m/z 473.1755
HO
OH
S
NH2
x10 6
Exact Mass: 149.0961
[M+H-C10H17N3O6]+
273.0951
[C10H12O]+
149.0965
[M+H-C5H7NO3]+
419.1645
[GSH+H-C5H10N2O3S]+
130.0501
[GSH+H-C5H7NO3]+
179.0526
[M+H-C5H7NO3-C2H5NO2-CO]+
316.1374
[M+H]+
548.2077
[GSH+H-C8H14N2O4S]+
74.0059
[GSH+H]+
308.0963
[M+H-C5H7NO3-C2H5NO2]+
344.1324
[M+H-C2H5NO2]+
473.1755
217.0321
402.1379
445.1797
531.1834
Counts vs. Mass-to-Charge (m/z)
B
m/z 289.0893
x10 4
Exact Mass: 149.0961
Exact Mass: 195.0474
[M+H]+
564.2007
[M+H-C5H7NO3]+
435.1584
[M+H-C5H7NO3-C2H5NO2-CO]+
332.1314
[C10H12O]+
149.0960
[M+H-C10H17N3O6]+
289.0893
[M+H-H2O]+
546.1942
[GSH+H-C5H10N2O3S]+
130.0496
[C10H10O2S]+
195.0465
[M+H-C2H5NO2]+
489.1685
86.0958
238.0894
378.1641
469.1837
Counts vs. Mass-to-Charge (m/z)

C

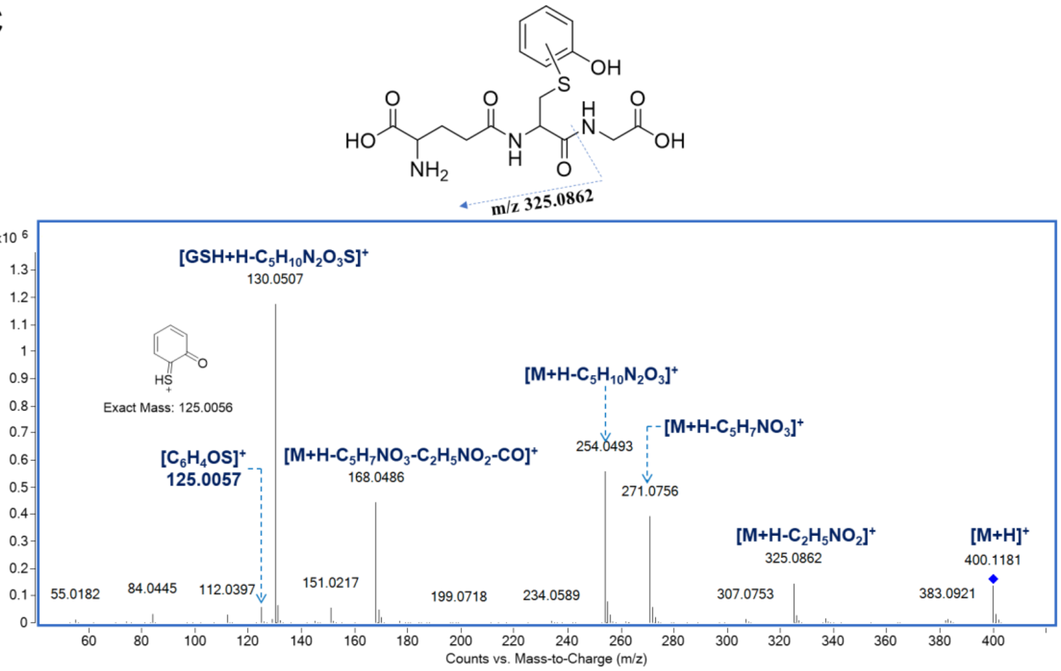
OH
S
O
O
H
N
O
HO
N
H
OH
NH2
O
m/z 325.0862
x10 6
[GSH+H-C5H10N2O3S]+
130.0507
HS
Exact Mass: 125.0056
[M+H-C5H10N2O3]+
254.0493
[M+H-C5H7NO3]+
271.0756
[C6H4OS]+
125.0057
[M+H-C5H7NO3-C2H5NO2-CO]+
168.0486
[M+H-C2H5NO2]+
325.0862
[M+H]+
400.1181
55.0182
84.0445
112.0397
151.0217
199.0718
234.0589
307.0753
383.0921
Counts vs. Mass-to-Charge (m/z)

D

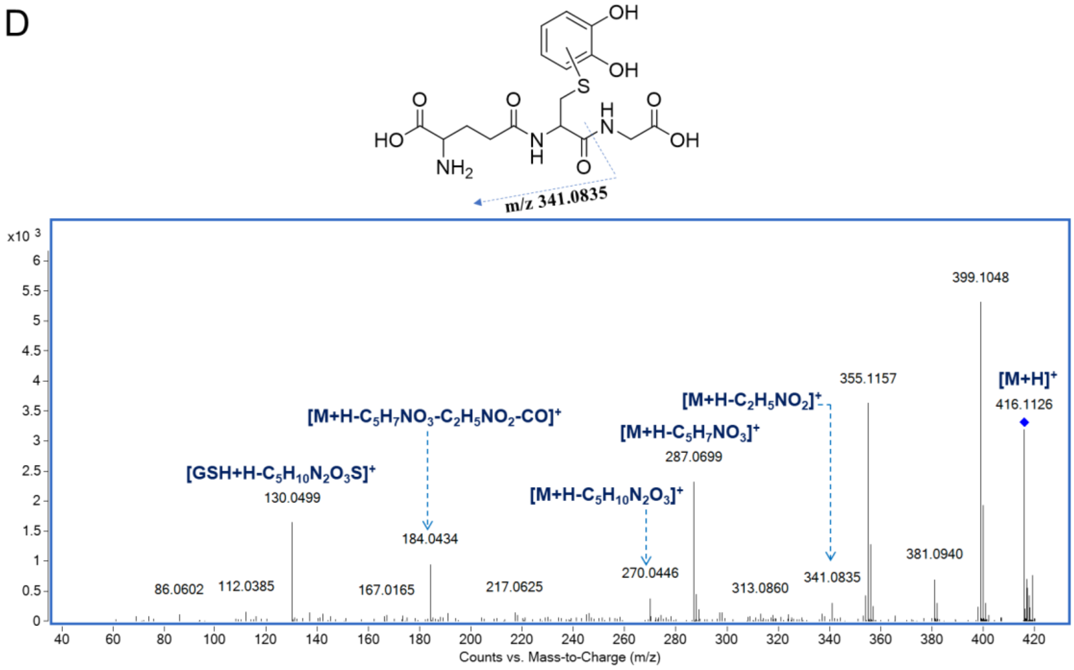
OH
OH
S
O
O
H
N
O
HO
N
H
OH
NH2
O
m/z 341.0835
x10 3
399.1048
355.1157
[M+H]+
416.1126
[M+H-C2H5NO2]+
[M+H-C5H7NO3-C2H5NO2-CO]+
[M+H-C5H7NO3]+
287.0699
[GSH+H-C5H10N2O3S]+
130.0499
[M+H-C5H10N2O3]+
184.0434
270.0446
381.0940
341.0835
86.0602
112.0385
167.0165
217.0625
313.0860
Counts vs. Mass-to-Charge (m/z)

E

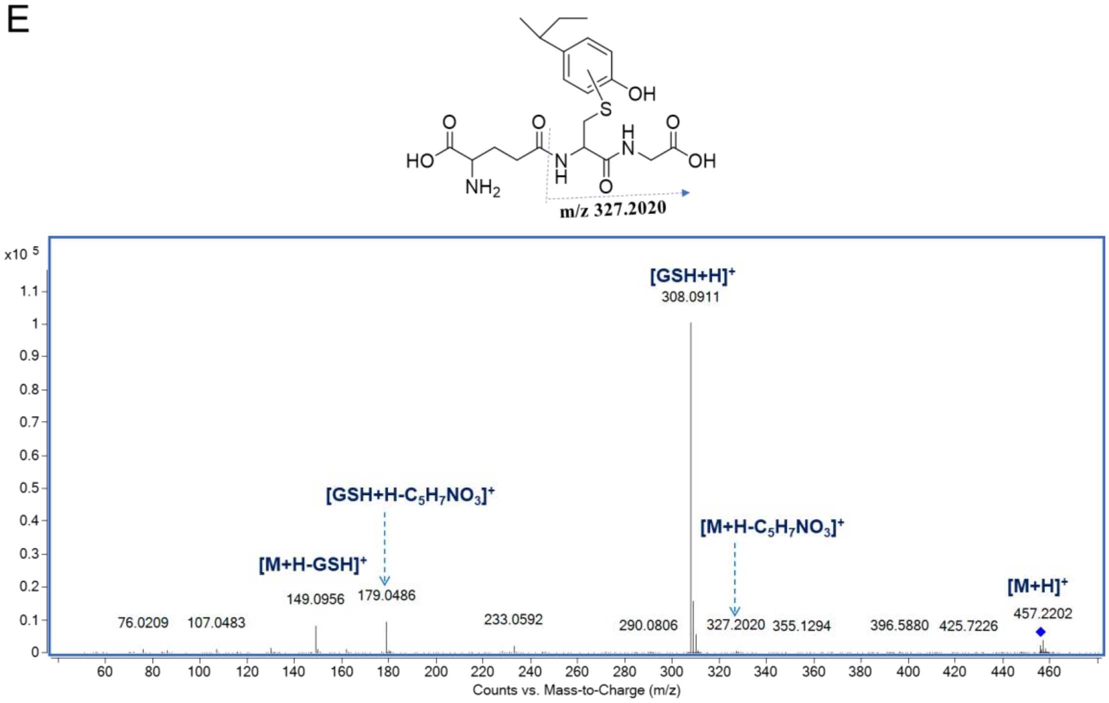
OH
S
HO
O
O
N
H
H
N
O
O
OH
NH2
m/z 327.2020
x10 5
[GSH+H]+
308.0911
[GSH+H-C5H7NO3]+
[M+H-C5H7NO3]+
[M+H-GSH]+
149.0956
179.0486
[M+H]+
76.0209
107.0483
233.0592
290.0806
327.2020
355.1294
396.5880
425.7226
457.2202
Counts vs. Mass-to-Charge (m/z)

F

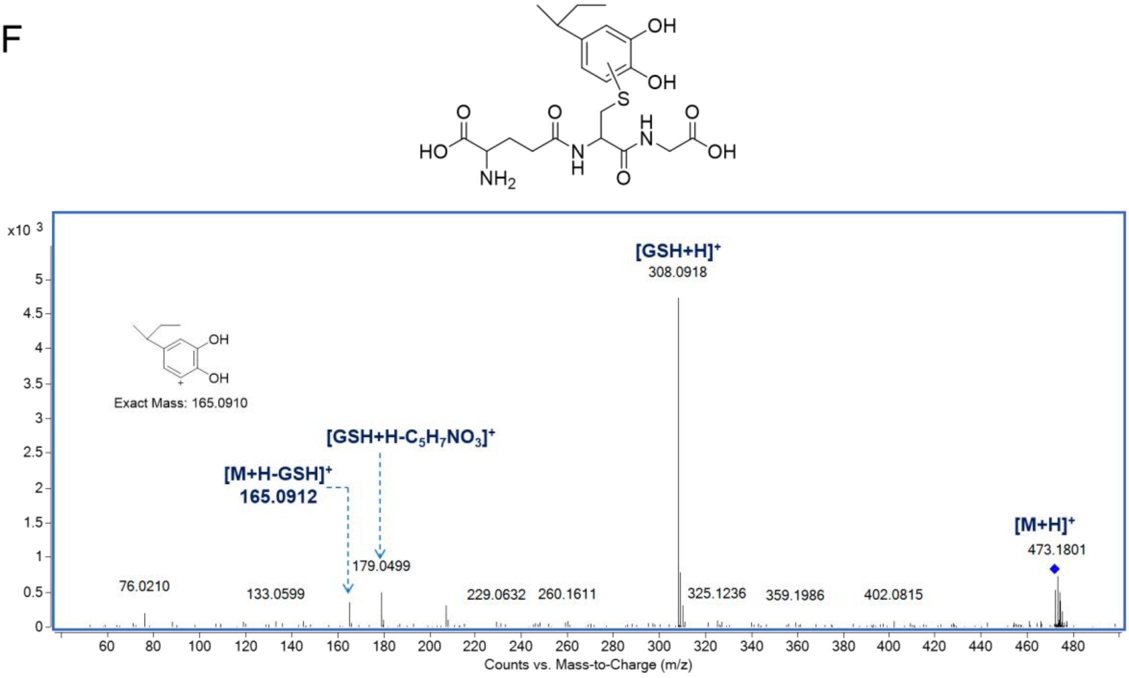
OH
OH
S
HO
O
O
N
H
H
N
O
O
OH
NH2
x10 3
[GSH+H]+
308.0918
OH
OH
Exact Mass: 165.0910
[GSH+H-C5H7NO3]+
[M+H-GSH]+
165.0912
[M+H]+
473.1801
179.0499
76.0210
133.0599
229.0632
260.1611
325.1236
359.1986
402.0815
Counts vs. Mass-to-Charge (m/z)

G

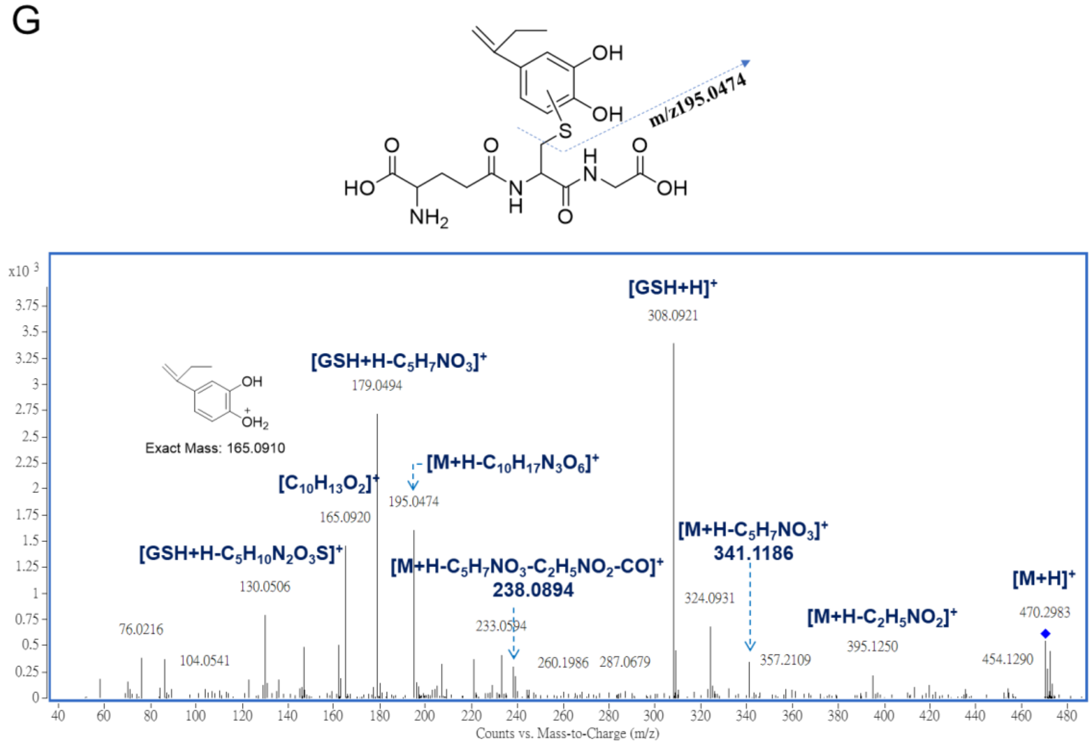

Figure S9. MS/MS spectrum and possible structure of BPB RM-GSH adducts in positive mode. (A) BG1, (B) BG2, (C) BG3, (D) BG4, (E) BG5, (F) BG6, (G) BG7.

A

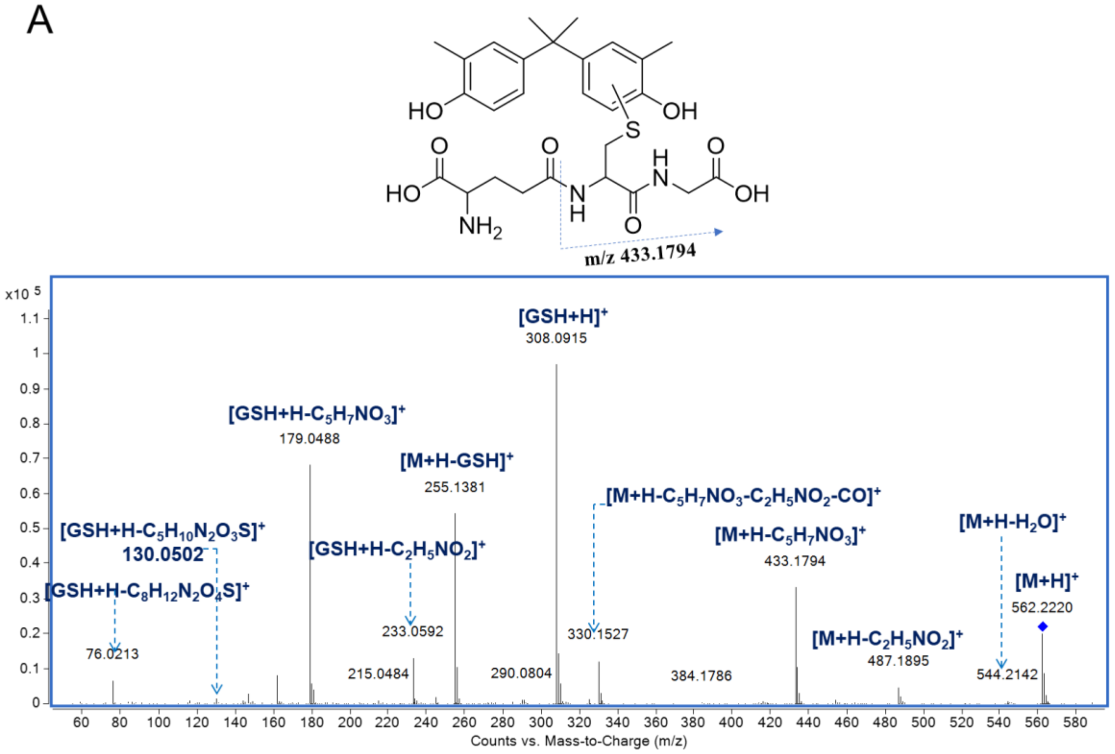
m/z 433.1794
x10 5
[GSH+H]+
308.0915
[GSH+H-C5H7NO3]+
179.0488
[M+H-GSH]+
255.1381
[M+H-C5H7NO3-C2H5NO2-CO]+
[GSH+H-C5H10N2O3S]+
130.0502
[GSH+H-C2H5NO2]+
[M+H-C5H7NO3]+
433.1794
[M+H-H2O]+
[GSH+H-C8H12N2O4S]+
[M+H]+
562.2220
76.0213
233.0592
330.1527
[M+H-C2H5NO2]+
487.1895
215.0484
290.0804
384.1786
544.2142
Counts vs. Mass-to-Charge (m/z)

B

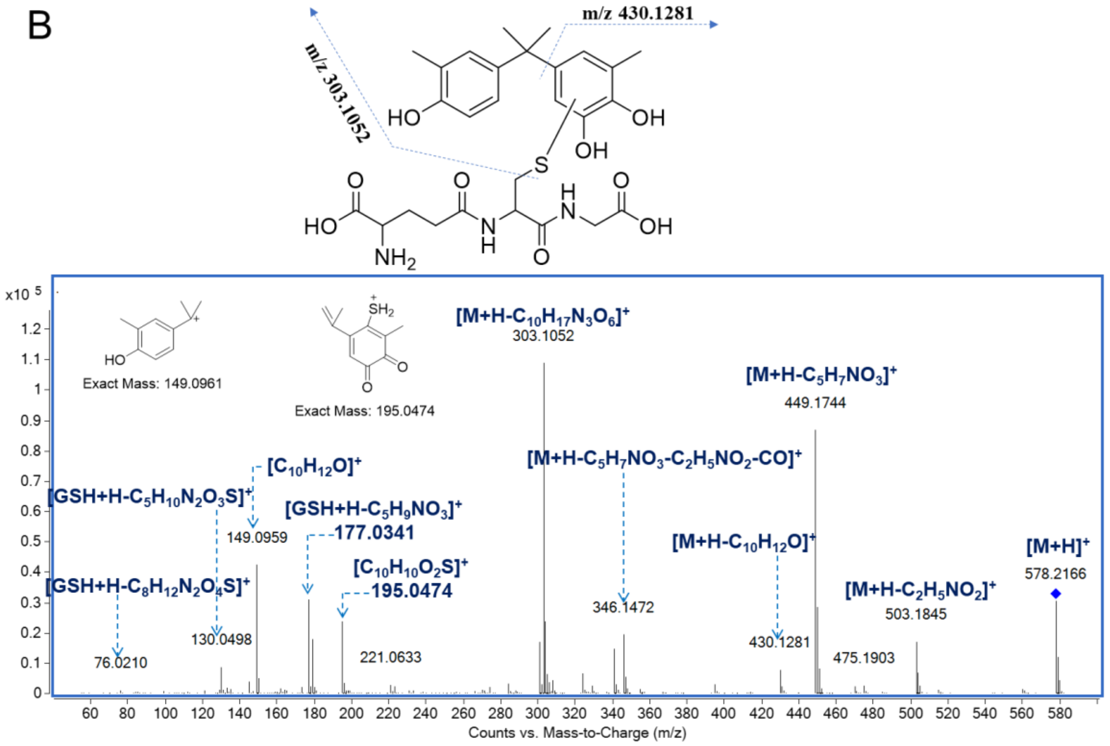
m/z 430.1281
m/z 303.1052
x10 5
Exact Mass: 149.0961
Exact Mass: 195.0474
[M+H-C10H17N3O6]+
303.1052
[M+H-C5H7NO3]+
449.1744
[M+H-C5H7NO3-C2H5NO2-CO]+
[C10H12O]+
[GSH+H-C5H10N2O3S]+
149.0959
[GSH+H-C5H9NO3]+
177.0341
[M+H-C10H12O]+
[M+H]+
578.2166
[C10H10O2S]+
195.0474
[GSH+H-C8H12N2O4S]+
[M+H-C2H5NO2]+
503.1845
346.1472
130.0498
430.1281
221.0633
475.1903
76.0210
Counts vs. Mass-to-Charge (m/z)

C

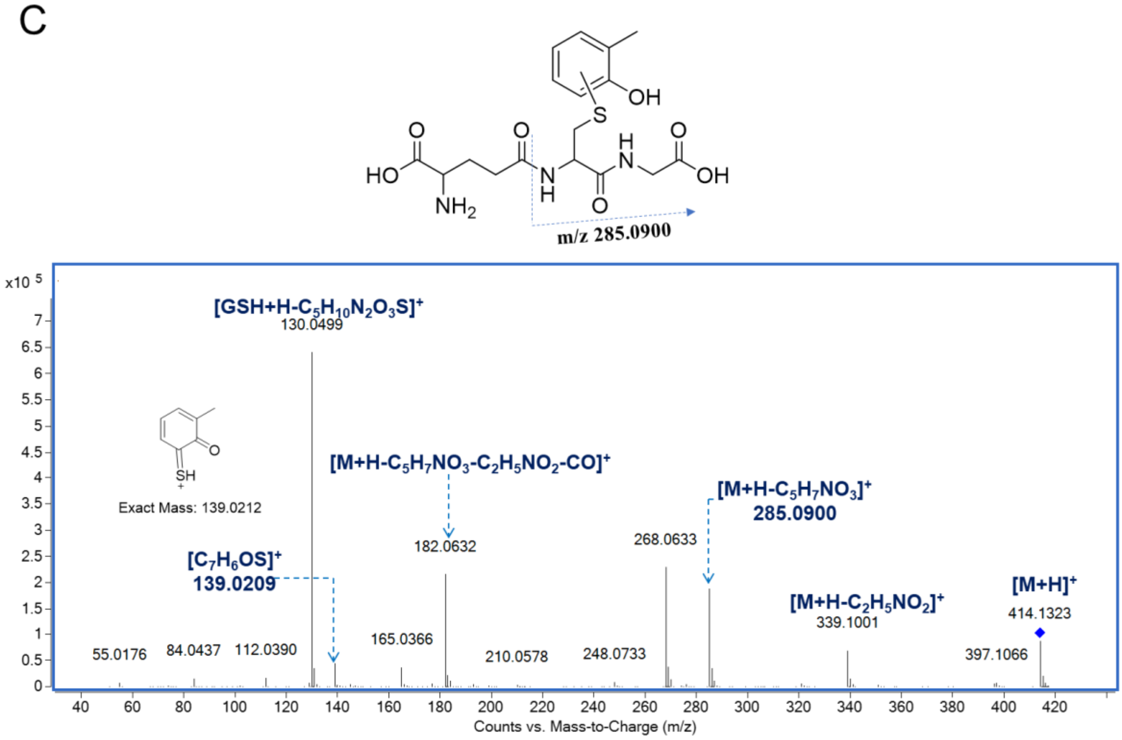

HO
NH2
OH
S
N
H
m/z 285.0900
x10 5
[GSH+H-C5H10N2O3S]+
130.0499
Exact Mass: 139.0212
[M+H-C5H7NO3-C2H5NO2-CO]+
182.0632
[C7H6OS]+
139.0209
268.0633
[M+H-C5H7NO3]+
285.0900
[M+H-C2H5NO2]+
339.1001
[M+H]+
414.1323
55.0176
84.0437
112.0390
165.0366
210.0578
248.0733
397.1066
Counts vs. Mass-to-Charge (m/z)

D

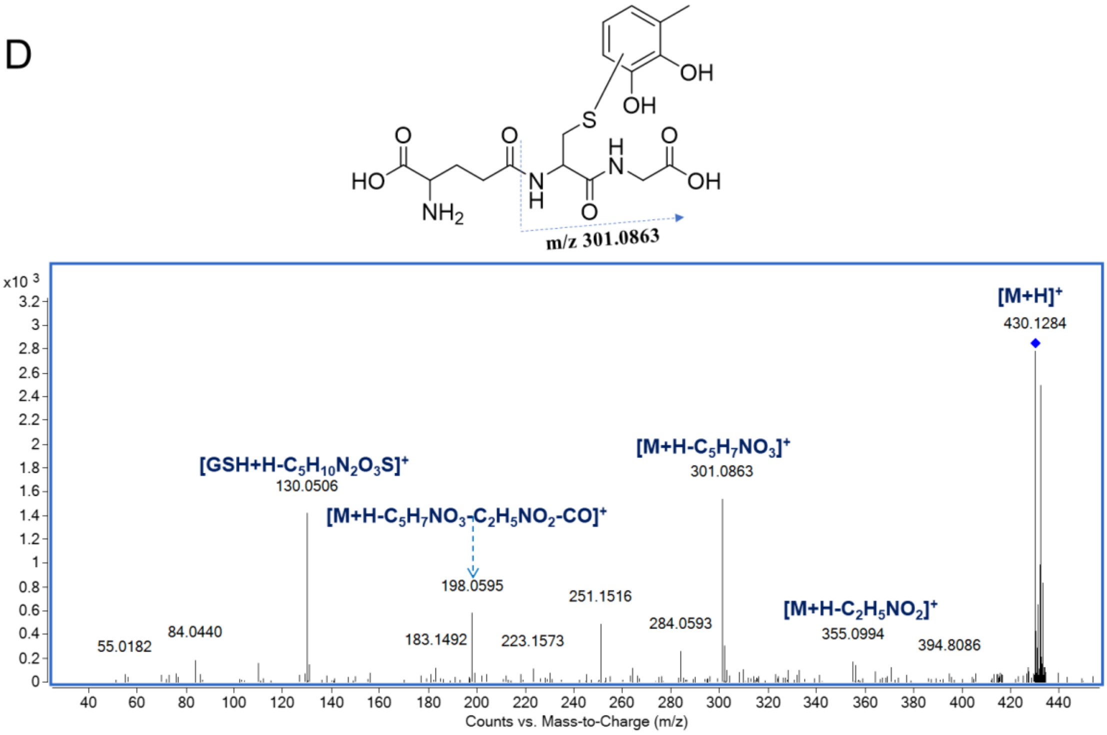

HO
NH2
OH
S
N
H
m/z 301.0863
x10 3
[M+H]+
430.1284
[GSH+H-C5H10N2O3S]+
130.0506
[M+H-C5H7NO3]+
301.0863
[M+H-C5H7NO3-C2H5NO2-CO]+
198.0595
251.1516
284.0593
[M+H-C2H5NO2]+
355.0994
394.8086
55.0182
84.0440
183.1492
223.1573
Counts vs. Mass-to-Charge (m/z)

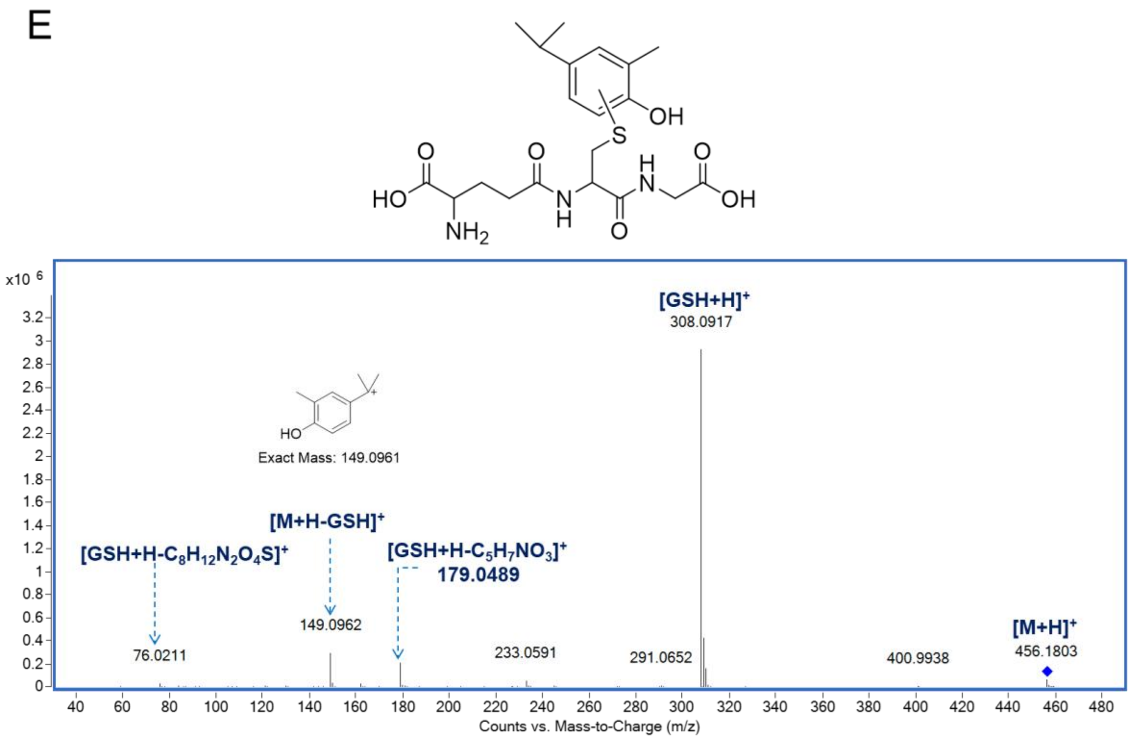

Figure S10. MS/MS spectrum and possible structure of BPC RM-GSH adducts in positive mode. (A) CG1 and CG2, (B) CG3, (C) CG4, (D) CG5, (E) CG6.

A

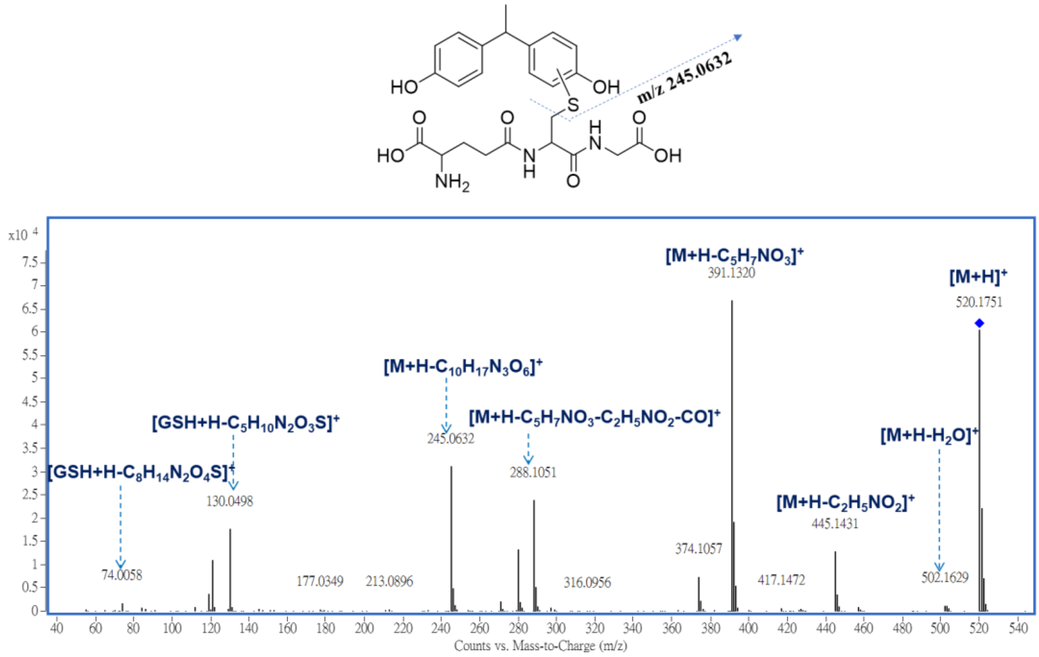

m/z 245.0632
HO
OH
S
NH2
[M+H-C5H7NO3]+
391.1320
[M+H]+
520.1751
[M+H-C10H17N3O6]+
245.0632
[M+H-C5H7NO3-C2H5NO2-CO]+
288.1051
[GSH+H-C5H10N2O3S]+
130.0498
[GSH+H-C8H14N2O4S]+
74.0058
[M+H-H2O]+
502.1629
[M+H-C2H5NO2]+
445.1431
374.1057
417.1472
177.0349
213.0896
316.0956
x10 4
Counts vs. Mass-to-Charge (m/z)

B

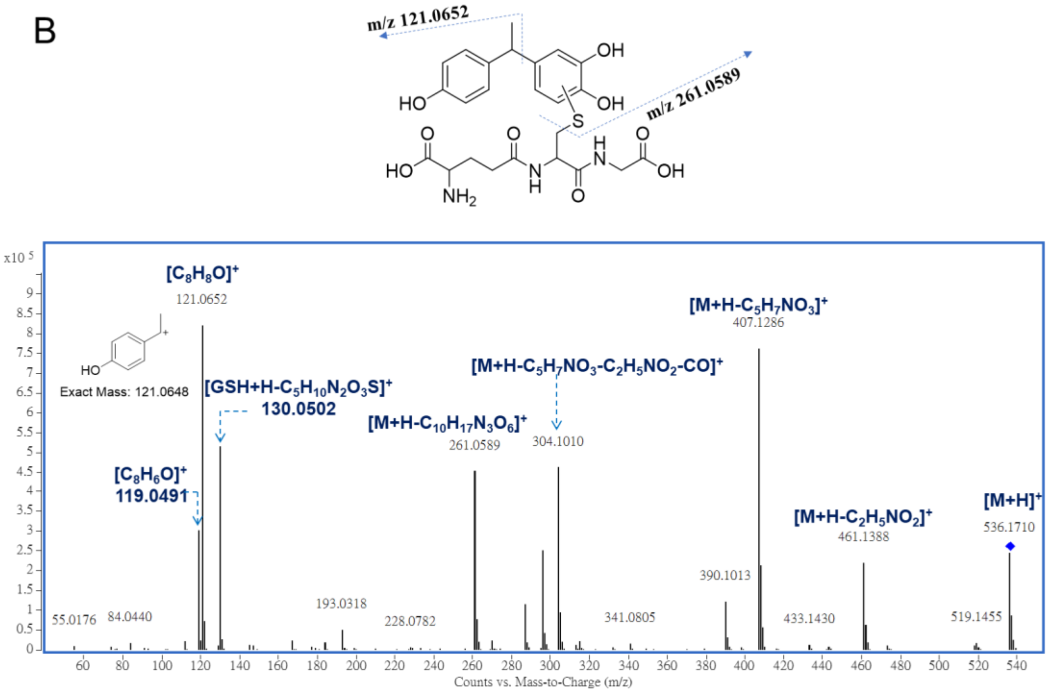

m/z 121.0652
m/z 261.0589
HO
OH
S
NH2
[C8H9O]+
121.0652
Exact Mass: 121.0648
[GSH+H-C5H10N2O3S]+
130.0502
[C8H6O]+
119.0491
[M+H-C10H17N3O6]+
261.0589
[M+H-C5H7NO3-C2H5NO2-CO]+
304.1010
[M+H-C5H7NO3]+
407.1286
[M+H-C2H5NO2]+
461.1388
[M+H]+
536.1710
390.1013
433.1430
519.1455
341.0805
228.0782
193.0318
55.0176
84.0440
x10 5
Counts vs. Mass-to-Charge (m/z)

C

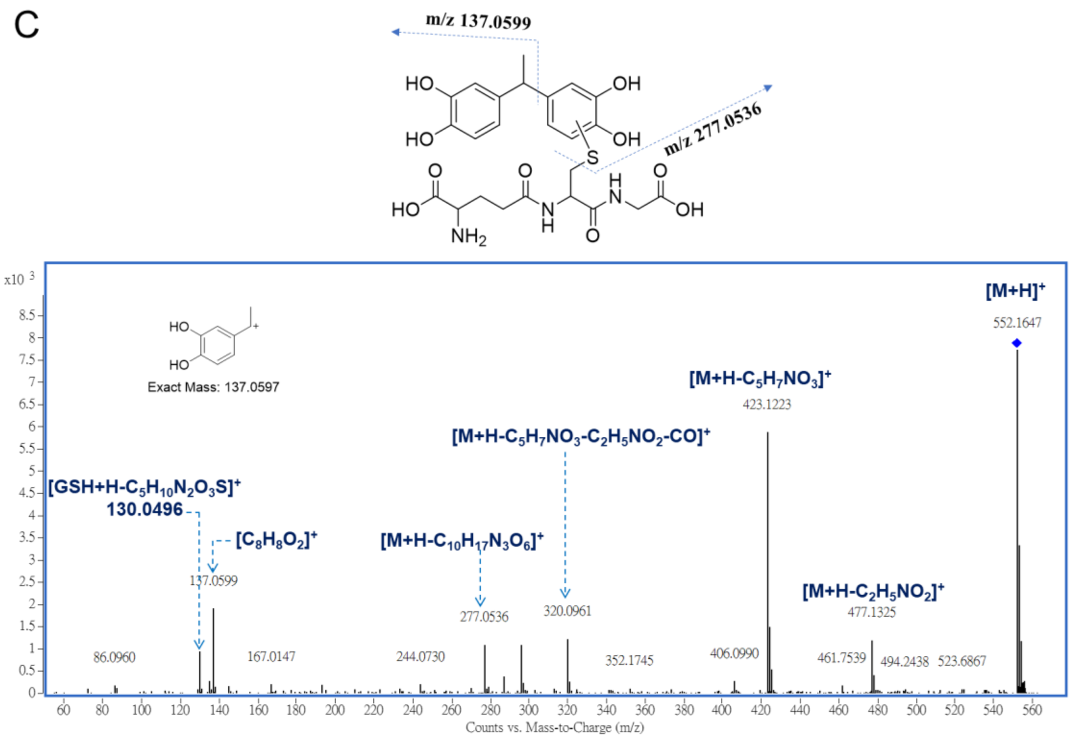
m/z 137.0599
m/z 277.0536
Exact Mass: 137.0597
[M+H]+
552.1647
[M+H-C5H7NO3]+
423.1223
[M+H-C5H7NO3-C2H5NO2-CO]+
[GSH+H-C5H10N2O3S]+
130.0496
[C8H8O2]+
[M+H-C10H17N3O6]+
137.0599
277.0536
320.0961
[M+H-C2H5NO2]+
477.1325
86.0960
167.0147
244.0730
352.1745
406.0990
461.7539
494.2438
523.6867
Counts vs. Mass-to-Charge (m/z)

D

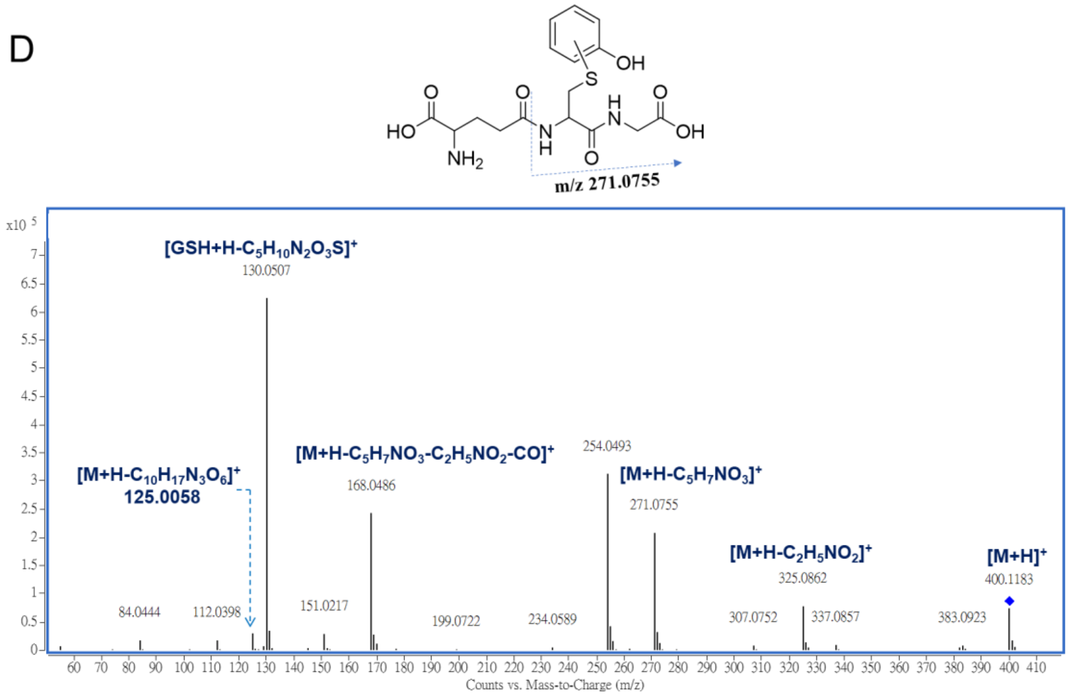
m/z 271.0755
[GSH+H-C5H10N2O3S]+
130.0507
[M+H-C5H7NO3-C2H5NO2-CO]+
254.0493
[M+H-C10H17N3O6]+
125.0058
168.0486
[M+H-C5H7NO3]+
271.0755
[M+H-C2H5NO2]+
325.0862
[M+H]+
400.1183
84.0444
112.0398
151.0217
199.0722
234.0589
307.0752
337.0857
383.0923
Counts vs. Mass-to-Charge (m/z)

E

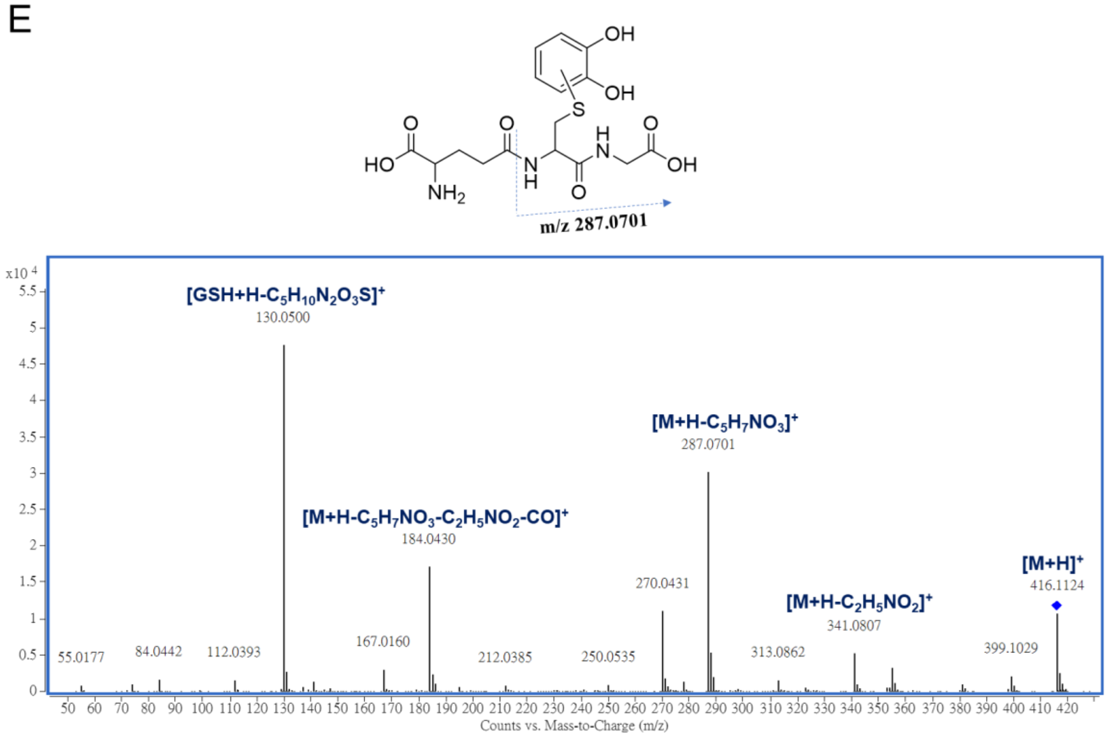

F

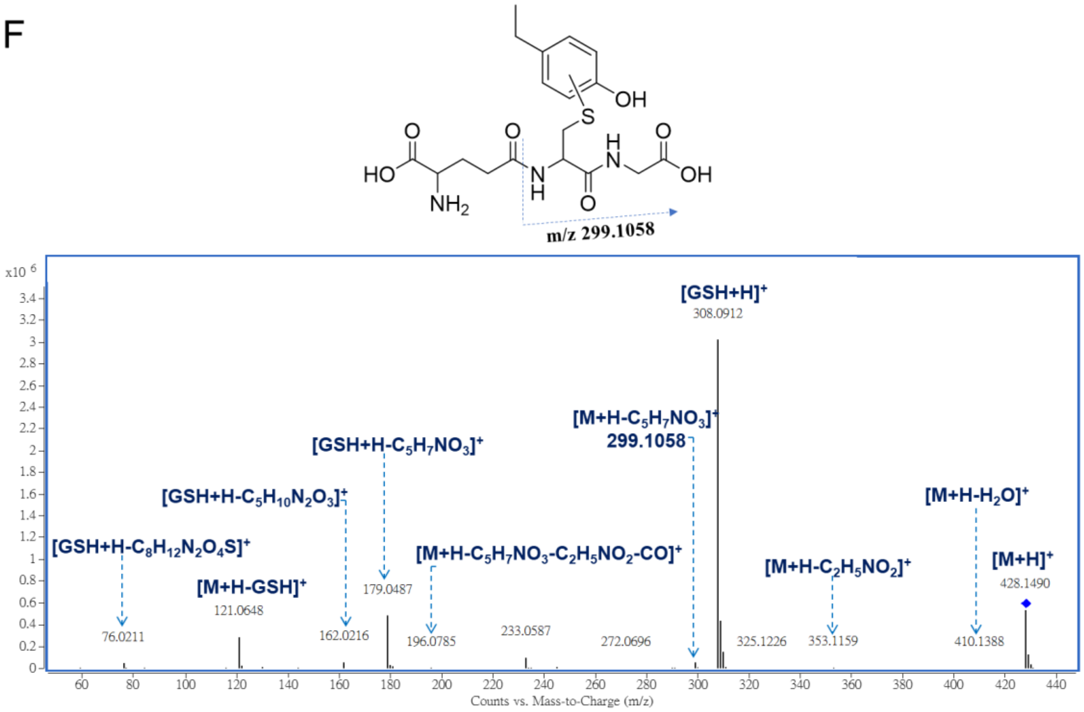

Figure S11. MS/MS spectrum and possible structure of BPE RM-GSH adducts in positive mode. (A) EG1 and EG2, (B) EG3, (C) EG4, (D) EG5, (E) EG6, (F) EG7 and EG8.

A

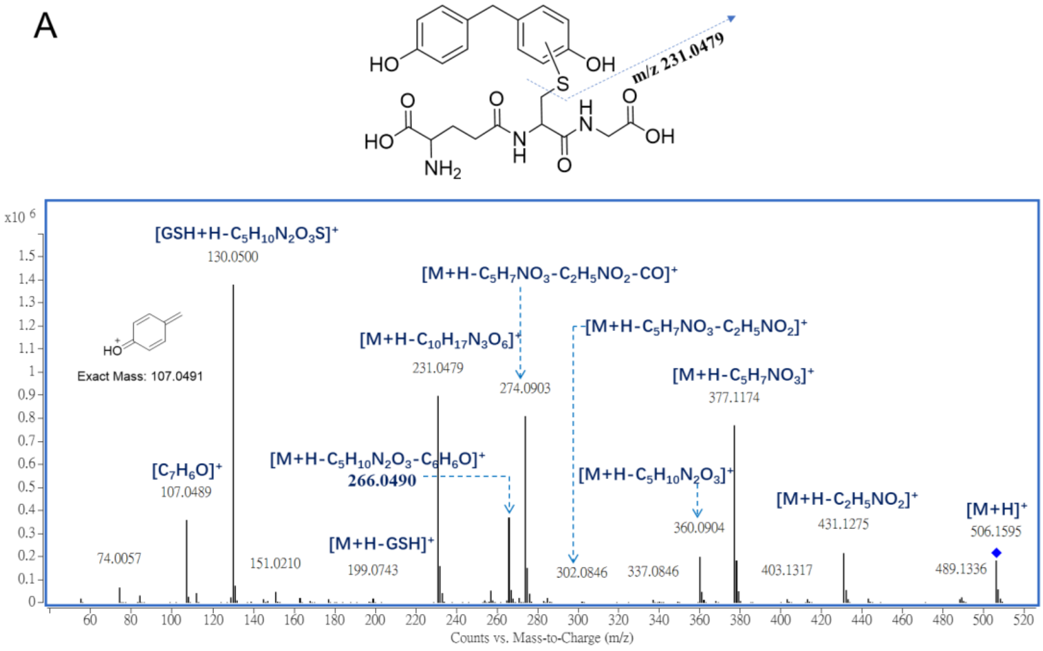
HO
OH
S
m/z 231.0479
O
HO
NH2
O
N
H
H
N
O
O
OH
x10 6
$[GSH+H-C_5H_{10}N_2O_3S]^+$
130.0500
$[M+H-C_5H_7NO_3-C_2H_5NO_2-CO]^+$
$[M+H-C_5H_7NO_3-C_2H_5NO_2]^+$
$[M+H-C_{10}H_{17}N_3O_6]^+$
231.0479
274.0903
$[M+H-C_5H_7NO_3]^+$
377.1174
HO
Exact Mass: 107.0491
$[C_7H_6O]^+$
107.0489
$[M+H-C_5H_{10}N_2O_3-C_6H_6O]^+$
266.0490
$[M+H-C_5H_{10}N_2O_3]^+$
$[M+H-C_2H_5NO_2]^+$
431.1275
$[M+H]^+$
506.1595
360.0904
$[M+H-GSH]^+$
74.0057
151.0210
199.0743
302.0846
337.0846
403.1317
489.1336
Counts vs. Mass-to-Charge (m/z)

B

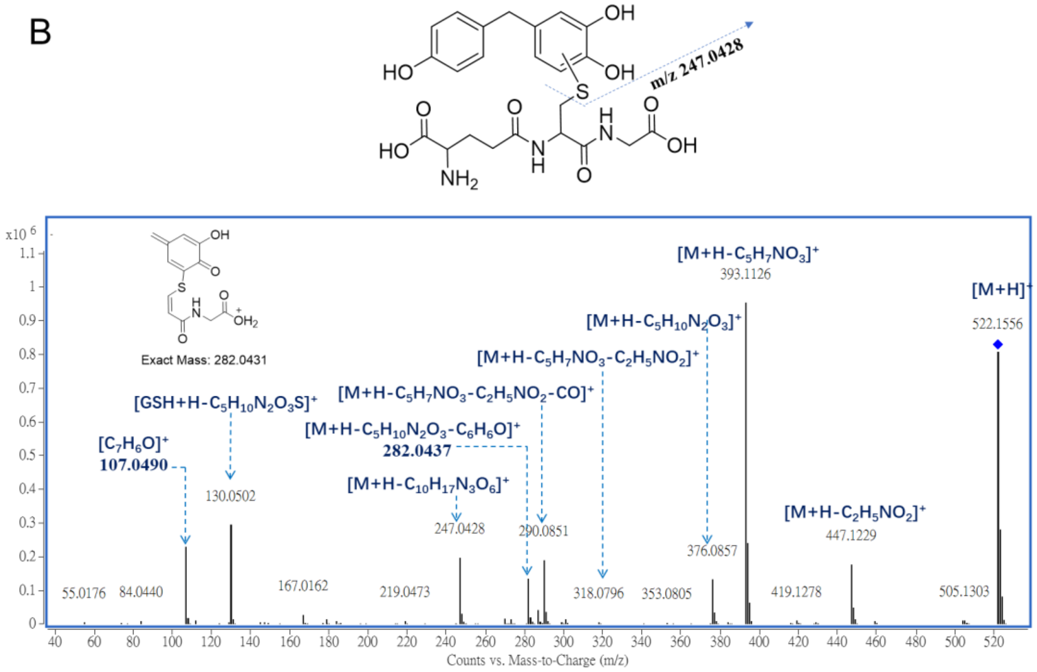
OH
HO
OH
S
m/z 247.0428
O
HO
NH2
O
N
H
H
N
O
O
OH
x10 6
OH
O
S
H
N
O
OH_2
O
Exact Mass: 282.0431
$[M+H-C_5H_7NO_3]^+$
393.1126
$[M+H]^+$
522.1556
$[M+H-C_5H_{10}N_2O_3]^+$
$[M+H-C_5H_7NO_3-C_2H_5NO_2]^+$
$[M+H-C_5H_7NO_3-C_2H_5NO_2-CO]^+$
$[GSH+H-C_5H_{10}N_2O_3S]^+$
$[M+H-C_5H_{10}N_2O_3-C_6H_6O]^+$
$[C_7H_6O]^+$
107.0490
282.0437
$[M+H-C_{10}H_{17}N_3O_6]^+$
130.0502
247.0428
290.0851
$[M+H-C_2H_5NO_2]^+$
447.1229
376.0857
55.0176
84.0440
167.0162
219.0473
318.0796
353.0805
419.1278
505.1303
Counts vs. Mass-to-Charge (m/z)

C

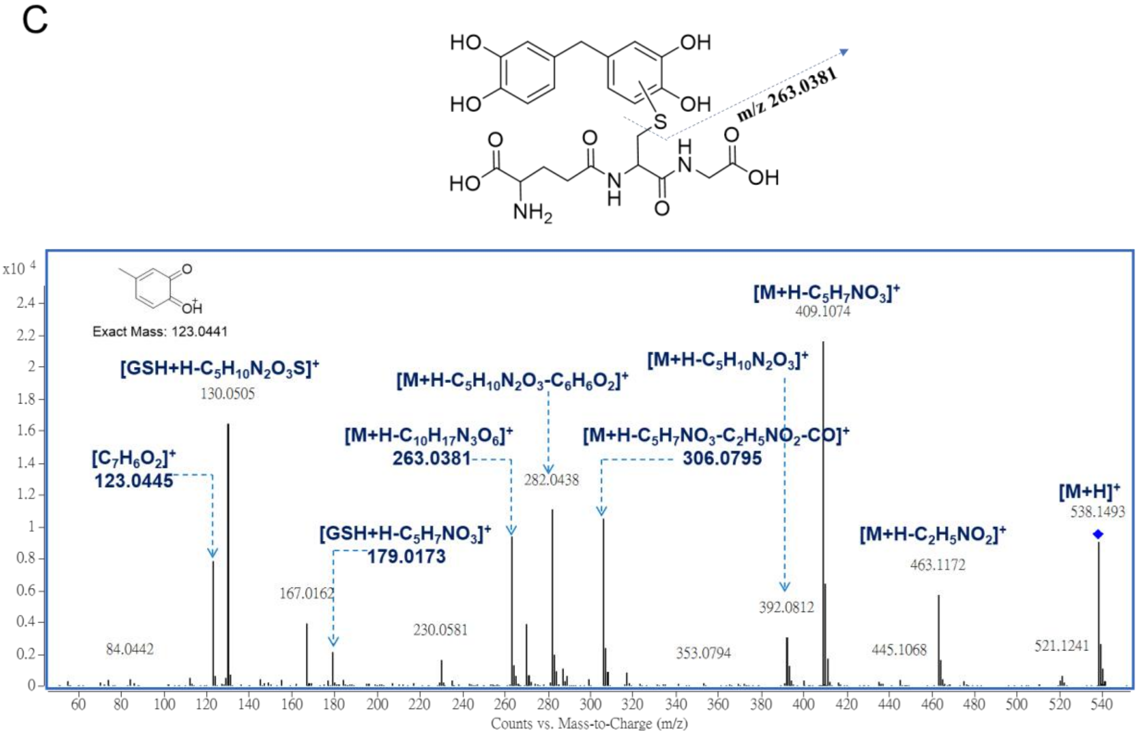

HO
HO
OH
OH
S
m/z 263.0381
O
O
H
N
O
HO
N
H
OH
NH2
O
x10 4
Exact Mass: 123.0441
[GSH+H-C5H10N2O3S]+
130.0505
[C7H6O2]+
123.0445
[M+H-C5H10N2O3-C6H6O2]+
[M+H-C10H17N3O6]+
263.0381
282.0438
[M+H-C5H10N2O3]+
[M+H-C5H7NO3-C2H5NO2-CO]+
306.0795
[M+H-C5H7NO3]+
409.1074
[GSH+H-C5H7NO3]+
179.0173
167.0162
230.0581
84.0442
353.0794
392.0812
445.1068
[M+H-C2H5NO2]+
463.1172
521.1241
[M+H]+
538.1493
Counts vs. Mass-to-Charge (m/z)

D

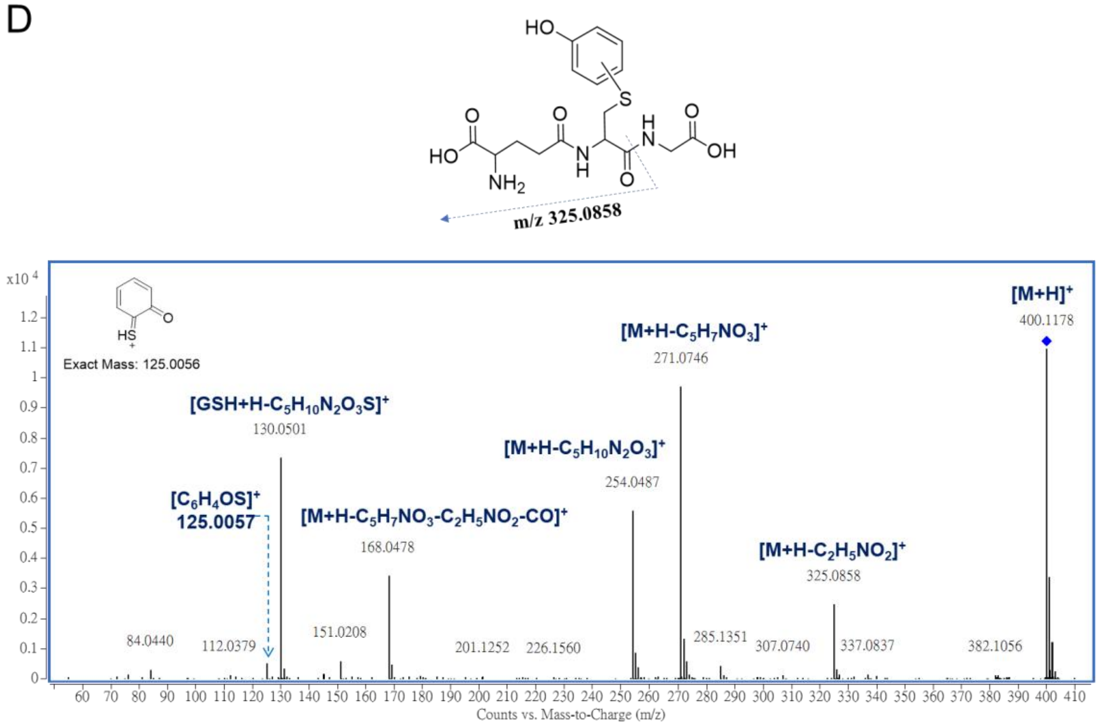

HO
S
O
O
H
N
O
HO
N
H
OH
NH2
O
m/z 325.0858
x10 4
HS
Exact Mass: 125.0056
[GSH+H-C5H10N2O3S]+
130.0501
[C6H4OS]+
125.0057
[M+H-C5H7NO3-C2H5NO2-CO]+
168.0478
[M+H-C5H10N2O3]+
254.0487
[M+H-C5H7NO3]+
271.0746
[M+H-C2H5NO2]+
325.0858
[M+H]+
400.1178
84.0440
112.0379
151.0208
201.1252
226.1560
285.1351
307.0740
337.0837
382.1056
Counts vs. Mass-to-Charge (m/z)

E

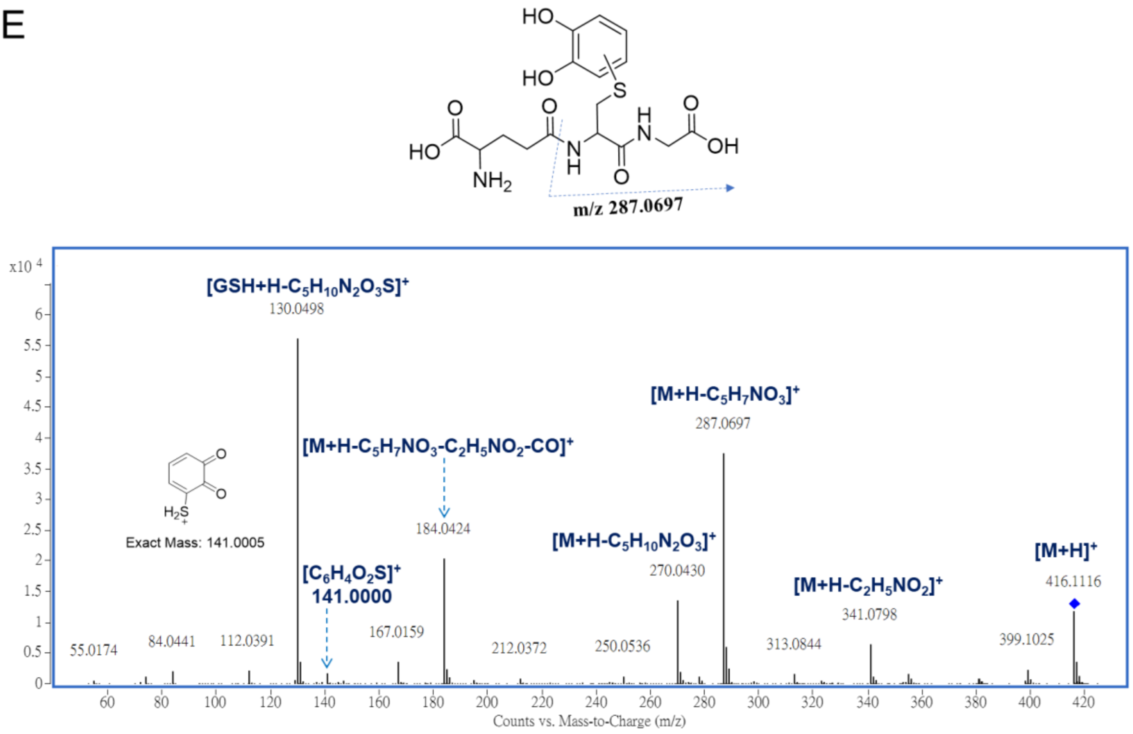

HO
HO
S
O
O
O
HO
NH2
N
H
H
N
O
OH
m/z 287.0697
x10 4
[GSH+H-C5H10N2O3S]+
130.0498
[M+H-C5H7NO3]+
287.0697
[M+H-C5H7NO3-C2H5NO2-CO]+
184.0424
H2S
Exact Mass: 141.0005
[M+H-C5H10N2O3]+
270.0430
[M+H]+
416.1116
[C6H4O2S]+
141.0000
[M+H-C2H5NO2]+
341.0798
55.0174
84.0441
112.0391
167.0159
212.0372
250.0536
313.0844
399.1025
Counts vs. Mass-to-Charge (m/z)

F

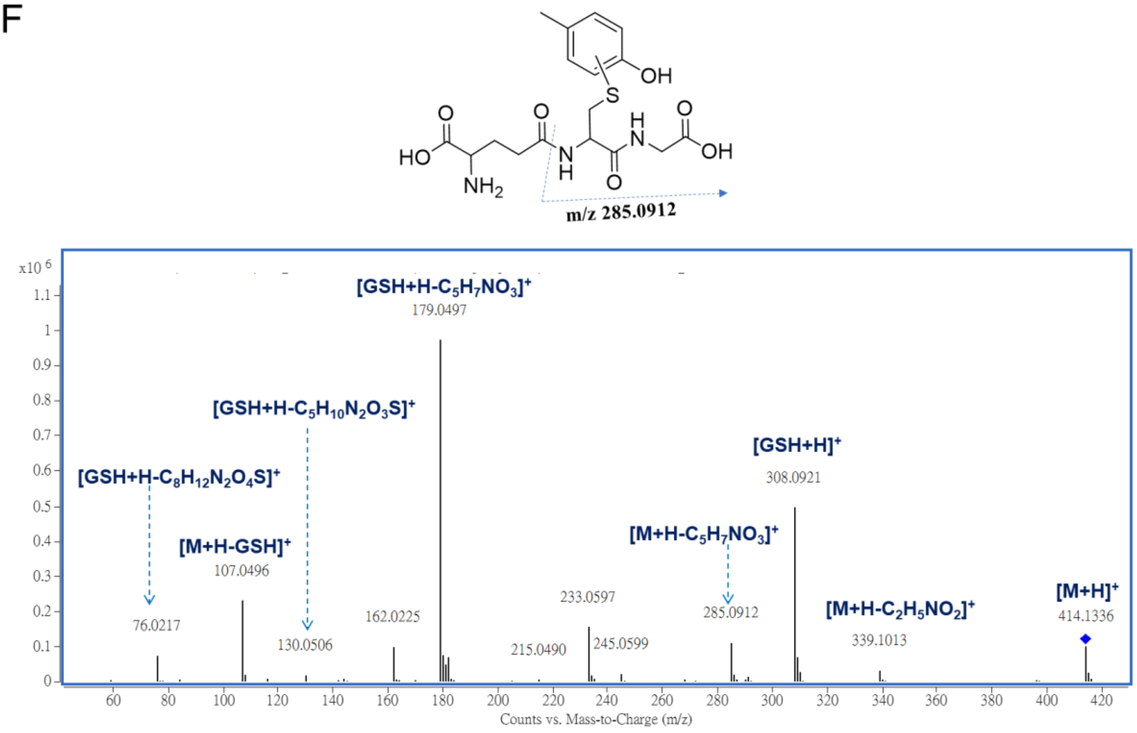

OH
S
O
O
O
HO
NH2
N
H
H
N
O
OH
m/z 285.0912
x10 6
[GSH+H-C5H7NO3]+
179.0497
[GSH+H-C5H10N2O3S]+
[GSH+H]+
308.0921
[GSH+H-C8H12N2O4S]+
[M+H-GSH]+
107.0496
[M+H-C5H7NO3]+
233.0597
285.0912
[M+H]+
414.1336
[M+H-C2H5NO2]+
339.1013
76.0217
130.0506
162.0225
215.0490
245.0599
Counts vs. Mass-to-Charge (m/z)

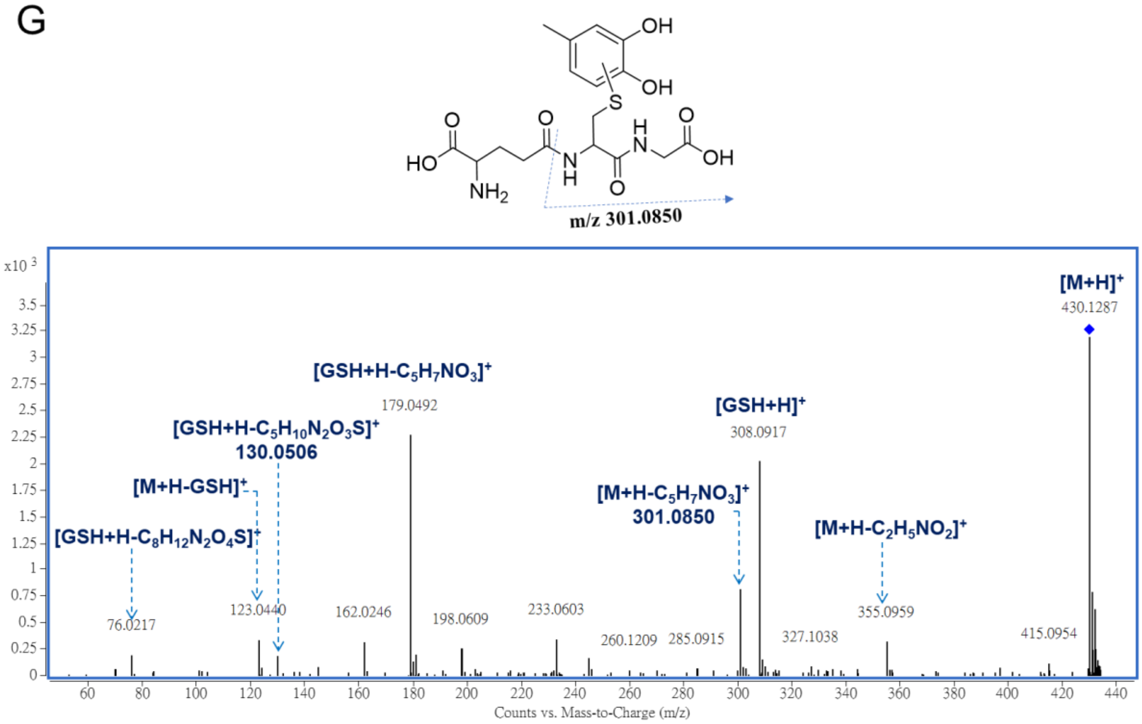

Figure S12. MS/MS spectrum and possible structure of BPF RM-GSH adducts in positive mode. (A) FG1 and FG2, (B) FG3 and FG4, (C) FG5, (D) FG6, (E) FG7, (F) FG8, (G) FG9.

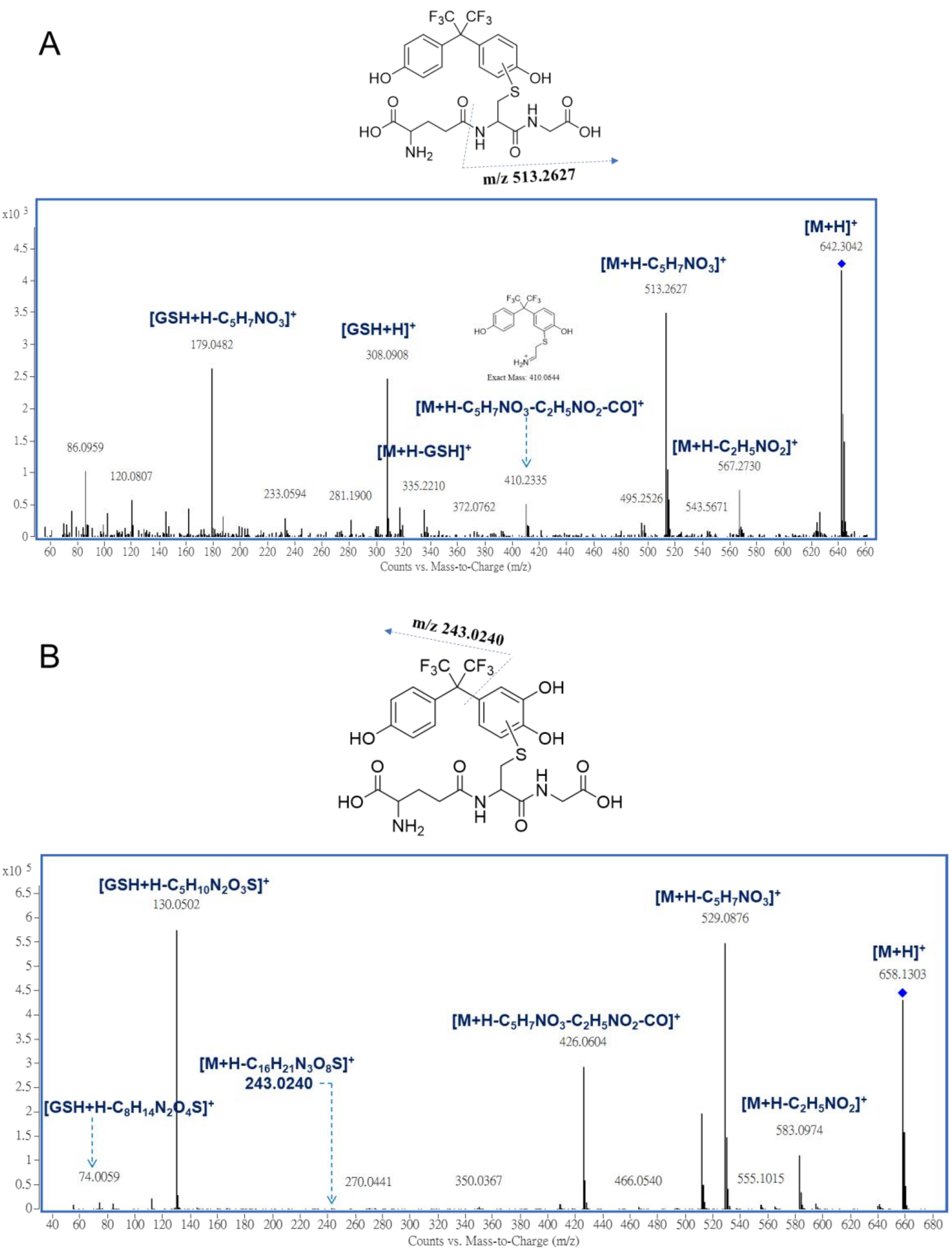
A
m/z 513.2627
x10 3
[M+H]+
642.3042
[M+H-C5H7NO3]+
513.2627
[GSH+H-C5H7NO3]+
179.0482
[GSH+H]+
308.0908
Exact Mass: 410.0644
[M+H-C5H7NO3-C2H5NO2-CO]+
410.2335
[M+H-GSH]+
[M+H-C2H5NO2]+
567.2730
86.0959
120.0807
233.0594
281.1900
335.2210
372.0762
495.2526
543.5671
Counts vs. Mass-to-Charge (m/z)
B
m/z 243.0240
x10 5
[GSH+H-C5H10N2O3S]+
130.0502
[M+H-C5H7NO3]+
529.0876
[M+H]+
658.1303
[M+H-C5H7NO3-C2H5NO2-CO]+
426.0604
[M+H-C16H21N3O8S]+
243.0240
[GSH+H-C8H14N2O4S]+
74.0059
[M+H-C2H5NO2]+
583.0974
270.0441
350.0367
466.0540
555.1015
Counts vs. Mass-to-Charge (m/z)

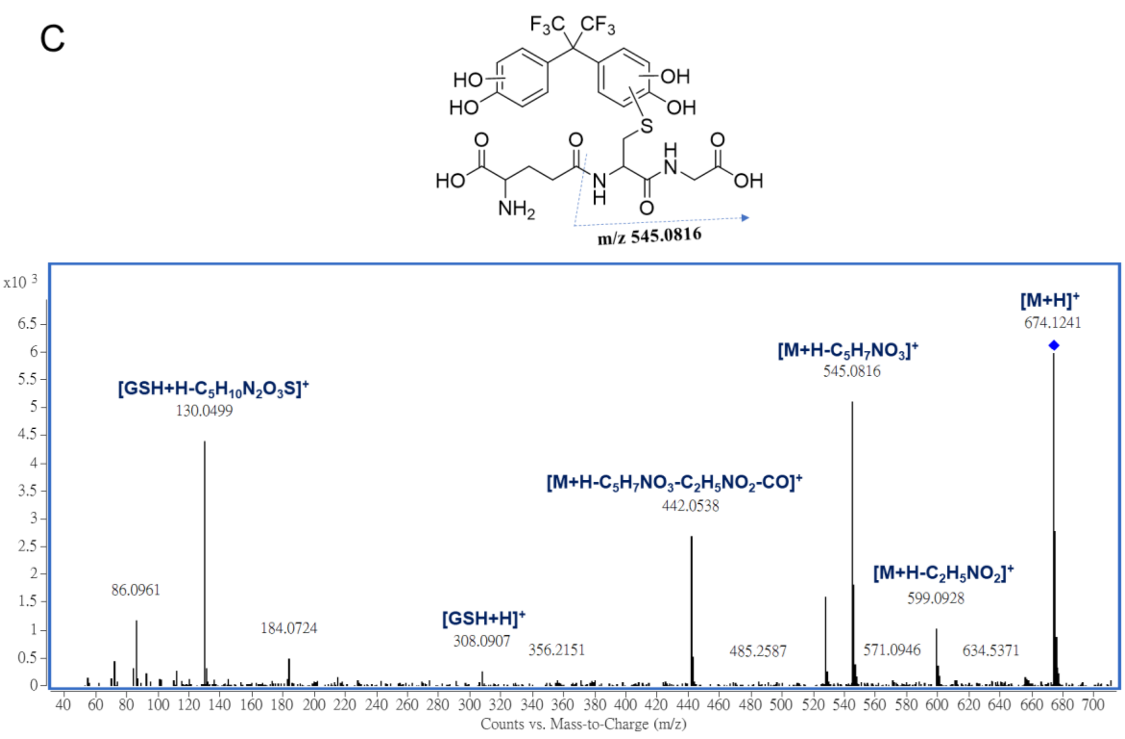

Figure S13. MS/MS spectrum and possible structure of BPAF RM-GSH adducts in positive mode. (A) AFG1, (B) AFG2, (C) AFG3.

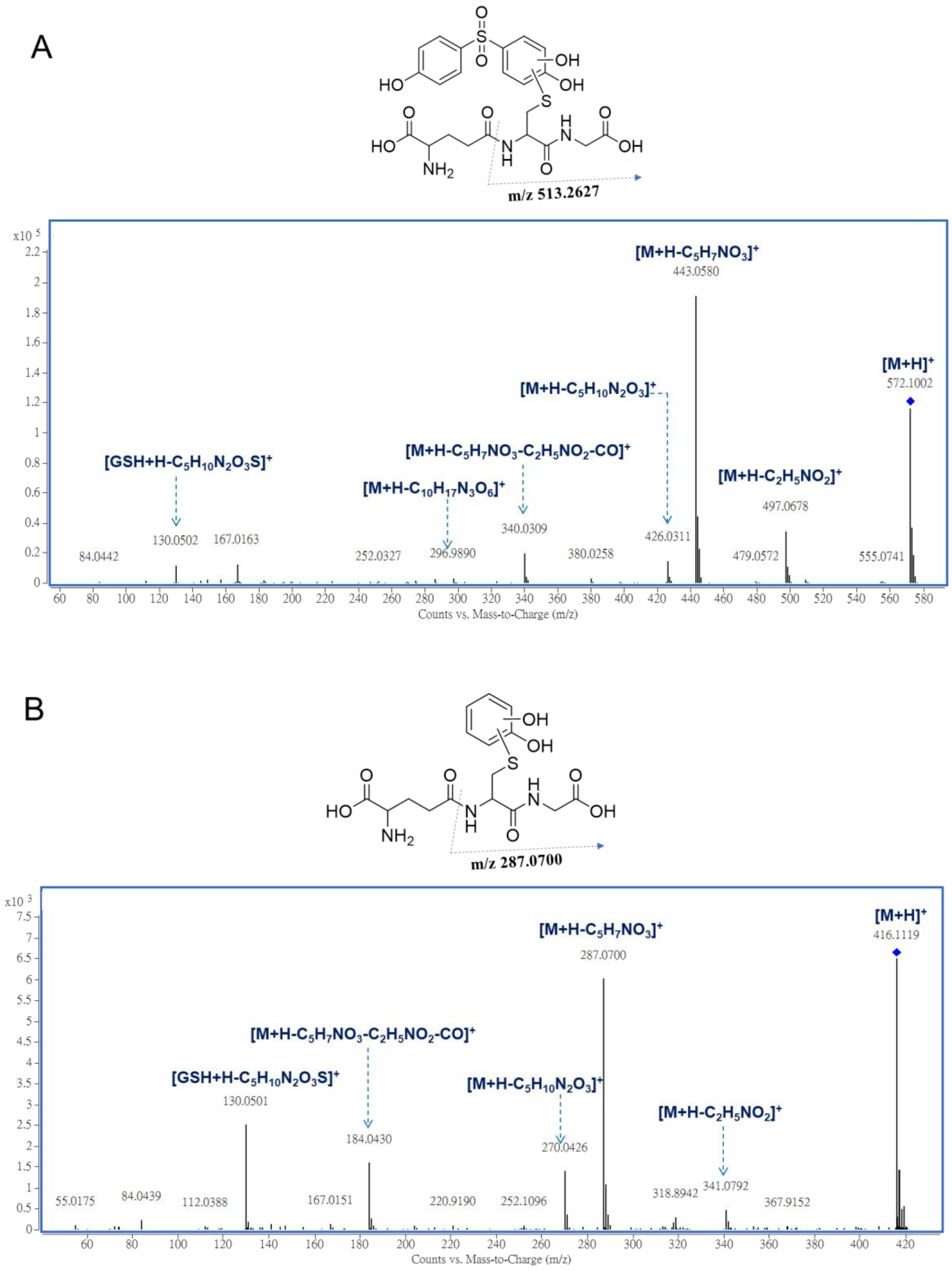

Figure S14. MS/MS spectrum and possible structure of BPS RM-GSH adducts in positive mode. (A) SG1, (B) SG2.

A

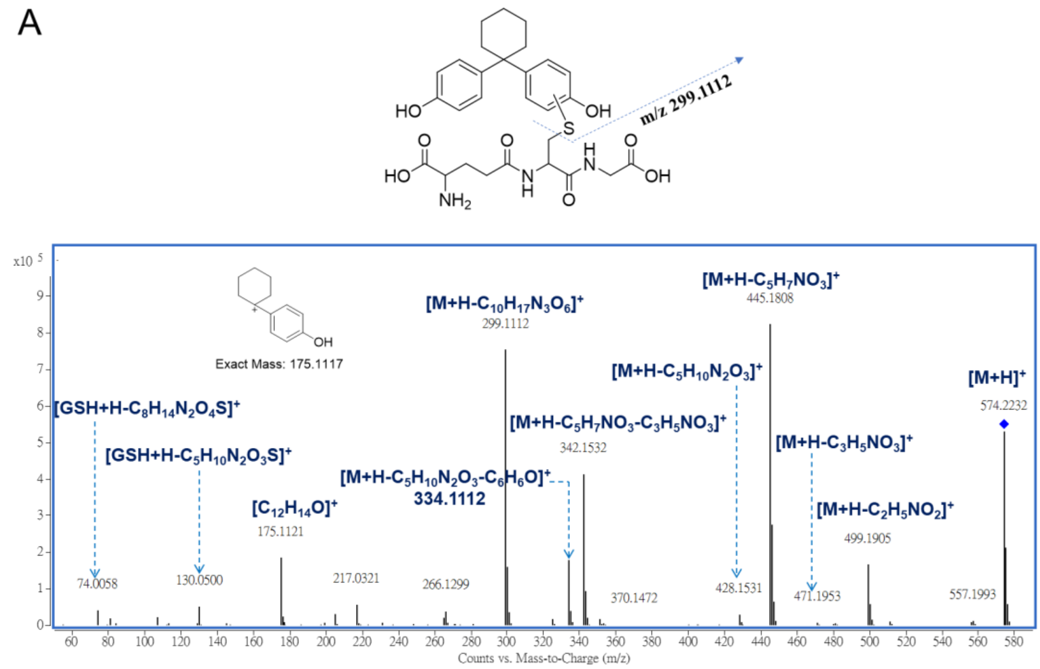
m/z 299.1112
x10 5
Exact Mass: 175.1117
[M+H-C10H17N3O6]+
299.1112
[M+H-C5H7NO3]+
445.1808
[M+H-C5H10N2O3]+
[M+H]+
574.2232
[GSH+H-C8H14N2O4S]+
[M+H-C5H7NO3-C3H5NO3]+
342.1532
[GSH+H-C5H10N2O3S]+
[M+H-C3H5NO3]+
[M+H-C5H10N2O3-C6H6O]+
334.1112
[C12H14O]+
175.1121
[M+H-C2H5NO2]+
499.1905
74.0058
130.0500
217.0321
266.1299
370.1472
428.1531
471.1953
557.1993
Counts vs. Mass-to-Charge (m/z)

B

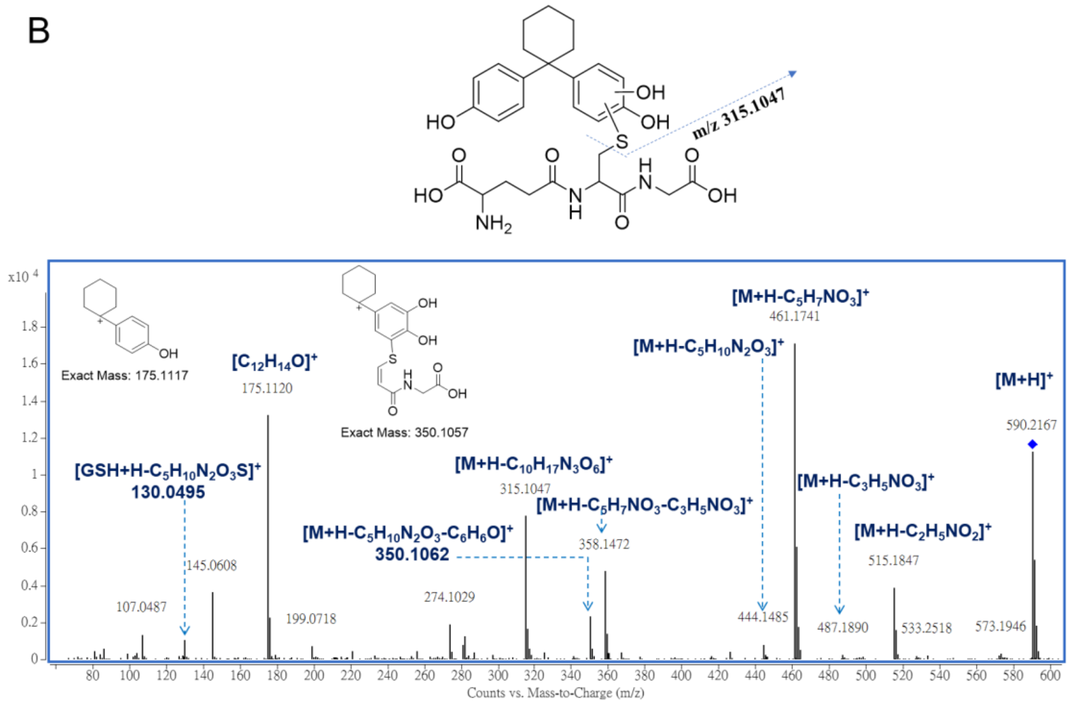
m/z 315.1047
x10 4
Exact Mass: 175.1117
Exact Mass: 350.1057
[C12H14O]+
175.1120
[M+H-C5H7NO3]+
461.1741
[M+H-C5H10N2O3]+
[M+H]+
590.2167
[GSH+H-C5H10N2O3S]+
130.0495
[M+H-C10H17N3O6]+
315.1047
[M+H-C5H7NO3-C3H5NO3]+
358.1472
[M+H-C3H5NO3]+
[M+H-C5H10N2O3-C6H6O]+
350.1062
[M+H-C2H5NO2]+
515.1847
145.0608
107.0487
199.0718
274.1029
444.1485
487.1890
533.2518
573.1946
Counts vs. Mass-to-Charge (m/z)

C

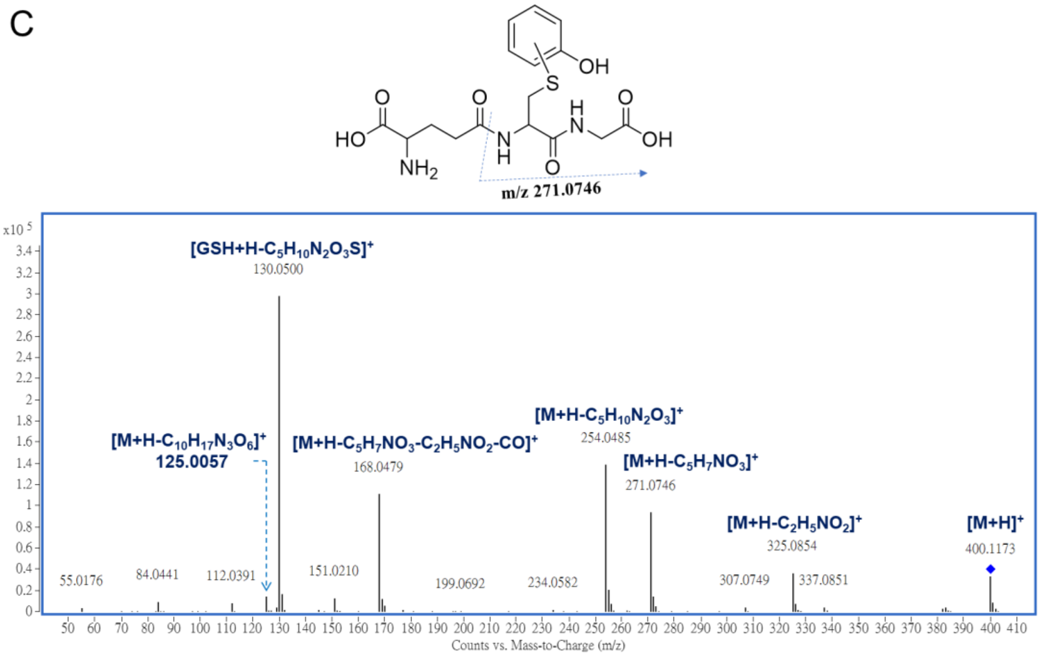

OH
S
HO
O
O
H
N
O
OH
N
H
NH2
O
m/z 271.0746
x10 5
[GSH+H-C5H10N2O3S]+
130.0500
[M+H-C10H17N3O6]+
125.0057
[M+H-C5H7NO3-C2H5NO2-CO]+
168.0479
[M+H-C5H10N2O3]+
254.0485
[M+H-C5H7NO3]+
271.0746
[M+H-C2H5NO2]+
325.0854
[M+H]+
400.1173
55.0176
84.0441
112.0391
151.0210
199.0692
234.0582
307.0749
337.0851
Counts vs. Mass-to-Charge (m/z)

D

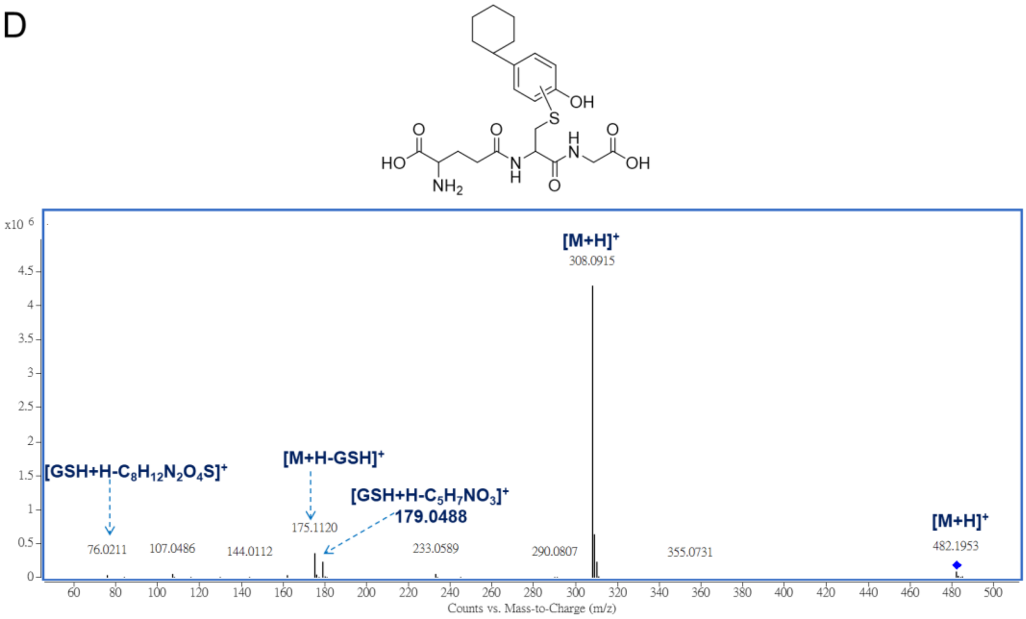

OH
S
HO
NH2
O
O
H
N
N
H
O
O
OH
x10 6
[M+H]+
308.0915
[GSH+H-C8H12N2O4S]+
[M+H-GSH]+
[GSH+H-C5H7NO3]+
179.0488
175.1120
76.0211
107.0486
144.0112
233.0589
290.0807
355.0731
[M+H]+
482.1953
Counts vs. Mass-to-Charge (m/z)

E

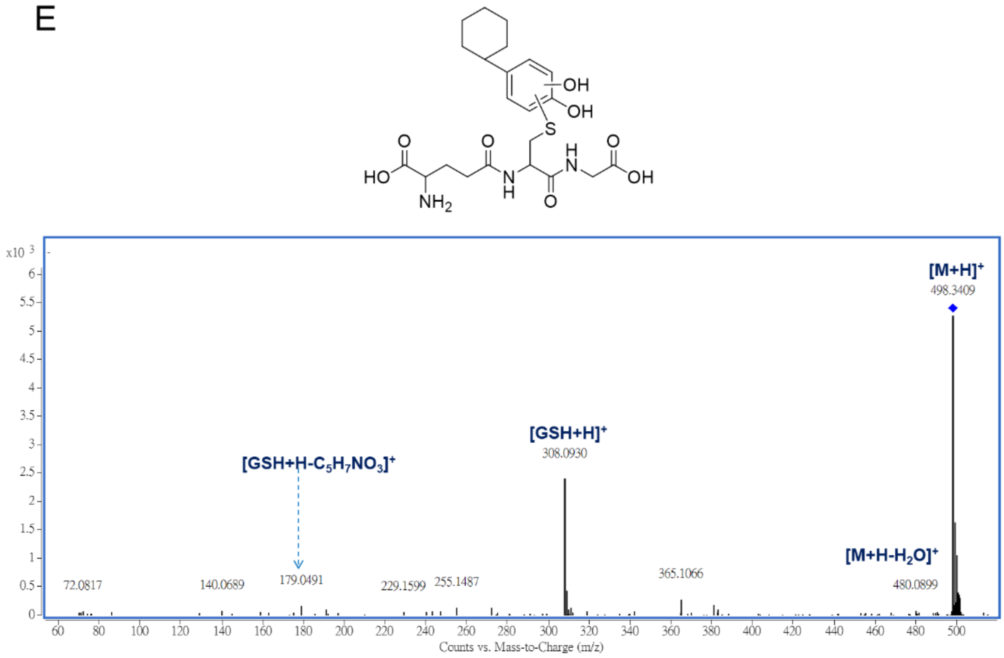

Figure S15. MS/MS spectrum and possible structure of BPZ RM-GSH adducts in positive mode. (A) ZG1, (B) ZG2-4, (C) ZG5, (D) ZG6, (E) ZG7-8.

A

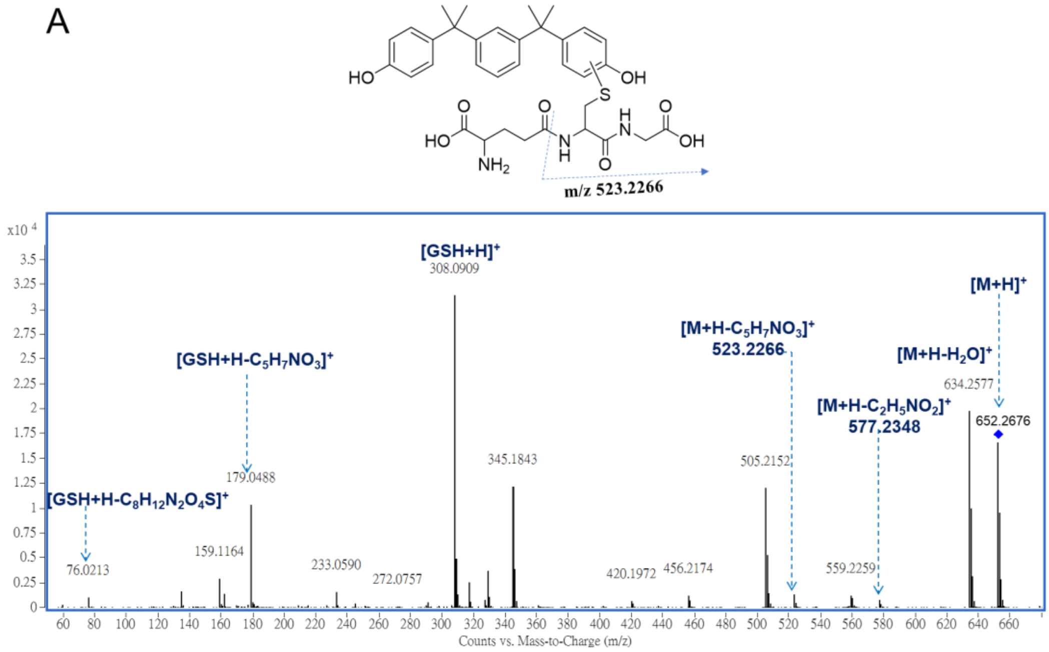
m/z 523.2266
[GSH+H]+
308.0909
[M+H]+
[M+H-C5H7NO3]+
523.2266
[M+H-H2O]+
634.2577
[GSH+H-C5H7NO3]+
[M+H-C2H5NO2]+
577.2348
652.2676
345.1843
505.2152
179.0488
[GSH+H-C8H12N2O4S]+
159.1164
76.0213
233.0590
272.0757
420.1972
456.2174
559.2259
Counts vs. Mass-to-Charge (m/z)

B

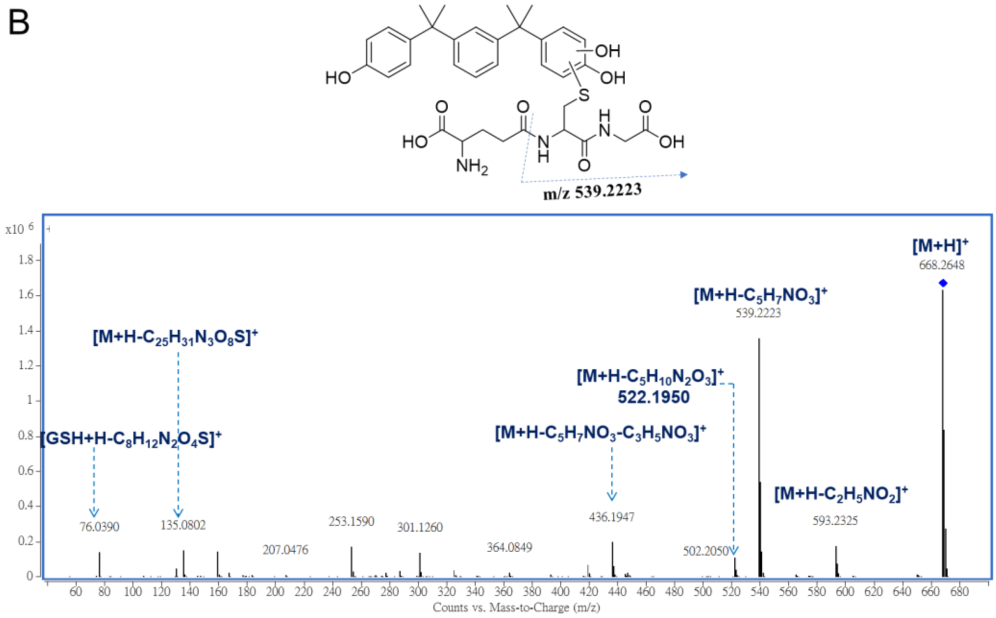
m/z 539.2223
[M+H]+
668.2648
[M+H-C5H7NO3]+
539.2223
[M+H-C25H31N3O8S]+
[M+H-C5H10N2O3]+
522.1950
[GSH+H-C8H12N2O4S]+
[M+H-C5H7NO3-C3H5NO3]+
[M+H-C2H5NO2]+
76.0390
135.0802
253.1590
301.1260
207.0476
364.0849
436.1947
502.2050
593.2325
Counts vs. Mass-to-Charge (m/z)

C

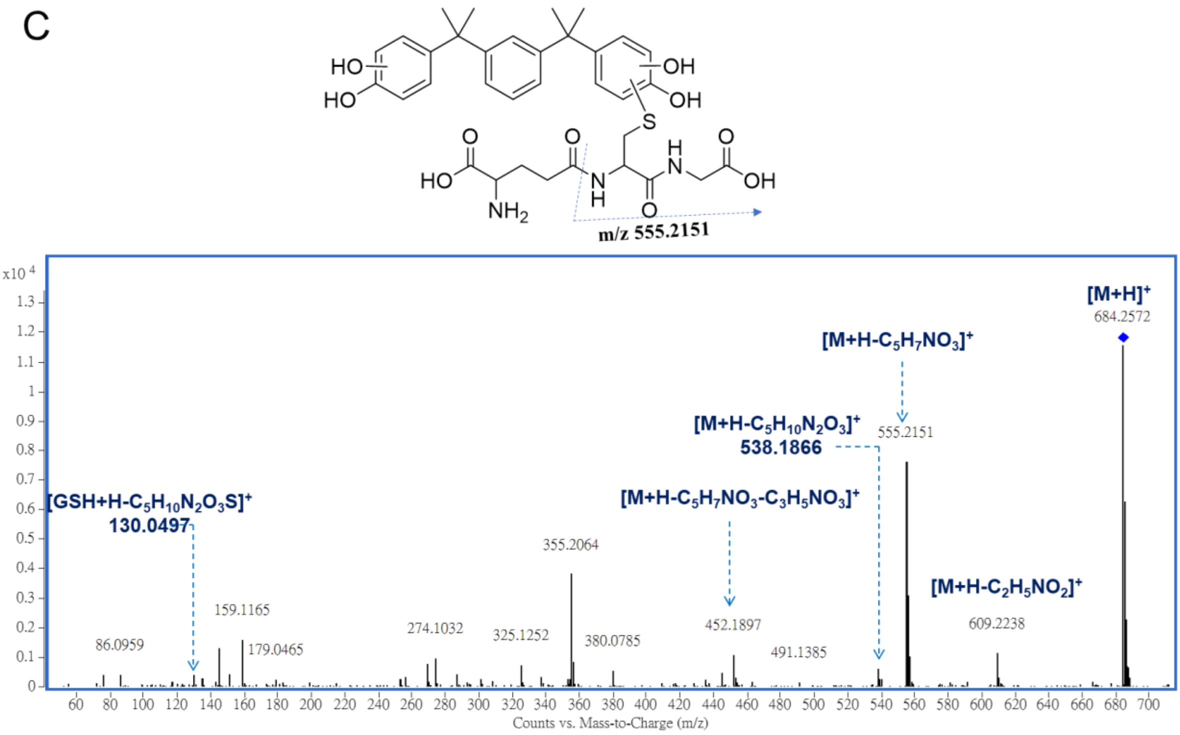

Figure S16. MS/MS spectrum and possible structure of BPM RM-GSH adducts in positive mode. (A) MG1, (B) MG2, (C) MG3.

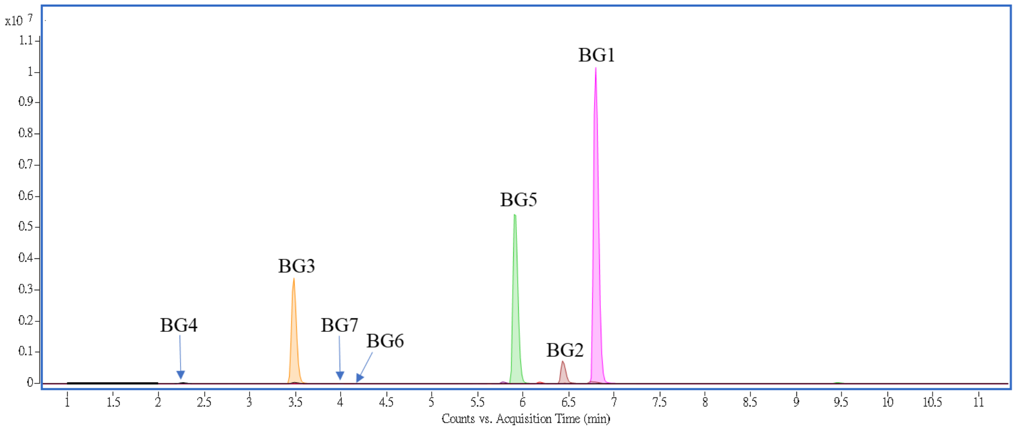

Figure S17. Extracted ion chromatograms of GSH conjugates formed with BPB and its metabolites in microsomes.

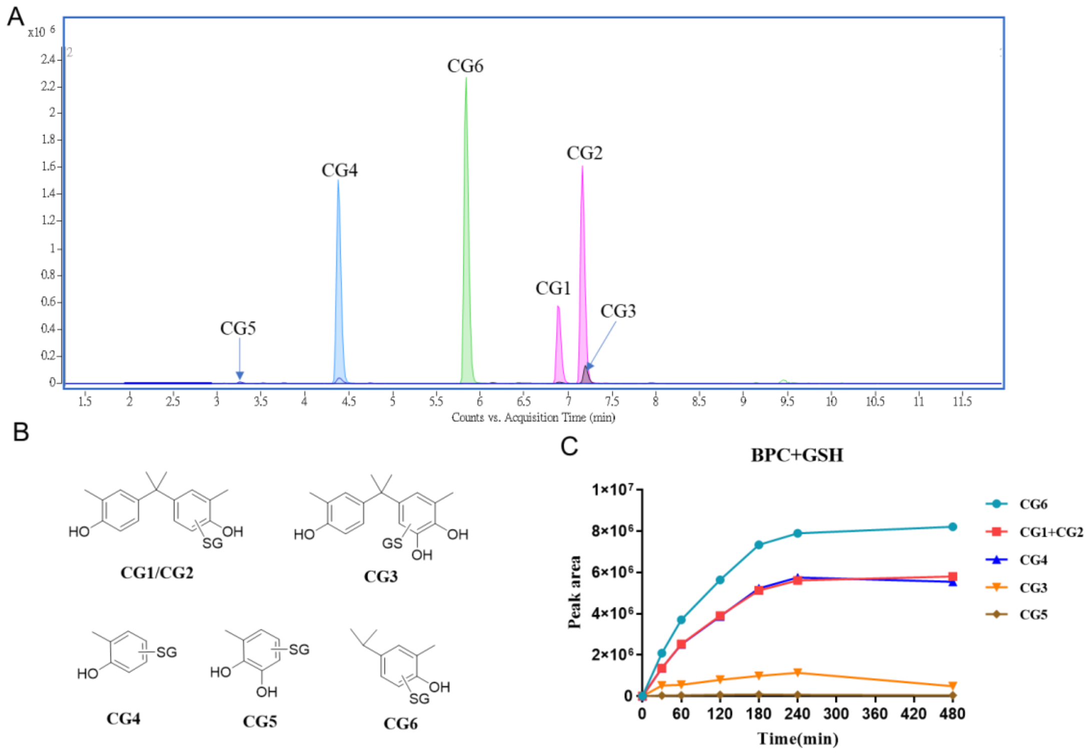

Figure S18. Extracted ion chromatograms (A), possible structure (B), and time-course changes (C) of GSH conjugates formed with BPC and its metabolites in microsomes.

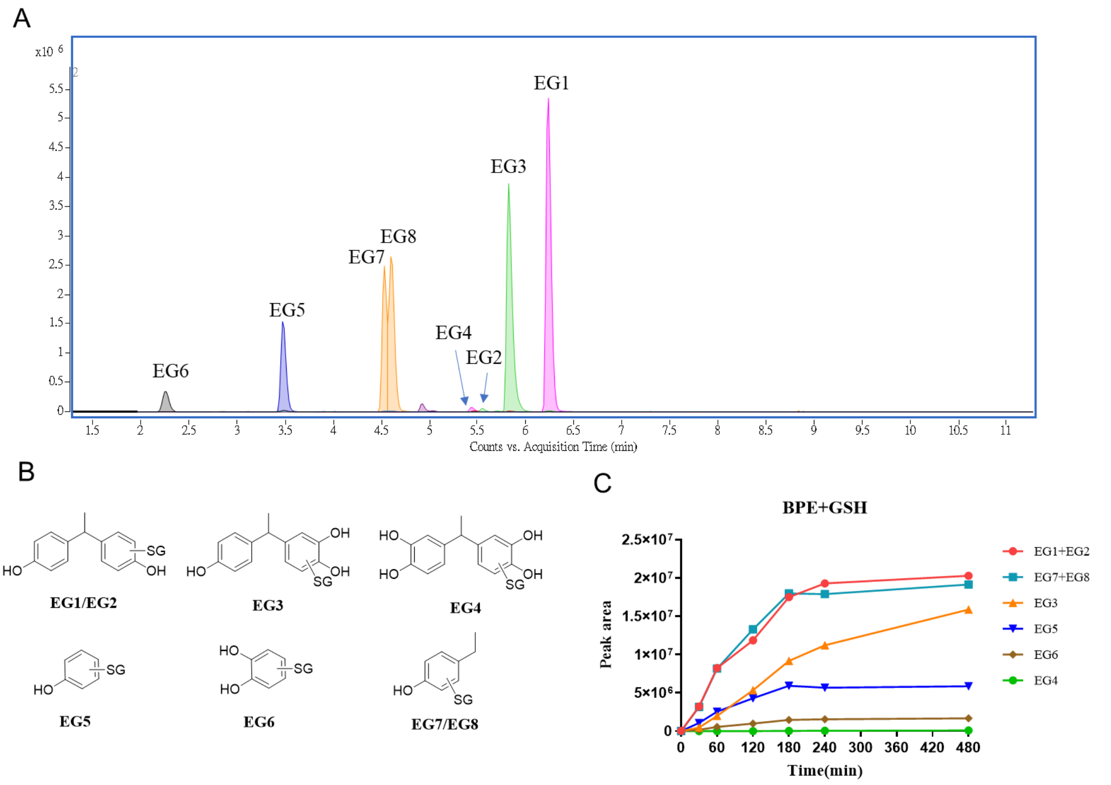

Figure S19. Extracted ion chromatograms (A), possible structure (B), and time-course changes (C) of GSH conjugates formed with BPE and its metabolites in microsomes.

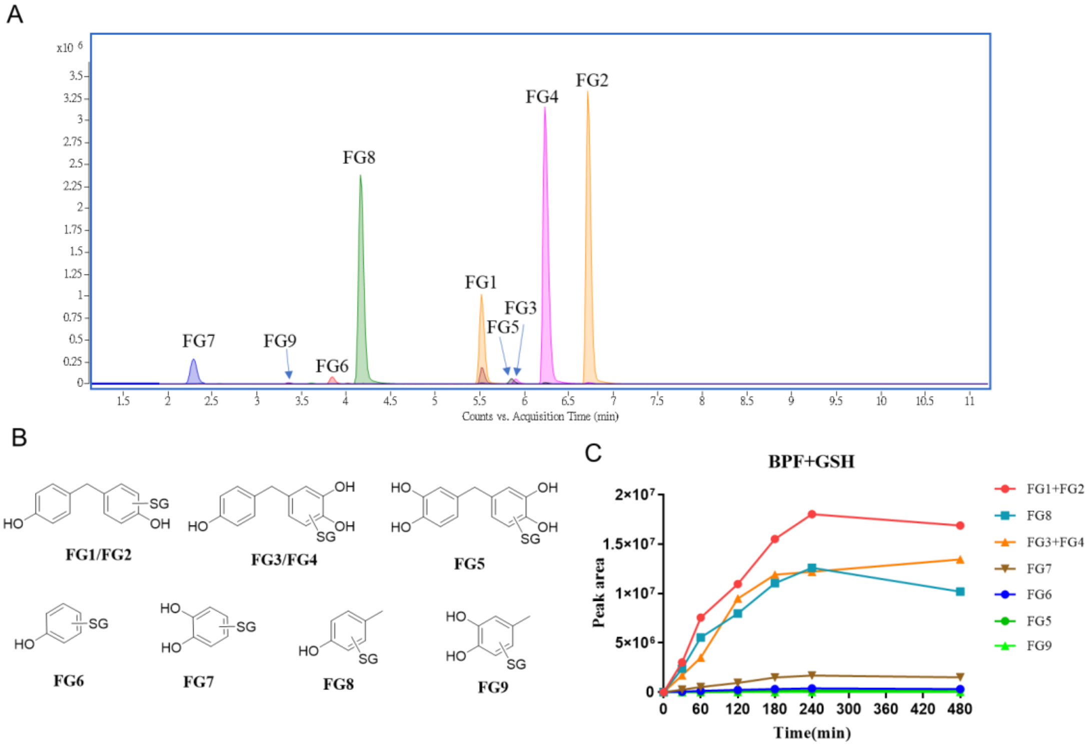

Figure S20. Extracted ion chromatograms (A), possible structure (B), and time-course changes (C) of GSH conjugates formed with BPF and its metabolites in microsomes.

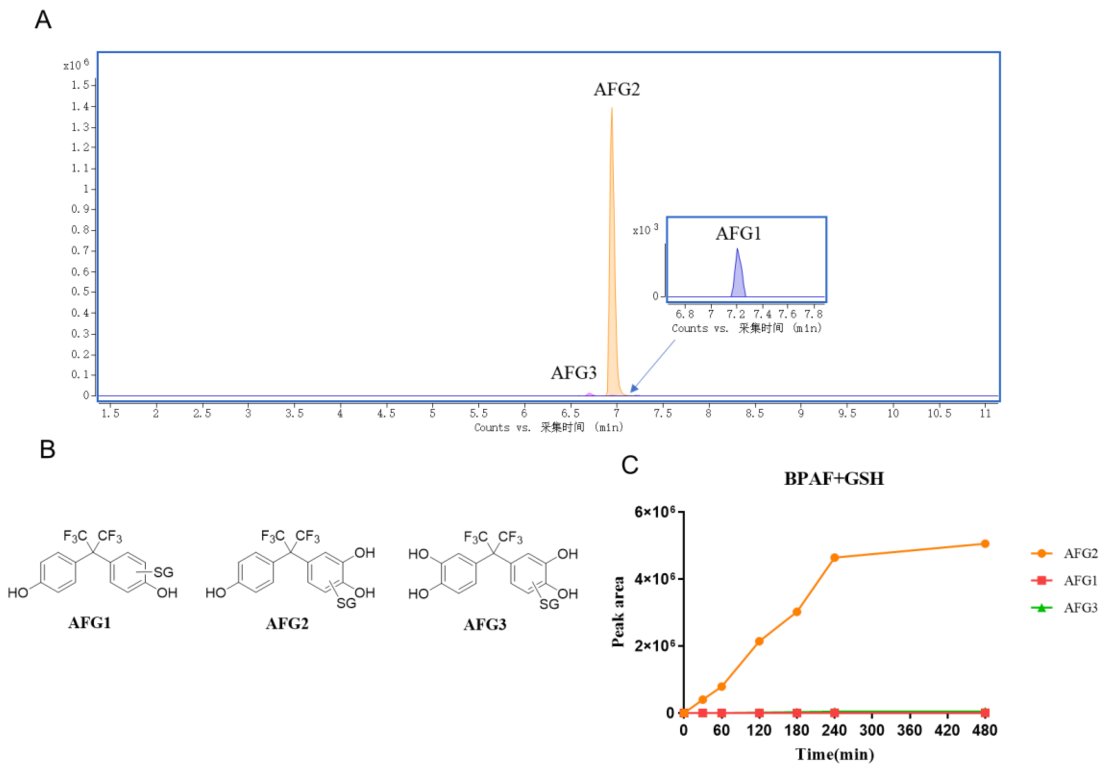

Figure S21. Extracted ion chromatograms (A), possible structure (B), and time-course changes (C) of GSH conjugates formed with BPAF and its metabolites in microsomes.

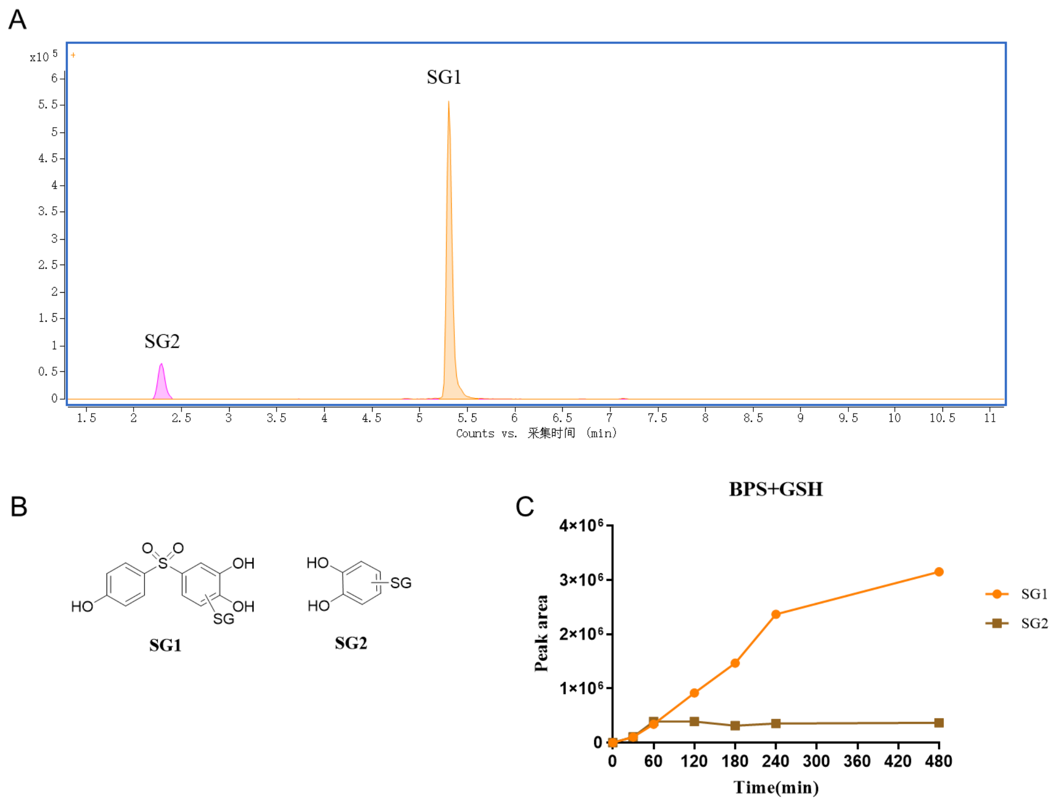

Figure S22. Extracted ion chromatograms (A), possible structure (B), and time-course changes (C) of GSH conjugates formed with BPS and its metabolites in microsomes.

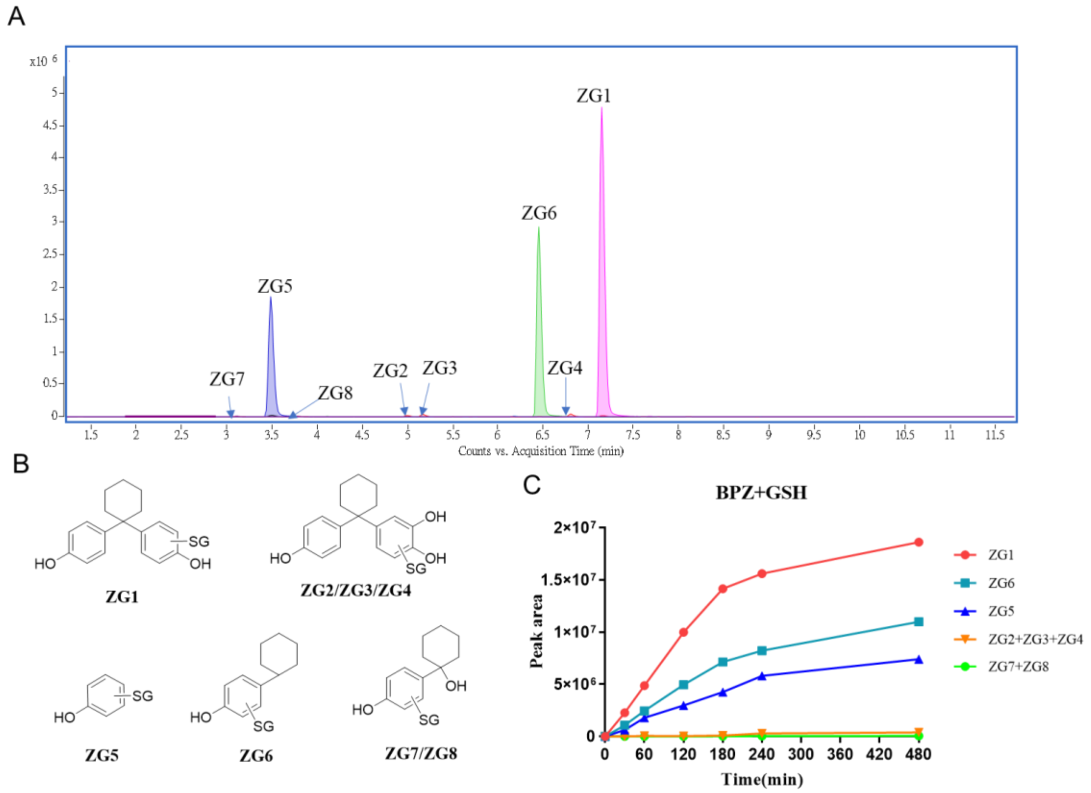

Figure S23. Extracted ion chromatograms (A), possible structure (B), and time-course changes (C) of GSH conjugates formed with BPZ and its metabolites in microsomes.

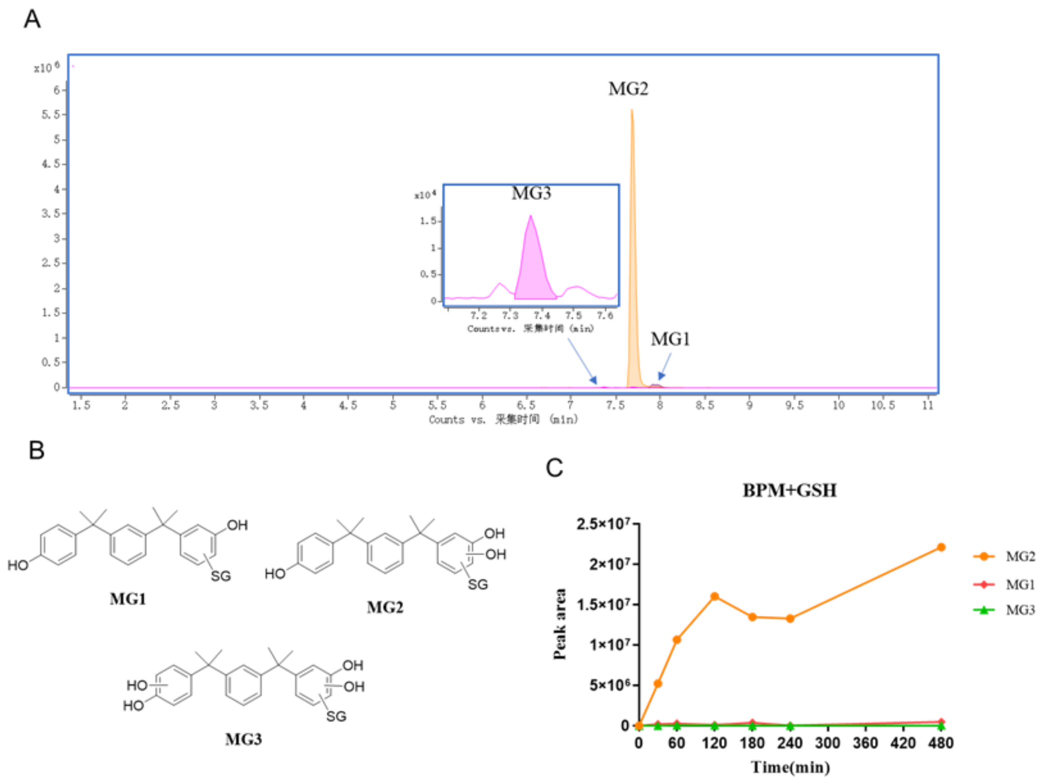

Figure S24. Extracted ion chromatograms (A), possible structure (B), and time-course changes (C) of GSH conjugates formed with BPM and its metabolites in microsomes.
